# Defining recurrent urinary tract infections and quantifying bladder cancer risk in primary care in England: a nationwide case–control study

**DOI:** 10.1016/j.lanprc.2025.100103

**Published:** 2026-02

**Authors:** Sikhuphukile G Mahati, Jianhua Wu, Fiona M Walter, Yin Zhou

**Affiliations:** a Centre for Cancer Screening, Prevention, and Early Diagnosis, Wolfson Institute of Population Health, Queen Mary University of London, London, UK; b Centre for Primary Care, Wolfson Institute of Population Health, Queen Mary University of London, London, UK

## Abstract

**Background:**

Around 30–40% of people with bladder cancer in England present with symptoms or diagnosis of urinary tract infection (UTI) in the year before a diagnosis of bladder cancer. National Institute for Health and Care Excellence guidelines advise referral of people aged 60 or older with unexplained recurrent UTIs to specialists, but recurrence is ambiguously defined, risking delayed diagnosis and worsened outcomes. We aimed to better define and quantify the effect of recurrent UTIs on the likelihood of bladder cancer.

**Methods:**

In this nationwide retrospective case–control study, we collected data from people aged 18 years or older with bladder cancer diagnosed between Jan 5, 1998, and Dec 31, 2018, and who had at least one UTI before diagnosis from the National Cancer Registration and Analysis Service (NCRAS) and the Clinical Practice Research Datalink (CPRD) in England. NCRAS diagnosis and dates were preferentially used. We matched each patient with bladder cancer (ie, case) to five patients without bladder cancer (ie, controls) using age at cancer diagnosis, sex, and general practice. Primary analyses examined the associations between UTI frequency (one, two, three, four, and five or more episodes) and bladder cancer using conditional logistic regression, within predefined look-back intervals up to 5 years before the index UTI (eg, the most recent UTI before their cancer diagnosis).

**Findings:**

We identified 92 277 patients registered with English primary care practices contributing to CPRD and 13 227 contributing to NCRAS, including 17 584 patients with bladder cancer (cases) and 87 920 without bladder cancer (controls). After excluding 7513 duplicates and 42 526 controls who had no UTIs before a diagnosis, then further excluding 1529 patients (427 cases and 1102 controls) who had a UTI 5–10 years before the index UTI, we included 53 936 patients for analysis (17 157 cases and 36 779 controls). There were more male (9992 [58·2%]) than female (7165 [41·8%]) patients with bladder cancer; with a median age of 75·0 years (IQR 67·0–81·0); and patients were White (16 028 [93·4%]), Asian (189 [1·1%]), Black (139 [0·8%]), or mixed race (31 [0·2%]) and of other (91 [0·5%]) and unknown ethnic groups (679 [4·0%]). There was a higher proportion of female than male patients without bladder cancer (19 858 [54·0%] *vs* 16 921 [46·0%]). A dose–response relationship between recurrent UTI and bladder cancer risk was seen in the first 6 months only, with the largest effect for five or more UTIs at 0–6 months (adjusted odds ratio 13·05 [95% CI 11·60–14·68]); patients with five or more UTIs had around 2·5-times higher odds than those with three UTIs in the same interval (4·95 [4·51–5·43]). Associations attenuated with increasing time from the index UTI.

**Interpretation:**

Our data suggest that recurrent UTIs within 6 months were a strong signal of bladder cancer risk. Our findings can help to refine existing guideline recommendations for patients with recurrent UTIs who might benefit from further investigations to rule out possible bladder cancer.

**Funding:**

Barts Charity and the National Institute for Health Research Policy Research Unit in Cancer Awareness, Screening, and Early Diagnosis.

## Introduction

Bladder cancer is among the ten most common cancers in England, with 18 000 new diagnoses each year and more than 150 000 people living with the disease.^1^ Early diagnosis is associated with improved survival and patient-reported outcomes;^2^ however, timely detection of bladder cancer can be challenging as urinary symptoms are common and can be caused by other benign conditions, such as urinary tract infections (UTIs) and benign prostatic disease.

**Research in context**

**Evidence before this study**

There was no formal literature search done for this study. Our previous work (as part of the PhD of the corresponding author) identified significant inequality in diagnosis and outcomes for patients with bladder cancer presenting with recurrent UTIs and other symptoms. Patients with recurrent urinary tract infections (UTIs) who are subsequently diagnosed with bladder cancer have delayed diagnosis and worse stage at diagnosis and patient experience than those presenting with haematuria, which might be partly due to the ambiguity in the definition of recurrence (currently based on clinical consensus) and insufficient knowledge about the exact risk of recurrent UTIs in patients with bladder cancer.

**Added value of this study**

Our study improves understanding of the definition of recurrent UTIs in the context of bladder cancer. We found that multiple UTIs within 6 months were a strong signal for bladder cancer risk, with a dose–response relationship between frequency of UTIs and cancer risk, especially in female patients. Our findings suggest that 6 months is the optimal interval to define recurrence of UTIs in the context of bladder cancer. This result provides a clearer definition of the concept of recurrent UTIs, which is necessary for improving clinical management of this group of patients and to inform future research to identify and risk-stratify symptomatic patients at risk of bladder cancer.

**Implications of all the available evidence**

Our study provides evidence to refine current clinical definitions of recurrent UTIs in patients with bladder cancer, which can improve clinical recommendations and pathways aiming to identify and investigate patients at risk of bladder cancer. Our findings can also inform future research that aims to better understand cancer risk in patients with recurrent UTIs in the general population and develop risk stratification approaches to guide investigations in these patients to detect bladder cancer earlier.

Existing evidence shows that 30–40% of people with bladder cancer report symptoms or diagnosis of urinary tract infection in the year before a diagnosis of bladder cancer.^3^^,^^4^ In people with bladder cancer, antibiotic prescriptions for UTIs often increase 7–11 months before a diagnosis in primary care, suggesting that these symptoms commonly occur a long time before the cancer diagnosis.^5^ Although a single UTI presentation signals low risk for cancer, repeat presentations might indicate a higher than average risk.^6^ National Institute for Health and Care Excellence (NICE) guidelines for suspected cancer (NG12) suggest that general practitioners should consider referring people aged 60 or older who have repeated episodes of UTI to a specialist for a cystoscopy, to rule out bladder cancer.^7^ However, referral happens for approximately a quarter of those with recurrent UTIs,^8^ partly due to ambiguity around the definition of recurrence and the likely low diagnostic yield of cancer in patients with recurrent UTIs.^9^ Consequently, people with bladder cancer (especially female patients) presenting with UTIs or similar symptoms have a longer time to diagnosis; are less likely to be referred; and have worse patient experiences, stage at diagnosis, and survival than those presenting with haematuria.^4^^,^^10^, ^11^, ^12^, ^13^

The current definition of recurrent UTIs is largely based on clinical consensus, with the European Urological Association defining recurrence as two episodes in 6 months, or three episodes in 1 year.^9^ In primary care, NICE guidelines (NG112) also advise general practitioners to consider empirical antibiotics for patients with two or more UTI symptoms (including dysuria, urinary frequency, and urgency) without requiring culture-confirmed infection.^14^^,^^15^ Although these irritative bladder symptoms without the presence of infection might also be an underlying sign of bladder cancer, identification of patients at high risk of bladder cancer is challenging in primary care. Given insufficient knowledge of the definition of recurrent UTIs and their effects on bladder cancer risk, clinical management and subsequent follow-up are suboptimal in primary care, with many patients who have symptoms of recurrent UTIs being given repeated courses of antibiotics and few clinical reviews.^8^

We aimed to better define and quantify the effect of recurrent UTIs on the likelihood of bladder cancer. Recognising that many UTIs are managed without definitive urine cultures in primary care, we sought to distinguish patterns that are consistent with genuine infection (ie, clinical UTIs) from recurrent irritative symptoms that mimic infection. Our objectives were to identify a time interval within which repeated UTI episodes most strongly signal bladder cancer risk; quantify the odds associated with episode counts and look-back intervals; and estimate the likelihood of bladder cancer in patients who have recurrent clinical UTIs and in those with recurrent irritative symptoms without clinical UTIs.

## Methods

### Study design and participants

In this nationwide retrospective case–control study, we collected data from people aged 18 years or older with bladder cancer diagnosed between Jan 5, 1998, and Dec 31, 2018, and who had at least one UTI before diagnosis from the National Cancer Registration and Analysis Service (NCRAS) and the Clinical Practice Research Datalink (CPRD) in England. The CPRD contains coded primary care data for more than 65 million patients, including 19 million who were registered as of 2025 in England.^16^ These data are broadly representative and cover around 20% of the UK population.^17^^,^^18^ The CPRD data are linked at source to Hospital Episodes Statistics (HES) datasets, the English Cancer Registry, and patient-level Index of Multiple Deprivation. HES contains secondary data from English National Health Service (NHS) hospitals (used exclusively for the extraction of ethnicity data in this study); Cancer Registry data are considered the gold standard for cancer diagnostic information in epidemiological research. CPRD approval was obtained after a Research Data Governance application for the study protocol (22_002092).

People were identified from NCRAS using ICD-10 codes and from CPRD using medcodes (appendix pp 16–17). NCRAS diagnosis and dates were preferentially used. Five patients without bladder cancer (ie, controls) from the general CPRD population were matched to each bladder cancer case based on age at cancer diagnosis, sex (male or female), and general practice. We additionally matched each patient with bladder cancer (ie, case) to their five controls based on the period of primary care data that were available before a cancer diagnosis, to ensure we could capture UTI events over a similar time period for both cases and controls. Consent was waived as data from CPRD do not contain identifiable patient information.

### Procedures

As most UTIs in primary care are diagnosed presumptively and before a urine culture is performed or available, we considered all recorded relevant UTI events in CPRD (including a UTI diagnosis, symptoms and signs of UTIs, and prescriptions for commonly used antibiotics to treat UTIs; appendix pp 6–7). Codes from previously published studies^4^^,^^5^^,^^19^ were reviewed by a general practitioner (YZ, who has 15 years of clinical experience in English primary care) and verified with a senior general practitioner (FMW, with more than 30 years of clinical experience in primary care in England) when necessary. We included codes that are most indicative of probable UTI that would warrant primary care management including those for irritative urinary symptoms (ie, dysuria, urinary frequency, and urinary urgency) and signs of probable UTI (ie, positive urine dipstick for leukocytes, or nitrites) and prescriptions that allowed reliable inference that they were prescribed for a UTI (ie, trimethoprim, nitrofurantoin, pivmecillinam, fosfomycin, methenamine hippurate, and sodium citrate; appendix pp 21–26). Although methenamine hippurate is commonly used to treat patients with established recurrent UTIs, and sodium citrate is often bought over the counter, we included these to enable an all-inclusive approach at identifying patients most likely to suffer from recurrent UTIs. We treated event codes that occurred on the same day as one event, prioritising events by the type of diagnosis, prescriptions, then symptoms and signs (in this order).

For each cancer case, we identified the most recent UTI before their cancer diagnosis (index UTI; time 0) and constructed look-back intervals over the preceding 10 years. Because UTI frequency plateaued after 5 years for both cases and controls (appendix p 2), subsequent analyses were restricted to the 5 years before the index UTI. Within each interval, we counted the frequency of UTIs (including index UTI [one episode] and two, three, four, and five or more episodes; appendix p 3). Controls were anchored on the same timeline as their matched case (ie, analysis intervals were defined retrospectively from the date of the index UTI [ie, time 0] for both cases and controls, appendix p 3).

Age group at diagnosis (≤40, 41–50, 51–60, 61–70, 71–80, or >80 years) and sex (male or female) were obtained from NCRAS and race and ethnicity (White, Asian, Black, or mixed race and other or unknown ethnic groups) was obtained from HES. Deprivation, measured by Index of Multiple Deprivation quintiles, was obtained from the linked CPRD dataset. Clinical risk factors, such as smoking status (non-smoker, current, ex-smoker, or unknown; most recent smoking status recorded at any point before a cancer diagnosis) and BMI (underweight [<18·5 kg/m^2^], healthy [18·5–24·9 kg/m^2^], overweight [25–29·9 kg/m^2^], obese [>30 kg/m^2^], or unknown; standardised international thresholds), were obtained from coded information within CPRD. The data sources and derivation of each variable are listed in the appendix (pp 8–9).

We considered comorbidities that might predispose patients to getting UTIs and irritative urinary symptoms (ie, diabetes and multiple sclerosis),^20^^,^^21^ use of hormone replacement therapy (recommended by NICE for atrophic vaginitis that can predispose and mimic symptoms of UTIs),^14^ and conditions in which UTIs are common but might affect help-seeking or access to health care (ie, dementia, stroke, and learning disability).^22^

All covariates were determined using the latest recorded data available at any point before cancer diagnosis, except for BMI which was determined using data recorded in the 2 years before a diagnosis to allow more reliable examination of the association between BMI and cancer risk, as BMI is more likely to fluctuate over time than other covariates (appendix pp 8–9).

### Statistical analysis

Primary analyses examined the associations between UTI frequency and bladder cancer using conditional logistic regression, accounting for the matched design (age, sex, and general practice). We fitted univariable models and three multivariable models to examine robustness to covariate adjustment, including the sociodemographic model (adjusted for ethnicity and Index of Multiple Deprivation quintiles), the clinical model (adjusted for BMI, smoking status, and comorbidities), and the combined model (adjusted for sociodemographic and clinical covariates). UTI frequency was entered as a categorical exposure within each prespecified look-back interval to allow for non-linearity and dose–response assessment.

For covariates with missing values, we retained all individuals in the analysis by including a separate unknown category for each variable, so that any systematic differences in risk among those with missing data could be captured as a separate group.

Secondary analyses compared signals from putative infections (defined by diagnosis or prescription codes) with irritative symptoms (defined by codes for symptoms or signs of UTI). We repeated all analyses separately for UTI events (ie, diagnoses, prescriptions, or symptoms or signs). We assumed decreasing clinical certainty of a UTI event (diagnoses > prescriptions > symptoms or signs) and, because prescriptions and diagnoses might reflect similar certainty of a clinical UTI, we additionally analysed a combined clinical UTI definition (ie, diagnoses and prescriptions only). For each definition, recurrence counts included only episodes of the same type.

We also performed three sensitivity analyses. First, we examined whether results in the period closest to diagnosis were sensitive to changes in interval lengths. We respecified the first year before the index UTI as four consecutive 3-month intervals (instead of two 6-month intervals in the main analysis) and re-estimated all conditional logistic regression models. Next, we assessed whether persistent or treatment-resistant UTIs affected the observed patterns in the main analysis by considering UTI episodes occurring within a 14-day period as one event. As there is a strong sex imbalance for bladder cancer incidence, diagnosis, and survival,^1^^,^^10^^,^^12^ we additionally repeated the main analyses stratified by sex.

Analyses were conducted using Stata (version 18), with the clogit command used for conditional logistic regression.^23^

### Role of the funding source

The funders of the study had no role in study design, data collection, data analysis, data interpretation, or writing of the report.

## Results

**Figure 1:**
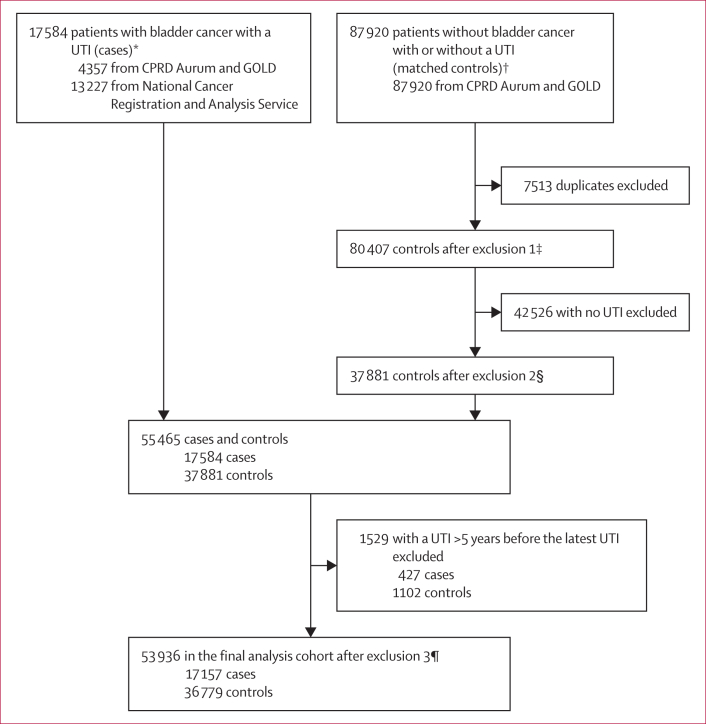
Study flow diagram CPRD=Clinical Practice Research Datalink. UTI=urinary tract infection. ∗Diagnosis date from Jan 5, 1998, to Dec 31, 2018. Index UTI date from Jan 17, 1990, to Dec 21, 2018. †Index UTI date from Jan 1, 1988, to Jan 19, 2024. Five matched controls for one case (matched on age, sex, and practice ID). ‡In exclusion 1, controls were matched to multiple cases and duplicates removed by retaining the control linked to the earliest case diagnosis date. §In exclusion 2, controls without a UTI were excluded. ¶In exclusion 3, cases and controls who had a UTI more than 5 years since the last UTI diagnosis were excluded.

We collected data for 92 277 patients registered with English primary care practices contributing to CPRD and 13 227 patients in NCRAS, including 17 584 patients with bladder cancer (cases) and 87 920 without bladder cancer (controls; figure 1). We excluded 7513 duplicates and 42 526 controls (male 28 450 [66·9%] and female 14 076 [33·1%]; mean age 71·5 years [SD 11·5]) who had no UTIs before a diagnosis, resulting in 55 465 patients in total (17 584 cases and 37 881 controls) with at least one UTI occurring up to 10 years before a diagnosis. After excluding 1529 patients (427 cases and 1102 controls) who had a UTI 5–10 years before the index UTI, we included 53 936 for analysis (17 157 cases and 36 779 controls).

There were more male (9992 [58·2%]) than female (7165 [41·8%]) patients with bladder cancer, with a median age of 75·0 years (IQR 67·0–81·0). Patients were White (16 028 [93·4%]), Asian (189 [1·1%]), Black (139 [0·8%]), and mixed race (31 [0·2%]) and of other (91 [0·5%]) and unknown ethnic groups (679 [4·0%]; table 1). There was a higher proportion of female than male patients without bladder cancer (controls; 19 858 [54·0%] *vs* 16 921 [46·0%]), but the age and ethnicity distributions were similar to those with bladder cancer. 10 053 (58·6%) cases compared with 16 069 (43·7%) controls were current or ex-smokers. A higher proportion of cases had diabetes (2879 [16·8%] *vs* 5104 [13·9%]) and neurological conditions (1506 [8·8%] *vs* 2912 [7·9%]) than controls. Hormone replacement therapy was used by 6568 (17·9%) controls and 2251 (13·1%) cases. Mean BMI were similar (27·3 [SD 8·4] in cases *vs* 27·5 [8·0] in controls).

**Table 1:** Baseline characteristics

	Patients with bladder cancer (cases; n=17 157)	Patients without bladder cancer (matched controls; n=36 779)	Total (n=53 936)
Sex			
Male	9992 (58·2%)	16 921 (46·0%)	26 913 (49·9%)
Female	7165 (41·8%)	19 858 (54·0%)	27 023 (50·1%)
Age, years			
Mean	73·2 (11·2)	74·3 (11·1)	73·9 (11·1)
Median	75·0 (67·0–81·0)	76·0 (68·0–82·0)	76·0 (68·0–82·0)
Age group at diagnosis, years			
≤40	170 (1·0%)	338 (0·9%)	508 (0·9%)
41–50	497 (2·9%)	945 (2·6%)	1442 (2·7%)
51–60	1566 (9·1%)	2753 (7·5%)	4319 (8·0%)
61–70	3857 (22·5%)	7511 (20·4%)	11 368 (21·1%)
71–80	6214 (36·2%)	13 495 (36·7%)	19 709 (36·5%)
>80	4853 (28·3%)	11 737 (31·9%)	16 590 (30·8%)
Race and ethnicity			
White	16 028 (93·4%)	33 846 (92·0%)	49 874 (92·5%)
Asian	189 (1·1%)	564 (1·5%)	753 (1·4%)
Black	139 (0·8%)	363 (1·0%)	502 (0·9%)
Mixed	31 (0·2%)	74 (0·2%)	105 (0·2%)
Other	19 (0·5%)	215 (0·6%)	306 (0·6%)
Unknown	679 (4·0%)	1717 (4·7%)	2396 (4·4%)
Index of Multiple Deprivation			
1 (least deprived)	3687 (21·5%)	8680 (23·6%)	12 367 (22·9%)
2	3926 (22·9%)	8276 (22·5%)	12 202 (22·6%)
3	3389 (19·8%)	7468 (20·3%)	10 857 (20·1%)
4	3188 (18·6%)	6657 (18·1%)	9845 (18·3%)
5 (most deprived)	2953 (17·2%)	5658 (15·4%)	8611 (16·0%)
Unknown	14 (0·1%)	40 (0·1%)	54 (0·1%)
Smoking status∗			
Non-smoker	6102 (35·6%)	18 115 (49·3%)	24 217 (44·9%)
Current smoker	5817 (33·9%)	7735 (21·0%)	13 552 (25·1%)
Ex-smoker	4236 (24·7%)	8334 (22·7%)	12 570 (23·3%)
Unknown	1002 (5·8%)	2595 (7·1%)	3597 (6·7%)
Comorbidities∗			
Diabetes (any)	2879 (16·8%)	5104 (13·9%)	7983 (14·8%)
Type 1	191 (1·1%)	161 (0·4%)	352 (0·7%)
Type 2	2812 (16·4%)	5013 (13·6%)	7825 (14·5%)
Neurological (any)	1506 (8·8%)	2912 (7·9%)	4418 (8·2%)
Dementia	529 (3·1%)	1120 (3·0%)	1649 (3·1%)
Learning disability	51 (0·3%)	69 (0·2%)	120 (0·2%)
Multiple sclerosis	77 (0·4%)	149 (0·4%)	226 (0·4%)
Stroke	914 (5·3%)	1725 (4·7%)	2639 (4·9%)
Hormone replacement therapy use†	2251 (13·1%)	6568 (17·9%)	8819 (16·4%)
BMI∗, kg/m^2^			
Mean	27·3 (8·4)	27·5 (8·0)	27·4 (8·2)
Median	26·5 (23·5–30·1)	26·7 (23·9–30·1)	26·7 (23·8–30·1)
BMI group, kg/m^2^			
Underweight (<18·5)	400 (2·3%)	526 (1·4%)	926 (1·7%)
Healthy (18·5–24·9)	3636 (21·2%)	8060 (21·9%)	11 696 (21·7%)
Overweight (25·0–29·9)	4137 (24·1%)	9915 (27·0%)	14 052 (26·1%)
Obese (>30·0)	2831 (16·5%)	6637 (18·0%)	9468 (17·6%)
Unknown	6153 (35·9%)	11 641 (31·7%)	17 794 (33·0%)

Data are n (%), mean (SD), and median (IQR).

∗ Smoking status, comorbidities, and BMI (up to 2 years before a diagnosis) were obtained before the date of a cancer diagnosis for all cases and from before the same date for matched controls.

† Defined as the presence of a recorded prescription for hormone replacement therapy before the date of a cancer diagnosis for all cases and from before the same date for matched controls.

Cases were more likely to have recurrent UTIs in the year before a cancer diagnosis than controls (appendix p 3). A higher frequency of UTIs was observed in the year before a cancer diagnosis in cases, which was not observed in controls (appendix p 4). The increased frequency of UTIs was concentrated over a 6-month interval in cases and controls.

**Figure 2:**
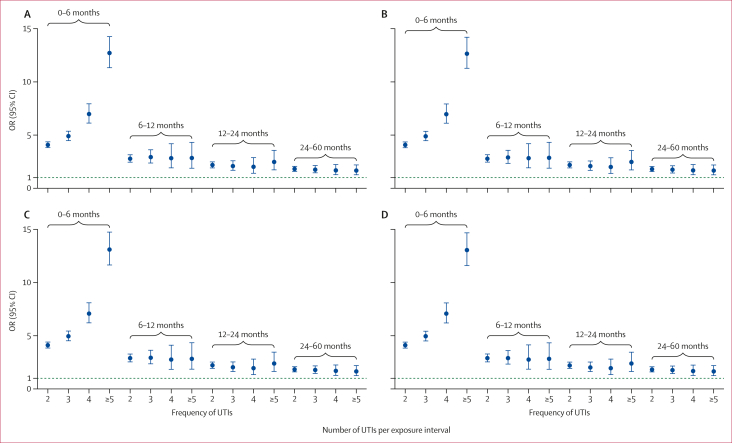
Unadjusted and adjusted ORs for bladder cancer by rate of UTI during a 5-year period ORs that are unadjusted (A), adjusted for sociodemographic factors including ethnicity and Index of Multiple Deprivation (B), adjusted for clinical risk factors including BMI, smoking status, and comorbidities (C), and fully adjusted for sociodemographic and clinical risk factors (D). The horizontal dashed line indicates OR of 1, which is the reference group in each model. OR=odds ratio. UTI=urinary tract infection.

Across all look-back intervals up to 5 years, having two or more UTI episodes was associated with higher odds of bladder cancer versus a single UTI in univariable and multivariable models (figure 2; table 2). Associations were strongest in the 0–6-month interval and attenuated with increasing time from the index UTI. For example, among those with three UTIs, the fully adjusted odds ratio [OR] was 4·95 (95% CI 4·51–5·43) at 0–6 months, 2·90 (2·34–3·60) at 6–12 months, and 2·03 (1·63–2·53) at 12–24 months. For four UTIs, adjusted ORs were 7·08 (6·20–8·09) at 0–6 months, 2·77 (1·86–4·14) at 6–12 months, and 1·95 (1·35–2·81) at 12–24 months. A clear dose–response was seen in the first 6 months only, with the largest effect for five or more UTIs at 0–6 months (adjusted OR 13·05 [11·60–14·68]); patients with five or more UTIs had around 2·5-times higher odds than those with three UTIs in the same interval (figure 2; appendix pp 10–11).

**Table 2:** Adjusted ORs and 95% CIs for the association between rate of UTI and bladder cancer for main analyses, UTI codes, and UTI-like codes

	Fully adjusted main analysis (95% CI)	n/N (%)	Adjusted OR (95% CI) in model of UTI diagnosis events	n (%)	Adjusted OR (95% CI) in model of UTI prescription events	n (%)	Adjusted OR (95% CI) in model of UTI symptoms or signs	n (%)	Adjusted OR (95% CI) in model of UTI diagnosis or prescription events
1 UTI∗	1 (ref)	4934/20 781 (23·7%)	1 (ref)	1417/8108 (17·5%)	1 (ref)	1133/4085 (27·7%)	1 (ref)	6351/28 889 (22·0%)	1 (ref)
**0–6 months**									
2 UTIs	4·11 (3·84–4·40)	2225/4591 (48·5%)	3·86 (3·54–4·23)	486/857 (56·7%)	4·50 (3·61–5·62)	463/910 (50·9%)	3·11 (2·20–4·39)	2711/5448 (49·8%)	4·27 (3·98–4·59)
3 UTIs	4·95 (4·51–5·43)	1124/2236 (50·3%)	5·73 (5·00–6·57)	261/517 (50·5%)	4·40 (2·93–6·61)	251/434 (57·8%)	4·17 (2·27–7·68)	1385/2753 (50·3%)	5·95 (5·32–6·65)
4 UTIs	7·08 (6·20–8·09)	600/1039 (57·7%)	9·81 (7·94–12·14)	112/198 (56·6%)	8·32 (4·14–16·77)	126/202 (62·4%)	10·85 (2·26–51·95)	712/1237 (57·6%)	9·10 (7·71–10·74)
≥5 UTIs	13·05 (11·60–14·68)	1064/1619 (65·7%)	15·36 (12·63–18·69)	252/398 (63·3%)	6·94 (3·89–12·39)	180/255 (70·6%)	6·91 (1·88–25·61)	1316/2017 (65·3%)	15·08 (12·95–17·57)
**6–12 months**									
2 UTIs	2·89 (2·54–3·29)	403/1108 (36·4%)	2·55 (2·16–3·01)	104/221 (47·1%)	3·50 (2·42–5·06)	79/162 (48·8%)	1·73 (0·92–3·25)†	507/1329 (38·2%)	2·81 (2·46–3·21)
3 UTIs	2·90 (2·34–3·60)	130/355 (36·6%)	2·74 (1·92–3·92)	33/102 (32·4%)	1·29 (0·43–3·93)†	23/62 (37·1%)	1·92 (0·43–8·51)†	163/457 (35·7%)	3·14 (2·36–4·18)
4 UTIs	2·77 (1·86–4·14)	40/110 (36·4%)	2·73 (1·46–5·12)	8/24 (33·3%)	0·81 (0·07–9·78)†	7/22 (31·8%)	4·28 (0·24–77·52)†	48/134 (35·8%)	3·54 (2·15–5·83)
≥5 UTIs	2·83 (1·85–4·33)	31/109 (28·4%)	1·95 (0·87–4·39)†	19/35 (54·3%)	··	3/7 (42·9%)	··	50/144 (34·7%)	2·97 (1·63–5·41)
**12–24 months**									
2 UTIs	2·21 (1·93–2·52)	352/1065 (33·1%)	2·13 (1·79–2·53)	94/225 (41·8%)	2·16 (1·43–3·25)	59/162 (36·4%)	1·97 (1·05–3·69)	446/1290 (34·6%)	2·23 (1·94–2·57)
3 UTIs	2·03 (1·63–2·53)	116/407 (28·5%)	2·30 (1·62–3·26)	37/113 (32·7%)	2·36 (0·88–6·35)†	29/63 (46·0%)	8·45 (0·66–107·82)†	153/520 (29·4%)	2·27 (1·73–2·98)
4 UTIs	1·95 (1·35–2·81)	44/142 (31·0%)	1·64 (0·95–2·83)†	9/33 (27·3%)	··	4/19 (21·1%)	··	53/175 (30·3%)	1·73 (1·10–2·72)
≥5 UTIs	2·38 (1·64–3·45)	37/144 (25·7%)	2·42 (1·30–4·50)	9/32 (28·1%)	··	6/18 (33·3%)	··	46/176 (26·1%)	3·02 (1·81–5·04)
**24–60 months**									
2 UTIs	1·82 (1·60–2·08)	333/1142 (29·2%)	1·60 (1·35–1·90)	84/220 (38·2%)	2·75 (1·89–4·00)	65/197 (33·0%)	2·16 (1·14–4·09)	417/1362 (30·6%)	1·79 (1·57–2·04)
3 UTIs	1·76 (1·45–2·14)	128/505 (25·4%)	1·52 (1·11–2·07)	43/131 (32·8%)	1·88 (0·93–3·79)†	15/72 (20·8%)	2·06 (0·34–12·62)†	171/636 (26·9%)	1·81 (1·43–2·29)
4 UTIs	1·69 (1·27–2·25)	69/238 (29·0%)	2·53 (1·66–3·86)	15/49 (30·6%)	3·11 (0·65–14·94)†	13/40 (32·5%)	··	84/287 (29·3%)	2·38 (1·67–3·39)
≥5 UTIs	1·66 (1·25–2·20)	64/261 (24·5%)	2·21 (1·37–3·56)	14/72 (19·4%)	··	10/39 (25·6%)	··	78/333 (23·4%)	2·04 (1·41–2·95)

All models were adjusted for ethnicity, deprivation (Index of Multiple Deprivation quintiles), BMI, smoking status, and comorbidities (diabetes, neurological conditions, and use of hormone replacement therapy). OR=odds ratio. UTI=urinary tract infection.

∗ Reference group for the exposure time intervals.

† Not statistically significant at the 95% CI level.

In the adjusted analysis, White ethnicity (adjusted OR 1·49 [95% CI 1·27–1·74]), higher deprivation (1·21 [1·10–1·33] for Index of Multiple Deprivation quintile 5), underweight status (1·63 [1·37–1·93] for BMI <18·5 kg/m^2^), diabetes (1·38 [1·29–1·47]), and neurological conditions (1·13 [1·04–1·23]) were each associated with modestly increased odds of bladder cancer (appendix pp 10–11). Current smokers and ex-smokers had higher odds of bladder cancer than non-smokers (2·14 [2·01–2·27] *vs* 1·50 [1·41–1·59]). Use of hormone replacement therapy appeared to be a protective factor against bladder cancer risk (0·77 [0·71–0·83]).

Across the three model specifications (sociodemographic, clinical, and combined), association patterns were consistent. The dose–response at the 0–6-month interval was preserved after adjustment, whereas effects beyond 6 months were attenuated and of similar magnitude across 6–12, 12–24, and 24–60-month intervals (figure 2). Those presenting with two episodes in 6 months had 1·5–2·0-times higher odds of bladder cancer than those with recurrent UTIs of any frequency beyond 6 months since the most recent UTI.

Among the study population, most recorded UTI events were diagnosis (35 852 [66·5%] of 53 936), prescriptions (11 335 [21·0%]), then symptoms or signs (6749 [12·5%]; appendix p 6). In cases, the breakdown of codes by type was diagnosis (11 694 [68·1%]), prescription (2997 [17·5%]), and symptoms or signs (2466 [14·4%]). In controls, the breakdown of codes by type was diagnosis (24 158 [65·7%]), prescription (8338 [22·7%]), and symptoms or signs (4283 [11·6%]). 3147 (5·8%) of 53 936 patients had two or more types of UTI codes recorded on the same day (appendix p 7).

There was a similar direction and dose relationship effect between recurrent UTIs defined by diagnosis codes and all codes (main analysis) across all look-back intervals (table 2). The effect of five or more UTIs was almost three times that of three UTIs at the 0–6 month interval for those with a recurrent UTI diagnosis (adjusted OR 15·36 [95% CI 12·63–18·69] *vs* 5·73 [5·00–6·57]). For UTIs defined by prescription events only and irritative symptoms or signs only, there was around two-times increase in the odds of bladder cancer between those with three and those with four episodes of UTIs at the 0–6 month interval in both models. Although those with five or more UTIs in this interval in these two models had a reduction in odds compared with those who had four episodes, the absolute odds of bladder cancer for those with five or more UTIs were greater than those with three UTIs at the same intervals. The overall pattern of association between rates of UTI and bladder cancer was similar for models using diagnosis or prescription events and the main analyses, showing an increasing dose-dependent relationship between the number of UTI episodes and odds of bladder cancer at the 0–6 month interval.

In the sensitivity analysis performed with 3-month intervals in the first year before the index UTI, we found an increase in the odds of bladder cancer at the 0–3 month and 3–6 month intervals in patients with two UTIs compared with those who had five or more UTIs (appendix p 5). Although the dose–response relationship was preserved at the 6–9 month interval with increasing frequency of UTIs, the increase in odds was significantly less substantial between those with two and those with five or more UTI episodes.

When considering UTI events within 14 days as one event, the overall odds of bladder cancer remained highest in the 0–6 month interval, with a dose–response relationship seen between UTI frequency and bladder cancer odds in this interval (appendix pp 12–13).

In the sex-stratified analysis, we found a similar dose–response relationship to the main analysis between frequency of UTIs and odds of bladder cancer for male and female patients with recurrent UTIs at a 6-month interval, with a stronger dose–response effect seen in females than males (adjusted OR 9·29 [7·75–11·14] *vs* 5·20 [4·28–6·32] for four episodes and 20·74 [17·67–24·34] *vs* 6·54 [5·48–7·81] for five or more episodes in the 0–6 month interval; appendix pp 14–15).

## Discussion

Our study found that repeated attendance in primary care with symptoms and diagnosis of UTIs within 6 months was a strong signal for bladder cancer risk. There is a dose–response relationship between the frequency of UTIs within 6 months and likelihood of bladder cancer, especially in female patients. Smokers and ex-smokers, people who were White, those who were underweight (BMI <18·5 kg/m^2^), and those who were in the most deprived quintile had increased odds of having bladder cancer. The use of hormone replacement therapy appeared to be a protective factor for bladder cancer in patients with recurrent UTIs. Recurrent irritative urinary symptoms within 6 months were also associated with increased odds of bladder cancer. The overall effect sizes of the clinical and sociodemographic factors were small compared with the effect of a higher number of UTI episodes.

Our findings substantiate previous evidence that recurrent UTIs are associated with increased risk of bladder cancer,^24^^,^^25^ including the risk of squamous cell carcinoma of the bladder.^26^ Although previous associations between frequency of UTIs and bladder cancer risk have been mixed,^24^^,^^25^ to our knowledge, this study is the largest and the first to show a clear dose-relationship between the frequency of UTIs within predefined time intervals and bladder cancer risk. Further, our study provides more granular details than previous work regarding subgroup variations and considers risk factors and covariates that have not been previously examined. Our findings relating to the associations between smoking, deprivation, and bladder cancer risk corroborate existing evidence.^27^

To our knowledge, this study is the first to use a large national linked dataset to quantify the effect of recurrent UTIs in primary care on bladder cancer risk. We used a wide definition of recurrent UTIs, including clinically diagnosed UTIs and irritative symptoms, which might be managed similarly in primary care. In addition to clinical codes, we also used prescription data that are well recorded and less affected by coding biases, which allowed us to ascertain likely UTI presentations in primary care with more certainty, and to consider the different ways UTIs could be defined using big data.

A further strength of our study is the large sample size, which provides sufficient power to consider a wide range of clinical and sociodemographic risk factors. CIs overlapped across model specifications, suggesting that additional adjustment did not materially alter the effect estimates. This consistency across progressively adjusted models indicates that the observed temporal pattern is unlikely to be driven solely by covariate selection and supports the robustness of the findings. Analyses adjusted for the effects of significant risk factors for bladder cancer (including smoking status, BMI, common chronic conditions, and the use of hormone replacement therapy) which have not previously been reported in combination. Data on patients without bladder cancer (controls) were collected from the general CPRD population, so our estimates reflect the odds of bladder cancer associated with previous UTI frequency in a general practice context. Some patients without bladder cancer might have had other malignancies, reflecting the underlying morbidity profile of the general population, but we do not expect this issue to bias the association between recurrent UTIs and bladder cancer.

Distinguishing between the types of UTI codes is both a strength and a limitation of our study. Although we were able to categorise codes into events that are more likely to be clinical UTIs and identify those that represented repeated irritative symptoms, we could not corroborate each clinical UTI with a urine culture result due to data limitations. Our method allowed us to identify all patients with clinically significant UTIs (eg, defined by diagnosis or prescription events), who normally warrant management with antibiotics, and recurrent irritative symptoms (defined by codes for symptoms and signs) that might also be an indicator of underlying cancer symptoms. Importantly, differentiating the types of UTI codes allowed us to examine the risk associated with bladder cancer in different clinical scenarios, which currently pose challenges for primary care management.

Although our linked datasets allowed us to explore UTI episodes that were being managed in general practice, we did not have complete data on self-limiting UTIs, UTIs that were treated with antibiotics outside of our included list, and from all clinical settings that provide care for patients with UTIs, which might lead to underestimation of the absolute odds of bladder cancer in patients who were also managed in pharmacies or out-of-hours general practitioner services. A further limitation is that we did not examine the effect of haematuria. Although the presence of haematuria might weaken the association between frequency of UTIs and odds of bladder cancer, we believe the overall pattern and direction of association is unlikely to be substantially altered. As our study included patients with at least one UTI, a larger proportion of women were included in our study due to the higher incidence of UTIs in women than men. Our findings are generalisable to patients who present to the general practitioner with UTIs and representative of the risk of bladder cancer in the general population in those with at least one UTI. These results will be most applicable to high-income countries with universal health care similar to England.

We acknowledge that including an unknown category in the covariates does not fully account for the uncertainty around missing values and might still introduce bias if the missing data mechanism differs from what is implicitly assumed. As with all observational studies, residual confounding from unmeasured or incompletely measured factors cannot be excluded, despite our use of detailed covariate adjustment and matching on key demographic and clinical characteristics.

Our study showed that having recurrent UTIs was associated with an increased risk of bladder cancer at 0–24 months compared with patients who had a single episode of UTI. In particular, those presenting with recurrent UTIs in a 6-month period had the highest risk of bladder cancer, with those who had five or more UTIs showing around 2·5-times higher odds of having bladder cancer than those presenting with three UTI episodes. Our finding suggests that recurrent UTIs within short time intervals (eg, 6 months) are associated with a substantial risk of bladder cancer compared with patients who have a single episode or a repeat UTI more than 6 months from the initial episode.

We found a stronger dose–response relationship observed in female patients. This finding suggests that although female patients are more likely to have recurrent UTIs, frequent UTIs over a 6-month period should prompt general practitioners to consider further investigations. Similarly, recurrent irritative symptoms within 6 months are also associated with increased risk of bladder cancer. Clinically, enquiring about UTIs in the previous 6 months when patients present with a UTI is important and consideration of the clinical and contextual risk factors that might suggest further reviews or investigations is warranted.

A novel finding is the potential protective effect between the use of hormone replacement therapy and bladder cancer risk. Our findings suggested that patients who had recurrent UTIs and were using hormone replacement therapy (likely as recommended by NICE NG112) were at lower risk of having bladder cancer. A possible explanation is that these recurrent UTIs might have been related to postmenopausal syndrome (instead of bladder cancer), which presents similarly; therefore, enquiring about symptoms of postmenopausal syndrome and examining relevant groups of people with symptoms of recurrent UTIs is important.

The strong risk associated with recurrent UTIs within a 6-month interval was shown for all patients, irrespective of sociodemographic and clinical risk factors. Future studies should prioritise patients with repeated episodes within 6 months as the cohort of interest when studying recurrent UTIs in the context of bladder cancer, such as in the development of targeted interventions to identify groups at risk of bladder cancer and streamline their referral pathways. We propose that repeated episodes of UTIs within 6 months should be the interval of choice when defining recurrent UTIs in the context of bladder cancer. Further research is needed to quantify the risk of bladder cancer in patients with clinically significant recurrent UTIs and irritative symptoms in the general population, to determine the subgroups who would benefit from referral for specialist investigations.

Our findings identified the time interval of 6 months as the most important interval for defining recurrent UTIs in the context of bladder cancer. Defining recurrent UTIs on this basis might inform and improve consistency in future research aiming to examine bladder cancer risk and identify referral thresholds for high-risk patients with recurrent UTIs. Our findings can inform and refine existing guidelines supporting primary care referrals for suspected bladder cancer.

## Data sharing

Data are available upon request from the Clinical Practice Research Datalink (CPRD) website and subject to scientific and governance approval using the Electronic Research Applications Portal. The data dictionaries and study protocol (22_002092) are publicly available on the CPRD website.

## Declaration of interests

We declare no competing interests.
