# Supplementary material for: Defining recurrent urinary tract infections and quantifying bladder cancer risk in primary care in England: a nationwide case–control study
Source: Lancet Prim Care. 2026 Feb;2(2):None. doi: 10.1016/j.lanprc.2025.100103 (PMC12978999; doi:10.1016/j.lanprc.2025.100103)
Supplement: Supplementary appendix [file mmc1.pdf]

THE LANCET
Primary Care

Supplementary appendix

This appendix formed part of the original submission and has been peer reviewed.
We post it as supplied by the authors.

Supplement to: Mahati SG, Wu J, Walter FM, et al. Defining recurrent urinary tract infections and quantifying bladder cancer risk in primary care in England: a nationwide case–control study. *Lancet Prim Care* 2026. https://doi.org/10.1016/j.lanprc.2025.100103

Contents

Supplementary Figure 1: Number of UTIs per year per patient pre-cancer diagnosis (cases and controls) 2

Supplementary Figure 2: Diagrammatic representation of exposure variable (recurrent UTIs) 3

Supplementary Figure 3: Distributions of: a) the study population by number of UTI episodes for each year pre-diagnosis, b) people with UTI episodes over time before cancer diagnosis, and c) people with UTI episodes over time before index UTI. 4

Supplementary Figure 4: Unadjusted and adjusted odds ratios and 95% confidence intervals for bladder cancer risk by rate of UTI for over 5-year period (3-month windows in first year) 5

Supplementary Table 1: Proportions of UTI codes by type 6

Supplementary Table 2: Proportions of UTI codes by single type and combinations 7

Supplementary Table 3: Variable derivation... 8

Supplementary Table 4: Unadjusted and adjusted odds ratios and 95% confidence intervals for the association between rate of UTIs and bladder cancer 10

Supplementary Table 5: Proportion with at least 2 UTIs within 14 days 12

Supplementary Table 6: Adjusted odds ratios and 95% confidence intervals for the association between rate of UTI and bladder cancer – for 14-day grouped UTI episodes 12

Supplementary Table 7: Adjusted odds ratios and 95% confidence intervals for the association between rate of UTI and bladder cancer - stratified by sex 14

Supplementary Table 8: Bladder cancer codes 16

Supplementary Table 9: UTI diagnosis, symptom and sign codes.. 18

Supplementary Table 10: UTI prescription codes 21

Supplementary Table 11: Smoking status codes 27

Supplementary Table 12: Dementia codes.. 32

Supplementary Table 13: Learning disability codes 42

Supplementary Table 14: Stroke codes 50

Supplementary Table 15: Diabetes codes.. 55

Supplementary Table 16: Multiple sclerosis codes.. 71

Supplementary Table 17: Hormone Replacement Therapy (HRT) codes 73

Supplementary Table 18: Boby Mass Index (BMI) codes 85

Supplementary Table 19: Index of Multiple Deprivation (IMD) codes... 85

Supplementary Table 20: Sex codes 85

Supplementary Table 21: Ethnicity codes 86

Supplementary Figure 1: Number of UTIs per year per patient pre-cancer diagnosis (cases and controls)

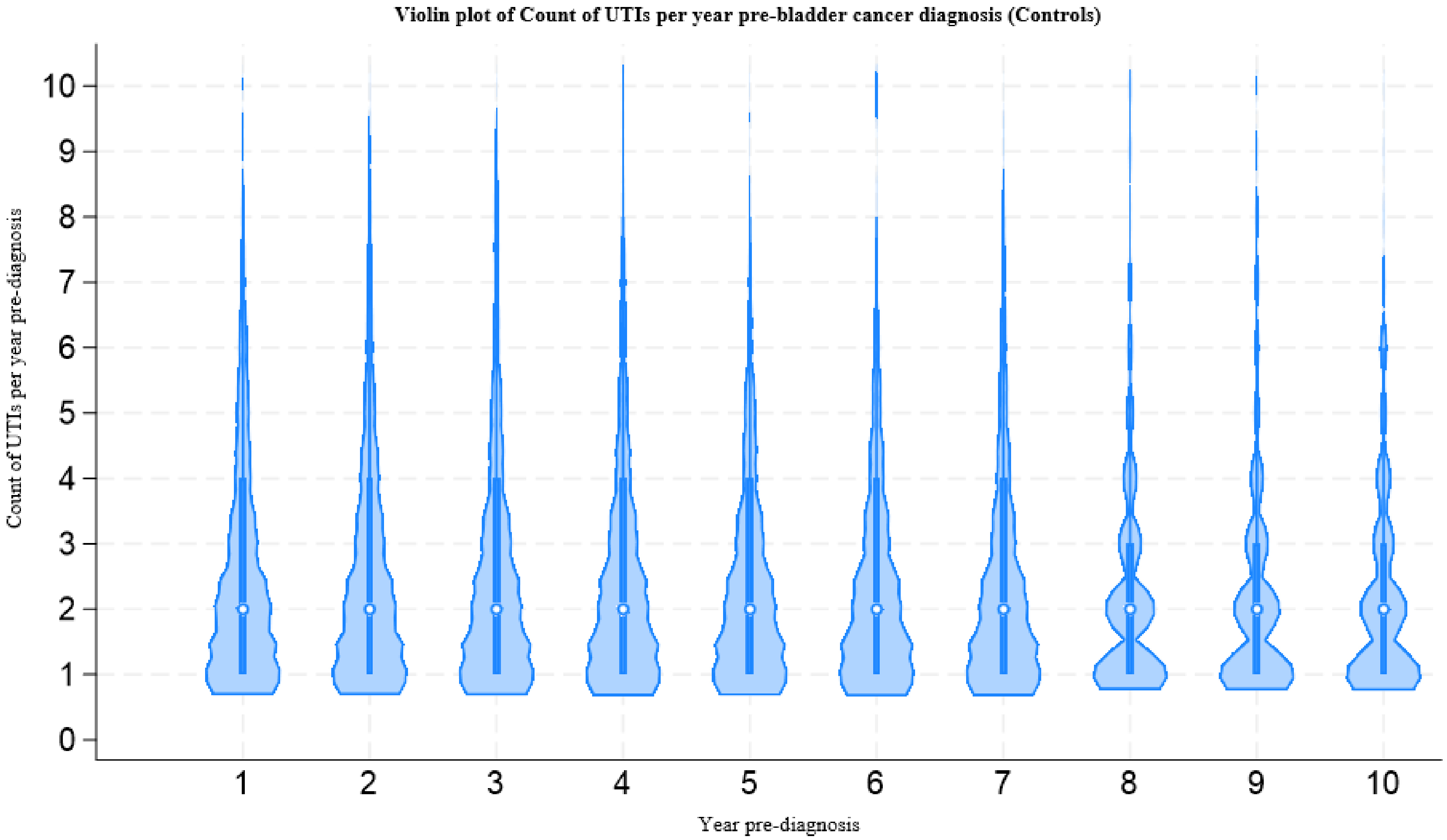

Supplementary Figure 2: Diagrammatic representation of exposure variable (recurrent UTIs)

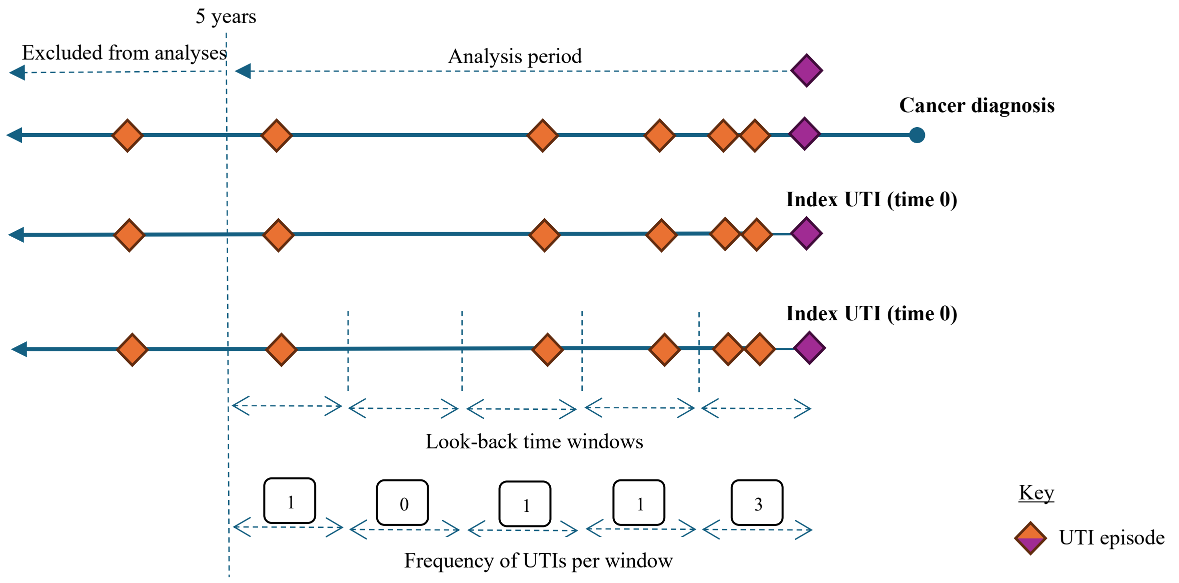

Supplementary Figure 3: Distributions of: a) the study population by number of UTI episodes for each year pre-diagnosis, b) people with UTI episodes over time before cancer diagnosis, and c) people with UTI episodes over time before index UTI.

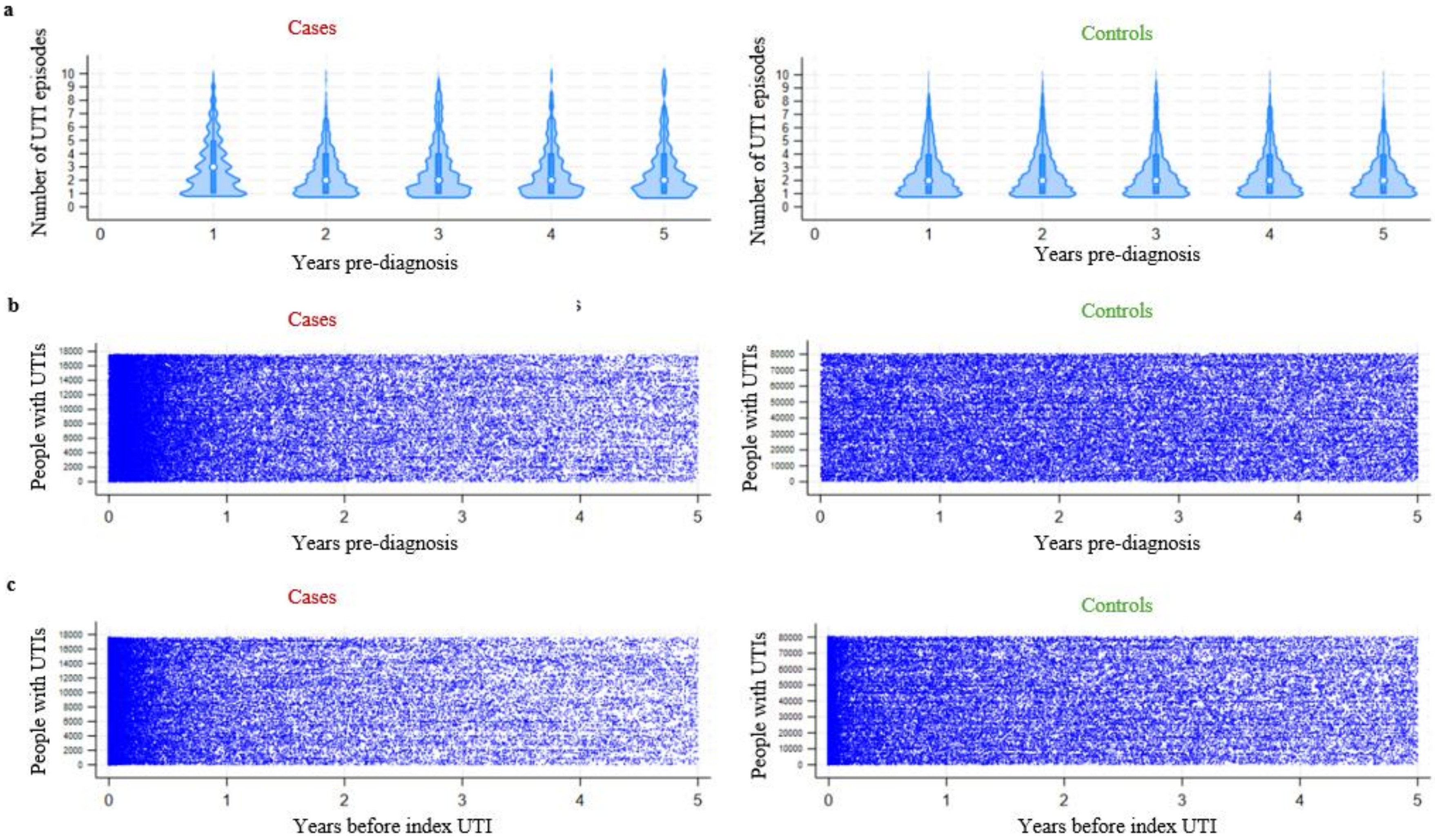

Supplementary Figure 4: Unadjusted and adjusted odds ratios and 95% confidence intervals for bladder cancer risk by rate of UTI for over 5-year period (3-month windows in first year)

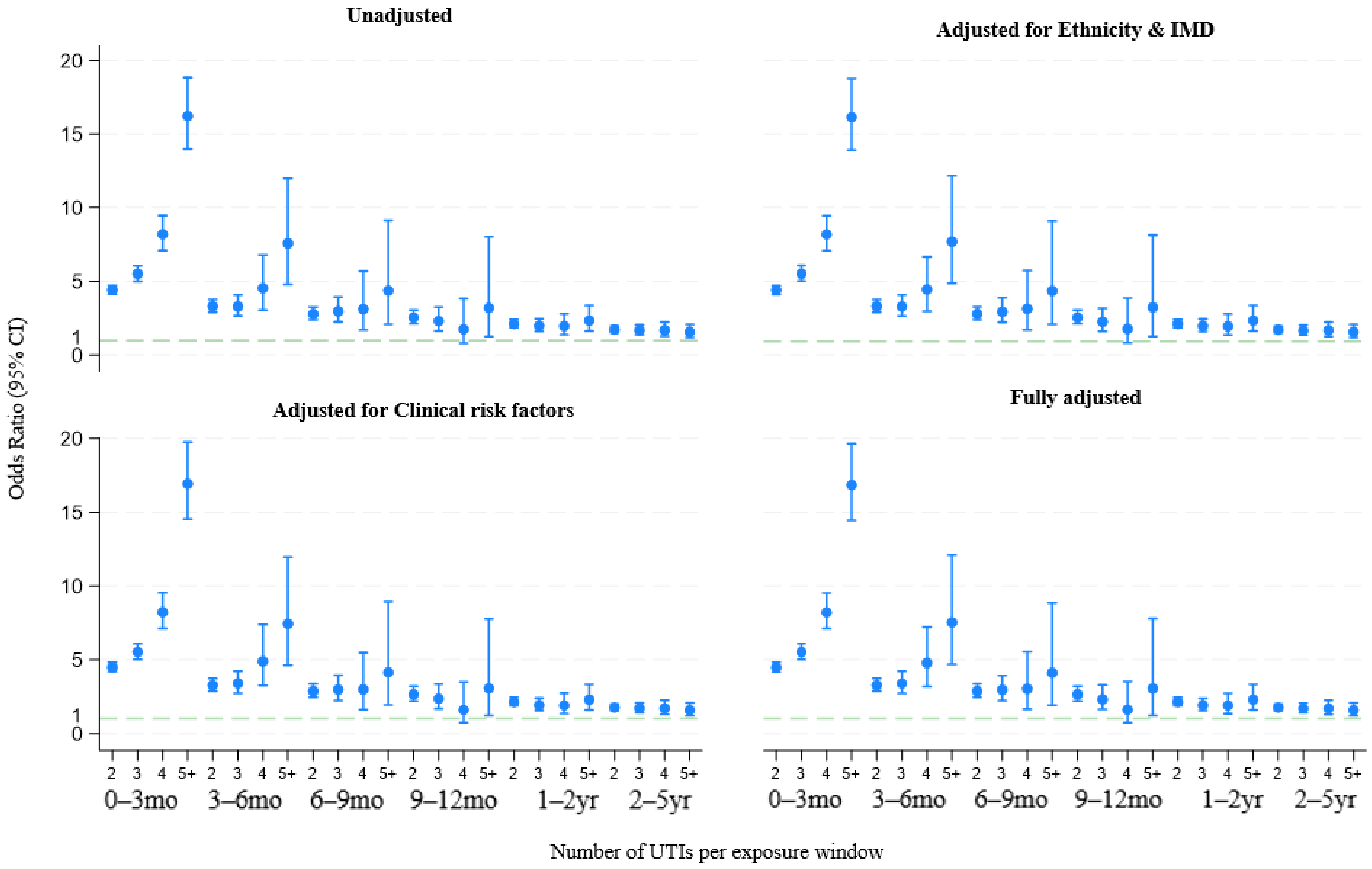

Supplementary Table 1: Proportions of UTI codes by type

Type of UTI code	Cases n (%)	Controls n (%)	Total n (%)
**Groups by single type of UTI code***			
Diagnosis	11,694 (68·1%)	24,158 (65·7%)	35,852 (66·5%)
Prescription	2,997 (17·5%)	8,338 (22·7%)	11,335 (21·0%)
Sign/Symptom	2,466 (14·4%)	4,283 (11·6%)	6,749 (12·5%)
**Combined type of UTI code****			
Diagnosis OR Prescription	14,691 (85·6%)	32,496 (88·4%)	47,187 (87·5%)
**Total**	**17,157 (100)**	**36,779 (100)**	**53,936 (100)**

*All three groups add up to 100% in columns

**each combined group as a % of the total number of cases or controls

All events grouped in the following order of priority if occurred on the same day: diagnosis>prescription>symptoms/signs

Supplementary Table 2: Proportions of UTI codes by single type and combinations

Type of UTI code	Cases<br>n (%)	Controls<br>n (%)	Total<br>n (%)
**Diagnosis only**	10,653 (62·1%)	22,436 (61·2%)	33,089 (61·5%)
**Prescription only**	2,847 (16·6%)	8,104 (22·1%)	10,951 (20·4%)
Antibiotics only^	*2,843 (99·9%)*	*8,097 (99·9%)*	*10,940 (99·9%)*
Sodium citrate only^	*0 (0·0%)*	*0 (0·0%*	*0 (0·0%*
Methenamine Hippurate^	*4 (0·1%)*	*7 (0·1%)*	*11 (0·1%)*
**Sign/Symptom only**	2,466 (14·4%)	4,283 (11·7%)	6,749 (12·5%)
**Diagnosis & Prescription***	868 (5·1%)	1,493 (4·1%)	2,361 (4·4%)
Diagnosis and antibiotics *	*868 (100%)*	*1,493 (100%)*	*2,361 (100%*
**Diagnosis & Sign/Symptom***	153 (0·9%)	194 (0·5%)	347 (0·6%)
**Prescription & Sign/Symptom***	150 (0·9%)	234 (0·6%)	384 (0·7%)
**Diagnosis & Prescription & Sign/Symptom***	20 (0·1%)	35 (0·1%)	55 (0·1%)
Total	**17,157 (100)**	**36,779 (100)**	**53,936 (100)**

*Refers to UTI events recorded on the same day

^ Percentages across rows shows the proportion of each prescription type as a percentage of all prescriptions.

Supplementary Table 3: Variable derivation

Variable Name	Derived variable	Role in Study	Data Source(s)	Period of Inclusion	Derivation Method / Notes	Management of Missing Data
**Bladder cancer diagnosis and date**	No	Outcome Timing	NCRAS Cancer Registry and CPRD Aurum / GOLD	January 1998 – December 2018	Cancer diagnosis date was taken as the first recorded instance of ICD-10 C66 or C67 in NCRAS (preferred source), or CPRD where NCRAS data was unavailable.	N/A
**Case/Control status**	No	Outcome	NCRAS Cancer Registry and CPRD Aurum / GOLD	NCRAS: January 1998 – December 2018<br><br>CPRD Aurum / GOLD: January 1988 – September 2024	**Case**: First recorded diagnosis of bladder cancer in NCRAS (ICD-10 C67 and C66) or CPRD (relevant bladder cancer medical codes).<br>**Control**: Patients with no recorded diagnosis of bladder cancer in NCRAS or CPRD. Controls were selected by CPRD from patients eligible for linkage, matched on age, sex, and practice.<br>The matched case-control dataset was provided by CPRD, based on age (±0 year), sex, and practice ID.	N/A
**Age at diagnosis**	Yes	Matching Variable	NCRAS Patient data	At bladder cancer diagnosis date	Estimated from year of birth (from CPRD) and bladder cancer diagnosis date.	No missing data
**Sex**	No	Matching Variable	NCRAS Patient data	At registration	Recorded as male and female	No missing data
**General practice ID**	No	Matching Variable	CPRD Aurum / GOLD		Used for matching cases and controls to same practice.	
**UTI events**	No	Exposure Definition	CPRD Aurum / GOLD (medical and product codes)	Up to five years before last UTI	**Definition**: Clinical diagnosis, antibiotic prescription (trimethoprim, nitrofurantoin, pivmecillinam, fosfomycin, methanamine hippurate), or UTI symptoms (dysuria, frequency, urgency, positive dipstick).<br>**Prioritisation**: If multiple codes on same day: diagnosis > prescription > symptom.	Assumed not present if not recorded
**Index UTI date**	No	Exposure Anchor	Derived from UTI events (CPRD Aurum / GOLD)	Last UTI within 5 years before cancer diagnosis (cases) or matched date (controls)	For cases: date of the last UTI prior to cancer diagnosis.<br>For controls: date of the last UTI prior to the matched case's diagnosis date.	N/A
**UTI frequency (Look-back intervals)**	Yes	Exposure	Derived from UTI events (CPRD Aurum / GOLD)	0–6 months, 6–12 months, 1–2 years, 2–5 years before last UTI	Count of UTI episodes within each pre-defined window. Categorised: 2, 3, 4, 5+ UTIs and reference being 1 UTI – which is the last UTI before diagnosis	N/A
**Ethnicity**	Yes	Covariate	Linked HES data	Available recorded Ethnicity	Ethnicity was extracted exclusively from linked HES data. Ethnic groups included: Bangladeshi, Black African, Black Caribbean, Black Other, Chinese, Indian, Mixed, Other Asian, Other, Pakistani, White and Unknown.<br><br>Patients were categorised as White, Other or Unknown, due to small proportion of patients reporting ethnicities other than White.	Missing values were classified as Unknown.

**Index of Multiple Deprivation (IMD)**	No	Covariate	Linked HES data	Available recorded IMD	IMD was extracted solely from linked HES data. CPRD did not provide IMD data in this dataset. Patients were grouped into quintiles (1 = least deprived, 5 = most deprived).	Missing IMD values were classified as Unknown.
**Smoking Status**	Yes	Covariate	CPRD Aurum / GOLD (medical or read codes)	Latest recorded before diagnosis	Categorised as non-smoker, current smoker, ex-smoker, unknown.	Missing status were Classified as Unknown.
**BMI**	No	Covariate	CPRD Aurum / GOLD (medical codes and terms)	Within 2 years of the last UTI (to account for missing values in patients who are infrequent users of primary care while maintaining clinical relevance).	BMI values were used if recorded within two years of diagnosis/index date. If direct BMI value was missing, height and weight were used to calculate BMI (kg/m^2). Grouped: underweight (<18.5), normal (18.5–24.9), overweight (25–29.9), obese (30+), unknown.	Missing values were classified as Unknown
**Comorbidities**	No	Covariate	CPRD Aurum / GOLD (medical or read codes)	Any time before last UTI	Presence of diabetes (type 1/2), dementia, learning disability, multiple sclerosis, stroke. Binary (yes/no).	Assumed not present if not recorded
**HRT Use**	No	Covariate	CPRD Aurum / GOLD (prescription codes)	Any time before last UTI	Recorded prescription for hormone replacement therapy. Binary (yes/no).	Assumed not present if not recorded

Supplementary Table 4: Unadjusted and adjusted odds ratios and 95% confidence intervals for the association between rate of UTIs and bladder cancer

Variable	Total Patients (N)	Cancer Cases n (%)	Univariate Odds ratio (95% CI)	p-value	Fully Adjusted Odds ratio (95% CI)	p-value
**1 UTI^**	32,974	7,484 (22·7%)	1·00	··	1·00	··
**0–6 Months**						
2 UTIs	6,358	3,174 (49·9%)	4·08 (3·82–4·36)	<0·001	4·11 (3·84–4·40)	<0·001
3 UTIs	3,187	1,636 (51·3%)	4·90 (4·48–5·36)	<0·001	4·95 (4·51–5·43)	<0·001
4 UTIs	1,439	838 (58·2%)	6·98 (6·13–7·94)	<0·001	7·08 (6·20–8·09)	<0·001
5+ UTIs	2,272	1,496 (65·8%)	12·71 (11·34–14·26)	<0·001	13·05 (11·60–14·68)	<0·001
**6–12 months**						
2 UTIs	1,491	586 (39·3%)	2·78 (2·45–3·15)	<0·001	2·89 (2·54–3·29)	<0·001
3 UTIs	519	186 (35·8%)	2·93 (2·37–3·62)	<0·001	2·90 (2·34–3·60)	<0·001
4 UTIs	156	55 (35·3%)	2·83 (1·92–4·18)	<0·001	2·77 (1·86–4·14)	<0·001
5+ UTIs	151	53 (35·1%)	2·85 (1·88–4·31)	<0·001	2·83 (1·85–4·33)	<0·001
**1–2 years**						
2 UTIs	1,452	505 (34·8%)	2·18 (1·91–2·48)	<0·001	2·21 (1·93–2·52)	<0·001
3 UTIs	583	182 (31·2%)	2·09 (1·69–2·59)	<0·001	2·03 (1·63–2·53)	<0·001
4 UTIs	194	57 (29·4%)	2·01 (1·41–2·88)	<0·001	1·95 (1·35–2·81)	<0·001
5+ UTIs	194	52 (26·8%)	2·48 (1·73–3·56)	<0·001	2·38 (1·64–3·45)	<0·001
**2–5 years**						
2 UTIs	1,559	482 (30·9%)	1·81 (1·59–2·06)	<0·001	1·82 (1·60–2·08)	<0·001
3 UTIs	708	186 (26·3%)	1·76 (1·45–2·14)	<0·001	1·76 (1·45–2·15)	<0·001
4 UTIs	327	97 (29·7%)	1·69 (1·28–2·23)	<0·001	1·69 (1·27–2·24)	<0·001
5+ UTIs	372	88 (23·7%)	1·67 (1·27–2·19)	<0·001	1·66 (1·25–2·20)	<0·001
**Ethnicity**						
Other	1,666	450 (14·6%)	··	··	1·00	··
White	49,874	16,028 (18·9%)	··	··	1·49 (1·27–1·74)	<0·001
Unknown	2,396	679 (8·5%)	··	··	1·16 (0·96–1·41)	0·117
**IMD quintile**						
1 – Least deprived	12,367	3,687 (16·8%)	··	··	1·00	··
2	12,202	3,926 (18·1%)	··	··	1·09 (1·01–1·18)	0·02
3	10,857	3,389 (17·5%)	··	··	1·03 (0·96–1·12)	0·398
4	9,845	3,188 (18·5%)	··	··	1·08 (0·99–1·17)	0·089
5 – Most deprived	8,611	2,953 (19·5%)	··	··	1·21 (1·10–1·33)	<0·001
Unknown	54	14 (6·9%)	··	··	0·90 (0·42–1·95)	0·798
**BMI group**						
Normal (18.5-24.9)	11,696	3,636 (18·3%)	··	··	1·00	··
Underweight (<18.5)	926	400 (28·4%)	··	··	1·63 (1·37–1·93)	<0·001
Overweight (25.0-29.9)	14,052	4,137 (16·8%)	··	··	0·81 (0·76–0·87)	<0·001
Obese (30+)	9,468	2,831 (17·8%)	··	··	0·79 (0·73–0·85)	<0·001
Unknown	17,794	6,153 (18·2%)	··	··	1·19 (1·12–1·28)	<0·001

Smoking status						
Non-smoker	24,217	6,102 (15·5%)	··	··	1·00	··
Current smoker	13,552	5,817 (26·3%)	··	··	2·14 (2·01–2·27)	<0·001
Ex-smoker	12,570	4,236 (20·6%)	··	··	1·50 (1·41–1·59)	<0·001
Unknown	3,597	1,002 (7·4%)	··	··	1·07 (0·96–1·19)	0·227
**Diabetes**						
No	45,953	14,278 (17·2%)	··	··	1·00	··
Yes	7,983	2,879 (23·2%)	··	··	1·38 (1·29–1·47)	<0·001
**Neurological conditions***						
No	49,518	15,651 (17·6%)	··	··	1·00	··
Yes	4,418	1,506 (23·4%)	··	··	1·13 (1·04–1·23)	0·003
**HRT use**						
No	45,117	14,906 (17·8%)	··	··	1·00	··
Yes	8,819	2,251 (18·8%)	··	··	0·77 (0·71–0·83)	<0·001

^ Reference group for the exposure time windows

** Neurological conditions include: dementia, learning disability, multiple sclerosis, stroke*

† BMI – body mass index; HRT – hormone replacement therapy; IMD – Index of Multiple Deprivation

Supplementary Table 5: Proportion with at least 2 UTIs within 14 days

Had at least 2 UTIs within 14 days	Cases	Controls	Total
Yes	5,204 (30.3%)	8,322 (15.7%)	13,526 (20.4%)
No	11,953 (69.7%)	28,457 (84.3%)	40,410 (79.6%)
**Total**	**17,157 (100%)**	**36,779 (100%)**	**53,936 (100%)**

Supplementary Table 6: Adjusted odds ratios and 95% confidence intervals for the association between rate of UTI and bladder cancer – for 14-day grouped UTI episodes

	Main analysis			14-Day Grouped Analysis		
**Variable**	**Total Patients (N)**	**Cancer Cases n (%)**	**Odds ratio (95% CI)**	**Total Patients (N)**	**Cancer Cases n (%)**	**Odds ratio (95% CI)**
**1 UTI^**	32,974	7,484 (22·7%)	1·00	35,252	8,666 (24·6%)	1·00
**0–6 Months**						
2 UTIs	6,358	3,174 (49·9%)	4·11 (3·84–4·40) **	6,582	3,449 (52·4%)	4·20 (3·93–4·49) **
3 UTIs	3,187	1,636 (51·3%)	4·95 (4·51–5·43) **	1,969	1,185 (60·2%)	6·49 (5·80–7·25) **
4 UTIs	1,439	838 (58·2%)	7·08 (6·20–8·09) **	767	501 (65·3%)	8·69 (7·28–10·37) **
5+ UTIs	2,272	1,496 (65·8%)	13·05 (11·60–14·68) **	443	296 (66·8%)	12·06 (9·44–15·40) **
**6–12 months**						
2 UTIs	1,491	586 (39·3%)	2·89 (2·54–3·29) **	2,207	849 (38·5%)	2·63 (2·36–2·92) **
3 UTIs	519	186 (35·8%)	2·90 (2·34–3·60) **	384	155 (40·4%)	2·83 (2·21–3·61) **
4 UTIs	156	55 (35·3%)	2·77 (1·86–4·14) **	84	31 (36·9%)	2·42 (1·41–4·15) **
5+ UTIs	151	53 (35·1%)	2·83 (1·85–4·33) **	35	18 (51·4%)	4·44 (2·05–9·64) **
**1–2 years**						
2 UTIs	1,452	505 (34·8%)	2·21 (1·93–2·52) **	2,132	721 (33·8%)	2·05 (1·84–2·30) **
3 UTIs	583	182 (31·2%)	2·03 (1·63–2·53) **	468	165 (35·3%)	2·12 (1·68–2·67) **
4 UTIs	194	57 (29·4%)	1·95 (1·35–2·81) **	135	54 (40·0%)	2·57 (1·72–3·83) **
5+ UTIs	194	52 (26·8%)	2·38 (1·64–3·45) **	64	24 (37·5%)	2·53 (1·42–4·50) **
**2–5 years**						
2 UTIs	1,559	482 (30·9%)	1·82 (1·60–2·08) **	2,247	696 (31·0%)	1·74 (1·56–1·94) **
3 UTIs	708	186 (26·3%)	1·76 (1·45–2·15) **	677	194 (28·7%)	1·64 (1·34–2·00) **
4 UTIs	327	97 (29·7%)	1·69 (1·27–2·24) **	271	86 (31·7%)	2·00 (1·49–2·69) **
5+ UTIs	372	88 (23·7%)	1·66 (1·25–2·20) **	219	67 (30·6%)	2·02 (1·45–2·82) **
**Ethnicity**						
Other	1,666	450 (27·0%)	1·00	1,666	450 (27·0%)	1·00

White	49,874	16,028 (32·1%)	1·49 (1·27–1·74) **	49,874	16,028 (32·1%)	1·49 (1·28–1·74) **
Unknown	2,396	679 (8·5%)	1·16 (0·96–1·41)	2,396	679 (28·3%)	1·18 (0·98–1·42)
**IMD quintile**						
1 – Least deprived	12,367	3,687 (16·8%)	1·00	12,367	3,687 (29·8%)	1·00
2	12,202	3,926 (18·1%)	1·09 (1·01–1·18)	12,202	3,926 (32·2%)	1·11 (1·03–1·19)
3	10,857	3,389 (17·5%)	1·03 (0·96–1·12)	10,857	3,389 (31·2%)	1·05 (0·97–1·13)
4	9,845	3,188 (18·5%)	1·08 (0·99–1·17)	9,845	3,188 (32·4%)	1·10 (1·01–1·19)
5 – Most deprived	8,611	2,953 (19·5%)	1·21 (1·10–1·33)	8,611	2,953 (34·3%)	1·21 (1·11–1·33) **
Unknown	54	14 (6·9%)	0·90 (0·42–1·95)	54	14 (25·9%)	0·85 (0·41–1·78)
**BMI group**						
Normal (18.5-24.9)	11,696	3,636 (18·3%)	1·00	11,696	3,636 (31·1%)	1·00
Underweight (<18.5)	926	400 (28·4%)	1·63 (1·37–1·93)	926	400 (43·2%)	1·66 (1·40–1·95) **
Overweight (25.0-29.9)	14,052	4,137 (16·8%)	0·81 (0·76–0·87)	14,052	4,137 (29·4%)	0·82 (0·76–0·87) **
Obese (30+)	9,468	2,831 (17·8%)	0·79 (0·73–0·85)	9,468	2,831 (29·9%)	0·79 (0·73–0·85) **
Unknown	17,794	6,153 (18·2%)	1·19 (1·12–1·28)	17,794	6,153 (34·6%)	1·21 (1·13–1·29) **
**Smoking status**						
Non-smoker	24,217	6,102 (15·5%)	1·00	24,217	6,102 (25·2%)	1·00
Current smoker	13,552	5,817 (26·3%)	2·14 (2·01–2·27)	13,552	5,817 (42·9%)	2·14 (2·02–2·27) **
Ex-smoker	12,570	4,236 (20·6%)	1·50 (1·41–1·59)	12,570	4,236 (33·7%)	1·49 (1·40–1·58) **
Unknown	3,597	1,002 (7·4%)	1·07 (0·96–1·19)	3,597	1,002 (27·9%)	1·06 (0·95–1·17)
**Diabetes**						
No	45,953	14,278 (17·2%)	1·00	45,953	14,278 (31·1%)	1·00
Yes	7,983	2,879 (23·2%)	1·38 (1·29–1·47)	7,983	2,879 (36·1%)	1·36 (1·28–1·45) **
**Neurological conditions***						
No	49,518	15,651 (17·6%)	1·00	49,518	15,651 (31·6%)	1·00
Yes	4,418	1,506 (23·4%)	1·13 (1·04–1·23)	4,418	1,506 (34·1%)	1·12 (1·04–1·22) **
**HRT use**						
No	45,117	14,906 (17·8%)	1·00	45,117	14,906 (33·0%)	1·00
Yes	8,819	2,251 (18·8%)	0·77 (0·71–0·83)	8,819	2,251 (25·5%)	0·77 (0·72–0·84) **

^ Reference group for the exposure time windows

** Neurological conditions include: dementia, learning disability, multiple sclerosis, stroke*

† BMI – body mass index; HRT – hormone replacement therapy; IMD – Index of Multiple Deprivation

*** Statistically significant at the 95% confidence interval level*

Supplementary Table 7: Adjusted odds ratios and 95% confidence intervals for the association between rate of UTI and bladder cancer - stratified by sex

	Male			Female		
**Variable**	**Total Patients (N)**	**Cancer Cases n (%)**	**Odds ratio (95% CI)**	**Total Patients (N)**	**Cancer Cases n (%)**	**Odds ratio (95% CI)**
**1 UTI^**	17,818	5 051 (28·4%)	1·00	15,156	2,433 (16·1%)	1·00
**0–6 Months**						
2 UTIs	3,077	1 796 (58·4%)	4·01 (3·64–4·43) **	3,281	1 378 (42·0%)	4·30 (3·90–4·74) **
3 UTIs	1,522	857 (56·3%)	4·18 (3·65–4·79) **	1,665	779 (46·8%)	5·76 (5·08–6·54) **
4 UTIs	684	413 (60·4%)	5·20 (4·28–6·32) **	755	425 (56·3%)	9·29 (7·75–11·14) **
5+ UTIs	958	580 (60·5%)	6·54 (5·48–7·81) **	1,314	916 (69·7%)	20·74 (17·67–24·34) **
**6–12 months**						
2 UTIs	573	299 (52·2%)	2·96 (2·42–3·62) **	918	287 (31·3%)	2·96 (2·50–3·50) **
3 UTIs	204	94 (46·1%)	2·88 (2·06–4·04) **	315	92 (29·2%)	3·01 (2·26–4·00) **
4 UTIs	58	23 (39·7%)	1·77 (0·88–3·57)	98	32 (32·7%)	3·57 (2·19–5·80) **
5+ UTIs	64	30 (46·9%)	3·52 (1·90–6·52) **	87	23 (26·4%)	2·25 (1·23–4·11) **
**1–2 years**						
2 UTIs	540	265 (49·1%)	2·50 (2·03–3·08) **	912	240 (26·3%)	2·11 (1·77–2·52) **
3 UTIs	202	88 (43·6%)	2·16 (1·50–3·11) **	381	94 (24·7%)	2·02 (1·53–2·66) **
4 UTIs	63	27 (42·9%)	2·69 (1·49–4·88) **	131	30 (22·9%)	1·75 (1·08–2·83) **
5+ UTIs	78	23 (29·5%)	1·92 (1·07–3·45) **	116	29 (25·0%)	2·95 (1·82–4·78) **
**2–5 years**						
2 UTIs	576	256 (44·4%)	2·14 (1·74–2·63) **	983	226 (23·0%)	1·68 (1·41–2·01) **
3 UTIs	249	90 (36·1%)	1·72 (1·25–2·36) **	459	96 (20·9%)	1·85 (1·43–2·39) **
4 UTIs	119	50 (42·0%)	2·05 (1·34–3·15) **	208	47 (22·6%)	1·50 (1·01–2·22) **
5+ UTIs	128	50 (39·1%)	2·19 (1·43–3·36) **	244	38 (15·6%)	1·39 (0·94–2·06)
**Ethnicity**						
Other	923	296 (32·1%)	1·00	743	154 (20·7%)	1·00
White	25,003	9,370 (37·5%)	1·54 (1·26–1·89) **	24,871	6,658 (26·8%)	1·41 (1·11–1·79) **
Unknown	987	326 (33·0%)	1·13 (0·87–1·47)	1,409	353 (25·1%)	1·16 (0·87–1·54)
**IMD quintile**						
1 – Least deprived	6,209	2,184 (35·2%)	1·00	6,158	1,503 (24·4%)	1·00
2	6,234	2,314 (37·1%)	1·06 (0·96–1·18)	5,968	1,612 (27·0%)	1·12 (1·01–1·25) **
3	5,495	1,990 (36·2%)	1·02 (0·92–1·14)	5,362	1,399 (26·1%)	1·06 (0·94–1·19)
4	4,856	1,848 (38·1%)	1·07 (0·95–1·20)	4,989	1,340 (26·9%)	1·09 (0·96–1·23)
5 – Most deprived	4,091	1,648 (40·3%)	1·21 (1·06–1·38) **	4,520	1,305 (28·9%)	1·21 (1·05–1·39) **
Unknown	28	8 (28·6%)	0·70 (0·24–2·05)	26	6 (23·1%)	1·23 (0·44–3·44)
**BMI group**						
Normal (18.5-24.9)	5,617	2,107 (37·5%)	1·00	6,079	1,529 (25·2%)	1·00
Underweight (<18.5)	301	160 (53·2%)	1·74 (1·30–2·33) **	625	240 (38·4%)	1·61 (1·30–2·00) **
Overweight (25.0-29.9)	8,124	2,787 (34·3%)	0·79 (0·73–0·87) **	5,928	1,350 (22·8%)	0·81 (0·73–0·90) **
Obese (30+)	4,664	1,641 (35·2%)	0·72 (0·65–0·80) **	4,804	1,190 (24·8%)	0·87 (0·78–0·97) **

Unknown	8,207	3,297 (40·2%)	1·08 (0·98–1·19)	9,587	2,856 (29·8%)	1·31 (1·19–1·45) **
**Smoking status**						
Non-smoker	10,036	3,013 (30·0%)	1·00	14,181	3,089 (21·8%)	1·00
Current smoker	7,369	3,587 (48·7%)	2·06 (1·90–2·24) **	6,183	2,230 (36·1%)	2·20 (2·02–2·39) **
Ex-smoker	7,870	2,894 (36·8%)	1·43 (1·31–1·55) **	4,700	1,342 (28·6%)	1·56 (1·42–1·71) **
Unknown	1,638	498 (30·4%)	0·87 (0·75–1·02)	1,959	504 (25·7%)	1·28 (1·10–1·48) **
**Diabetes**						
No	22,201	8,105 (36·5%)	1·00	23,752	6,173 (26·0%)	1·00
Yes	4,712	1 887 (40·1%)	1·36 (1·25–1·49) **	3,271	992 (30·3%)	1·38 (1·24–1·53) **
**Neurological conditions***						
No	24,616	9,105 (37·0%)	1·00	24,902	6,546 (26·3%)	1·00
Yes	2,297	887 (38·6%)	1·14 (1·01–1·27) **	2,121	619 (29·2%)	1·13 (1·00–1·28)
**HRT use**						
No	26,903	9,987 (37·1%)	1·00	18,214	4,919 (27·0%)	1·00
Yes	10	5 (50·0%)	4·17 (0·97–17·92)	8,809	2,246 (25·5%)	0·76 (0·70–0·82) **

^ Reference group for the exposure time windows

** Neurological conditions include: dementia, learning disability, multiple sclerosis, stroke*

† BMI – body mass index; HRT – hormone replacement therapy; IMD – Index of Multiple Deprivation

*** Statistically significant at the 95% confidence interval level*

Supplementary Table 8: Bladder cancer codes

ICD-10 code		Description	Database
C66		Malignant neoplasm of Ureter	NCRAS
C67		Malignant neoplasm of Bladder	NCRAS
**Medcode ID**	**Snomed-CT ID**	**Term**	
406263015	271468000	Malignant neoplasm of genitourinary organ	CPRD Aurum
380092019	255066001	Carcinoma of genitourinary organ	CPRD Aurum
1786811017	399326009	Malignant neoplasm of urinary bladder	CPRD Aurum
289262014	188239000	Malignant neoplasm of trigone of urinary bladder	CPRD Aurum
289267015	188240003	Malignant neoplasm of dome of urinary bladder	CPRD Aurum
289270016	188241004	Malignant neoplasm of lateral wall of urinary bladder	CPRD Aurum
289271017	188242006	Malignant neoplasm of anterior wall of urinary bladder	CPRD Aurum
289272012	188243001	Malignant neoplasm of posterior wall of urinary bladder	CPRD Aurum
289275014	188244007	Malignant neoplasm of bladder neck	CPRD Aurum
289276010	188245008	Malignant neoplasm of ureteric orifice	CPRD Aurum
720951000006119	363456000	Malignant neoplasm of urachus	CPRD Aurum
289279015	399326009	Malignant neoplasm of other site of urinary bladder	CPRD Aurum
289280017	188247000	Malignant neoplasm, overlapping lesion of bladder	CPRD Aurum
289281018	399326009	Malignant neoplasm of urinary bladder NOS	CPRD Aurum
729481000006112	419052002	Malignant tumour of urinary system	CPRD Aurum
720961000006117	363458004	Malignant tumour of ureter	CPRD Aurum
720991000006113	363459007	Malignant neoplasm of urethra	CPRD Aurum
289297011	419052002	Malignant neoplasm of other urinary organs	CPRD Aurum
289298018	188256008	Malignant neoplasm of overlapping lesion of urinary organs	CPRD Aurum
289299014	419052002	Malignant tumour of urinary system	CPRD Aurum
725761000006111	271468000	Malignant neoplasm of genitourinary organ OS	CPRD Aurum
289301019	271468000	Malignant neoplasm of genitourinary organ NOS	CPRD Aurum
290867017	109355002	Carcinoma in situ of breast and genitourinary system	CPRD Aurum
153107012	92546004	Carcinoma in situ of bladder	CPRD Aurum
290894018	255150000	Carcinoma in situ of urinary organs NOS	CPRD Aurum
511442015	94754000	Neoplasm of uncertain behaviour of bladder	CPRD Aurum
291300019	126885006	Neoplasm of bladder	CPRD Aurum
317731000006116	118287003	Transitional cell papilloma AND/OR carcinoma	CPRD Aurum
317671000006111	53530009	Transitional cell carcinoma in situ	CPRD Aurum
291363015	27090000	Transitional cell carcinoma	CPRD Aurum
318001000006113	27090000	Urothelial carcinoma	CPRD Aurum
317691000006112	112676006	[M]Transitional cell carcinoma, spindle cell type	CPRD Aurum
315031000006113	12400006	Papillary transitional cell carcinoma	CPRD Aurum
291364014	118287003	[M]Transitional cell papilloma or carcinoma NOS	CPRD Aurum
292147019	419052002	[X]Malignant neoplasm of urinary tract	CPRD Aurum
292148012	448233000	Malignant neoplasm of urinary organ	CPRD Aurum
1227573010	415087005	[V]Personal history of malignant neoplasm of bladder	CPRD Aurum
	**Readcode**	**Readterm**	
	B4...00	Malignant neoplasm of genitourinary organ	CPRD GOLD
	B4...11	Carcinoma of genitourinary organ	CPRD GOLD
	B49..00	Malignant neoplasm of urinary bladder	CPRD GOLD

	B490.00	Malignant neoplasm of trigone of urinary bladder	CPRD GOLD
	B491.00	Malignant neoplasm of dome of urinary bladder	CPRD GOLD
	B492.00	Malignant neoplasm of lateral wall of urinary bladder	CPRD GOLD
	B493.00	Malignant neoplasm of anterior wall of urinary bladder	CPRD GOLD
	B494.00	Malignant neoplasm of posterior wall of urinary bladder	CPRD GOLD
	B495.00	Malignant neoplasm of bladder neck	CPRD GOLD
	B496.00	Malignant neoplasm of ureteric orifice	CPRD GOLD
	B497.00	Malignant neoplasm of urachus	CPRD GOLD
	B49y.00	Malignant neoplasm of other site of urinary bladder	CPRD GOLD
	B49y000	Malignant neoplasm; overlapping lesion of bladder	CPRD GOLD
	B49z.00	Malignant neoplasm of urinary bladder NOS	CPRD GOLD
	B4A2.00	Malignant neoplasm of ureter	CPRD GOLD
	B4A3.00	Malignant neoplasm of urethra	CPRD GOLD
	B4Ay.00	Malignant neoplasm of other urinary organs	CPRD GOLD
	B4Ay000	Malignant neoplasm of overlapping lesion of urinary organs	CPRD GOLD
	B4y..00	Malignant neoplasm of genitourinary organ OS	CPRD GOLD
	B4z..00	Malignant neoplasm of genitourinary organ NOS	CPRD GOLD
	B83..00	Carcinoma in situ of breast and genitourinary system	CPRD GOLD
	B837.00	Carcinoma in situ of bladder	CPRD GOLD
	B83z.00	Carcinoma in situ of urinary organs NOS	CPRD GOLD
	B917.00	Neoplasm of uncertain behaviour of bladder	CPRD GOLD
	BA04.00	Neoplasm of unspecified nature of bladder	CPRD GOLD
	BB4..00	[M]Transitional cell papillomas and carcinomas	CPRD GOLD
	BB42.00	[M]Transitional cell carcinoma in situ	CPRD GOLD
	BB43.00	[M]Transitional cell carcinoma NOS	CPRD GOLD
	BB43.11	[M]Urothelial carcinoma	CPRD GOLD
	BB47.00	[M]Transitional cell carcinoma, spindle cell type	CPRD GOLD
	BB4A.00	[M]Papillary transitional cell carcinoma	CPRD GOLD
	BB4z.00	[M]Transitional cell papilloma or carcinoma NOS	CPRD GOLD
	Byu9.00	[X]Malignant neoplasm of urinary tract	CPRD GOLD
	Byu9000	[X]Malignant neoplasm of urinary organ; unspecified	CPRD GOLD
	ZV10511	[V]Personal history of malignant neoplasm of bladder	CPRD GOLD

Supplementary Table 9: UTI diagnosis, symptom and sign codes

Medcode ID	Snomed CT ID	Term	Type of code	Database
304323018	68566005	Urinary tract infection, site not specified NOS	Diagnosis	CPRD Aurum
65119018	38822007	Cystitis	Diagnosis	CPRD Aurum
74781000006117	68566005	Urinary tract infection, site not specified	Diagnosis	CPRD Aurum
304205011	197853008	Recurrent cystitis	Diagnosis	CPRD Aurum
183141000006112	197927001	Recurrent urinary tract infection	Diagnosis	CPRD Aurum
304084019	45816000	Pyelonephritis	Diagnosis	CPRD Aurum
61206018	36689008	Acute pyelonephritis	Diagnosis	CPRD Aurum
411392011	275412000	Cystitis of pregnancy	Diagnosis	CPRD Aurum
304321016	197928006	Chronic urinary tract infection	Diagnosis	CPRD Aurum
39884018	23754003	Calculous pyelonephritis	Diagnosis	CPRD Aurum
183171000006116	197927001	Recurrent urinary tract infection	Diagnosis	CPRD Aurum
304186019	33655002	Chronic cystitis	Diagnosis	CPRD Aurum
459357017	314940005	Suspected UTI (urinary tract infection)	Diagnosis	CPRD Aurum
183161000006111	197927001	Recurrent urinary tract infections	Diagnosis	CPRD Aurum
41678019	24868007	Acute gonococcal cystitis	Diagnosis	CPRD Aurum
304319014	197927001	Recurrent urinary tract infection	Diagnosis	CPRD Aurum
304206012	38822007	Other specified cystitis	Diagnosis	CPRD Aurum
304187011	33655002	Other chronic cystitis NOS	Diagnosis	CPRD Aurum
304209017	38822007	Cystitis NOS	Diagnosis	CPRD Aurum
216611000006114	197926005	Postoperative urinary tract infection	Diagnosis	CPRD Aurum
450823012	307534009	UTI - urinary tract infection in pregnancy	Diagnosis	CPRD Aurum
113331011	68226007	Acute cystitis	Diagnosis	CPRD Aurum
304367016	128606002	Urethral and urinary tract disorders NOS	Diagnosis	CPRD Aurum
22733019	13285005	Cystitis cystica	Diagnosis	CPRD Aurum
112408019	67685000	Prostatocystitis	Diagnosis	CPRD Aurum
8285011	4324001	Subacute cystitis	Diagnosis	CPRD Aurum
304184016	33655002	Other chronic cystitis	Diagnosis	CPRD Aurum
304208013	38822007	Other cystitis NOS	Diagnosis	CPRD Aurum
65241012	38898003	Xanthogranulomatous pyelonephritis	Diagnosis	CPRD Aurum
304071011	36689008	Acute pyelonephritis NOS	Diagnosis	CPRD Aurum
147258010	88813005	Chronic gonococcal cystitis	Diagnosis	CPRD Aurum
108231015	44323002	Tuberculous pyelonephritis	Diagnosis	CPRD Aurum
304083013	129128006	Pyelonephritis and pyonephrosis unspecified	Diagnosis	CPRD Aurum
304090015	45816000	Unspecified pyelonephritis NOS	Diagnosis	CPRD Aurum
304068015	197768004	Acute pyelonephritis without medullary necrosis	Diagnosis	CPRD Aurum
305203014	38822007	[X]Other cystitis	Diagnosis	CPRD Aurum
305202016	33655002	[X]Other chronic cystitis	Diagnosis	CPRD Aurum
304087014	197782004	Pyelonephritis in diseases EC	Diagnosis	CPRD Aurum
113884018	68566005	Urinary tract infection	Diagnosis	CPRD Aurum
259772017	364688007	Urine smell	Sign	CPRD Aurum
259777011	364688007	Urine smell NOS	Sign	CPRD Aurum
70181000006112	8769003	Urine smell abnormal	Sign	CPRD Aurum
259775015	167249005	Urine smell fishy	Sign	CPRD Aurum
259774016	167248002	Urine smell ammoniacal	Sign	CPRD Aurum

82701015	49650001	Dysuria	Symptom	CPRD Aurum
317512015	49650001	[D]Painful urination	Symptom	CPRD Aurum
317511010	49650001	[D]Dysuria	Symptom	CPRD Aurum
412649012	276498006	C/O - ureteric pain	Symptom	CPRD Aurum
11878012	6561007	Urethral pain	Symptom	CPRD Aurum
317514019	49650001	[D]Dysuria NOS	Symptom	CPRD Aurum
318021013	49650001	Painful micturition	Symptom	CPRD Aurum
1494943010	231517009	[X]Psychogenic dysuria	Symptom	CPRD Aurum
295388019	191978003	Psychogenic dysuria	Symptom	CPRD Aurum
124716012	75088002	Urgency of micturition	Symptom	CPRD Aurum
47612016	28442001	Polyuria	Symptom	CPRD Aurum
252595010	162053006	Suprapubic pain	Symptom	CPRD Aurum
292171000006114	75088002	[D] Urgency of micturition	Symptom	CPRD Aurum
317558019	6744007	[D]Vesical tenesmus	Symptom	CPRD Aurum
317532019	28442001	[D]Polyuria	Symptom	CPRD Aurum
317571016	162053006	[D]Suprapubic pain	Symptom	CPRD Aurum
317528013	274734008	Micturition frequency and polyuria	Symptom	CPRD Aurum
215531000006114	28442001	Polyuria	Symptom	CPRD Aurum
317534018	274734008	[D]Frequency of micturition or polyuria NOS	Symptom	CPRD Aurum
**Medcode**		**term**	**typeofcode**	**database**
150		Urinary tract infection, site not specified NOS	Diagnosis	CPRD GOLD
389		Cystitis	Diagnosis	CPRD GOLD
1289		Urinary tract infection, site not specified	Diagnosis	CPRD GOLD
1353		Recurrent cystitis	Diagnosis	CPRD GOLD
1572		Recurrent urinary tract infection	Diagnosis	CPRD GOLD
1899		Pyelonephritis unspecified	Diagnosis	CPRD GOLD
2546		Acute pyelonephritis	Diagnosis	CPRD GOLD
2602		Cystitis of pregnancy	Diagnosis	CPRD GOLD
2650		Chronic urinary tract infection	Diagnosis	CPRD GOLD
2939		Calculous pyelonephritis	Diagnosis	CPRD GOLD
2985		Recurrent UTI	Diagnosis	CPRD GOLD
3469		Chronic cystitis unspecified	Diagnosis	CPRD GOLD
7579		Suspected UTI	Diagnosis	CPRD GOLD
9378		Recurrent urinary tract infections	Diagnosis	CPRD GOLD
10295		Acute gonococcal cystitis	Diagnosis	CPRD GOLD
10515		Recurrent urinary tract infection	Diagnosis	CPRD GOLD
10857		Other specified cystitis	Diagnosis	CPRD GOLD
11315		Other chronic cystitis NOS	Diagnosis	CPRD GOLD
12484		Cystitis NOS	Diagnosis	CPRD GOLD
12570		Post operative urinary tract infection	Diagnosis	CPRD GOLD
14644		UTI - urinary tract infection in pregnancy	Diagnosis	CPRD GOLD
15074		Acute cystitis	Diagnosis	CPRD GOLD
15787		Urethral and urinary tract disorders NOS	Diagnosis	CPRD GOLD
22682		Cystitis cystica	Diagnosis	CPRD GOLD
29497		Prostatocystitis	Diagnosis	CPRD GOLD
30068		Subacute cystitis	Diagnosis	CPRD GOLD

32787		Other chronic cystitis	Diagnosis	CPRD GOLD
34630		Other cystitis NOS	Diagnosis	CPRD GOLD
38572		Xanthogranulomatous pyelonephritis	Diagnosis	CPRD GOLD
38698		Acute pyelonephritis NOS	Diagnosis	CPRD GOLD
48908		Chronic gonococcal cystitis	Diagnosis	CPRD GOLD
49235		Tuberculous pyelonephritis	Diagnosis	CPRD GOLD
53944		Pyelonephritis and pyonephrosis unspecified	Diagnosis	CPRD GOLD
59121		Unspecified pyelonephritis NOS	Diagnosis	CPRD GOLD
64482		Acute pyelonephritis without medullary necrosis	Diagnosis	CPRD GOLD
70189		[X]Other cystitis	Diagnosis	CPRD GOLD
72686		[X]Other chronic cystitis	Diagnosis	CPRD GOLD
95710		Pyelonephritis in diseases EC	Diagnosis	CPRD GOLD
97002		Urinary tract infection	Diagnosis	CPRD GOLD
8904		Urine smell	Sign	CPRD GOLD
19983		Urine smell NOS	Sign	CPRD GOLD
23841		Urine smell abormal	Sign	CPRD GOLD
27002		Urine smell fishy	Sign	CPRD GOLD
32031		Urine smell ammoniacal	Sign	CPRD GOLD
532		Dysuria	Symptom	CPRD GOLD
4498		[D]Painful urination	Symptom	CPRD GOLD
5350		[D]Dysuria	Symptom	CPRD GOLD
7014		C/O - ureteric pain	Symptom	CPRD GOLD
8433		Urethral pain	Symptom	CPRD GOLD
15599		[D]Dysuria NOS	Symptom	CPRD GOLD
52859		[X]Painful micturition, unspecified	Symptom	CPRD GOLD
53122		[X]Psychogenic dysuria	Symptom	CPRD GOLD
55781		Psychogenic dysuria	Symptom	CPRD GOLD
583		Urgency of micturition	Symptom	CPRD GOLD
4103		Polyuria	Symptom	CPRD GOLD
7300		Suprapubic pain	Symptom	CPRD GOLD
8028		[D] Urgency of micturition	Symptom	CPRD GOLD
15389		[D]Vesical tenesmus	Symptom	CPRD GOLD
16399		[D]Polyuria	Symptom	CPRD GOLD
20475		[D]Suprapubic pain	Symptom	CPRD GOLD
23662		[D]Micturition frequency and polyuria	Symptom	CPRD GOLD
53830		Polyuria	Symptom	CPRD GOLD
63898		[D]Frequency of micturition or polyuria NOS	Symptom	CPRD GOLD

Supplementary Table 10: UTI prescription codes

prodcodeid	Product Name	Drug	Formulation	Route of Administration	database
1091741000033110	Pivmecillinam 200mg tablets	Pivmecillinam hydrochloride	Tablet	Oral	CPRD Aurum
1275241000033110	Selexid 200mg tablets	Pivmecillinam hydrochloride	Tablet	Oral	CPRD Aurum
936041000033112	Monotrim 100mg tablets	Trimethoprim	Tablet	Oral	CPRD Aurum
936141000033111	Monotrim 200mg tablets	Trimethoprim	Tablet	Oral	CPRD Aurum
933741000033116	Monotrim 50mg/5ml oral suspension	Trimethoprim	Oral suspension	Oral	CPRD Aurum
1467541000033110	Trimethoprim 50mg/5ml oral suspension sugar free	Trimethoprim	Oral suspension	Oral	CPRD Aurum
1472841000033110	Trimopan 100mg tablets	Trimethoprim	Tablet	Oral	CPRD Aurum
1472941000033110	Trimopan 200mg tablets	Trimethoprim	Tablet	Oral	CPRD Aurum
8946441000033110	Fosfomycin 500mg capsules	Fosfomycin calcium	Capsule	Oral	CPRD Aurum
599941000033116	Fosfomycin 3g granules sachets	Fosfomycin trometamol	Granules	Oral	CPRD Aurum
925241000033114	Monuril 3g granules sachets	`	Granules	Oral	CPRD Aurum
670841000033118	Hiprex 1g tablets	Methenamine hippurate	Tablet	Oral	CPRD Aurum
619141000033114	Furadantin 100mg tablets	Nitrofurantoin	Tablet	Oral	CPRD Aurum
618541000033112	Furadantin 25mg/5ml oral suspension	Nitrofurantoin	Oral suspension	Oral	CPRD Aurum
619241000033119	Furadantin 50mg tablets	Nitrofurantoin	Tablet	Oral	CPRD Aurum
11603941000033100	Genfura 100mg tablets	Nitrofurantoin	Tablet	Oral	CPRD Aurum
11604041000033100	Genfura 50mg tablets	Nitrofurantoin	Tablet	Oral	CPRD Aurum
863441000033113	Macrobid 100mg modified-release capsules	Nitrofurantoin	Modified-release capsule	Oral	CPRD Aurum
857841000033111	Macrodantin 100mg capsules	Nitrofurantoin	Capsule	Oral	CPRD Aurum
857941000033115	Macrodantin 50mg capsules	Nitrofurantoin	Capsule	Oral	CPRD Aurum
971641000033114	Nitrofurantoin 100mg modified-release capsules	Nitrofurantoin	Modified-release capsule	Oral	CPRD Aurum
5997141000033110	Nitrofurantoin 25mg/5ml oral solution	Nitrofurantoin	Oral solution	Oral	CPRD Aurum
974441000033110	Nitrofurantoin 25mg/5ml oral suspension sugar free	Nitrofurantoin	Oral suspension	Oral	CPRD Aurum
2955441000033110	Urantoin 100mg tablets	Nitrofurantoin	Tablet	Oral	CPRD Aurum
6168141000033110	Sodium citrate 1.5g/5ml oral solution	Sodium citrate	Oral solution	Oral	CPRD Aurum
1362441000033110	Sodium citrate powder	Sodium citrate	Not applicable	Not applicable	CPRD Aurum
**prodcode**	**productname**	**drugsubstance**	**BNF Chapter**	**route**	**Database**
21029	Miraxid 450 Tablet (Rpr / Fisons)	Pivampicillin/Pivmecillinam Hydrochloride	Broad-spectrum Penicillins	Oral	CPRD GOLD
20516	Miraxid Liquid (Rpr / Fisons)	Pivampicillin/Pivmecillinam Hydrochloride	Broad-spectrum Penicillins	Oral	CPRD GOLD
17161	Miraxid Tablet (Rpr / Fisons)	Pivampicillin/Pivmecillinam Hydrochloride	Broad-spectrum Penicillins	Oral	CPRD GOLD
25832	Pivampicillin 125mg with pivmecillinam100mg tablet	Pivampicillin/Pivmecillinam Hydrochloride	Broad-spectrum Penicillins	Oral	CPRD GOLD
12540	Pivampicillin 250mg with pivmecillinam 200mg tablet	Pivampicillin/Pivmecillinam Hydrochloride	Broad-spectrum Penicillins	Oral	CPRD GOLD
8960	Pondocillin plus Tablet (Edwin Burgess Ltd)	Pivampicillin/Pivmecillinam Hydrochloride	Broad-spectrum Penicillins	Oral	
12014	Pivmecillinam 200mg tablets	Pivmecillinam hydrochloride	Mecillinams	Oral	CPRD GOLD

68786	Pivmecillinam 200mg/5ml oral suspension	Pivmecillinam hydrochloride	Mecillinams	Oral	CPRD GOLD
9601	Selexid 200mg tablets (LEO Pharma)	Pivmecillinam hydrochloride	Mecillinams	Oral	CPRD GOLD
26101	Pivmecillinam 100mg/sachet	Pivmecillinam Hydrochloride	Mecillinams/Urinary -tract Infections	Oral	CPRD GOLD
12015	Selexid 100mg/sachet Liquid (Edwin Burgess Ltd)	Pivmecillinam Hydrochloride	Mecillinams/Urinary -tract Infections	Oral	CPRD GOLD
372	Nalidixic acid 300mg/5ml oral suspension	Nalidixic acid	Quinolones	Oral	CPRD GOLD
21147	Uriben 300mg/5ml oral suspension (Rosemont Pharmaceuticals Ltd)	Nalidixic acid	Quinolones	Oral	CPRD GOLD
9073	Nalidixic acid with sodium citrate 660mg + 3750mg Sachets	Nalidixic Acid/Sodium Citrate	Quinolones	Oral	CPRD GOLD
13325	Monotrim 100mg tablets (Abbott Healthcare Products Ltd)	Trimethoprim	Sulphonamides And Trimethoprim	Oral	CPRD GOLD
8171	Monotrim 200mg tablets (Abbott Healthcare Products Ltd)	Trimethoprim	Sulphonamides And Trimethoprim	Oral	CPRD GOLD
36622	Monotrim 50mg/5ml oral suspension (Chemidex Pharma Ltd)	Trimethoprim	Sulphonamides And Trimethoprim	Oral	CPRD GOLD
340	Trimethoprim 100mg tablets	Trimethoprim	Sulphonamides And Trimethoprim	Oral	CPRD GOLD
32906	Trimethoprim 100mg tablets (A A H Pharmaceuticals Ltd)	Trimethoprim	Sulphonamides And Trimethoprim	Oral	CPRD GOLD
43545	Trimethoprim 100mg tablets (Actavis UK Ltd)	Trimethoprim	Sulphonamides And Trimethoprim	Oral	CPRD GOLD
58490	Trimethoprim 100mg tablets (Alliance Healthcare (Distribution) Ltd)	Trimethoprim	Sulphonamides And Trimethoprim	Oral	CPRD GOLD
53720	Trimethoprim 100mg tablets (Almus Pharmaceuticals Ltd)	Trimethoprim	Sulphonamides And Trimethoprim	Oral	CPRD GOLD
56267	Trimethoprim 100mg tablets (Bristol Laboratories Ltd)	Trimethoprim	Sulphonamides And Trimethoprim	Oral	CPRD GOLD
68225	Trimethoprim 100mg tablets (Crescent Pharma Ltd)	Trimethoprim	Sulphonamides And Trimethoprim	Oral	CPRD GOLD
67596	Trimethoprim 100mg tablets (DE Pharmaceuticals)	Trimethoprim	Sulphonamides And Trimethoprim	Oral	CPRD GOLD
34488	Trimethoprim 100mg tablets (Kent Pharmaceuticals Ltd)	Trimethoprim	Sulphonamides And Trimethoprim	Oral	CPRD GOLD
67147	Trimethoprim 100mg tablets (Mawdsley-Brooks & Company Ltd)	Trimethoprim	Sulphonamides And Trimethoprim	Oral	CPRD GOLD
49592	Trimethoprim 100mg tablets (Phoenix Healthcare Distribution Ltd)	Trimethoprim	Sulphonamides And Trimethoprim	Oral	CPRD GOLD
45246	Trimethoprim 100mg tablets (Sandoz Ltd)	Trimethoprim	Sulphonamides And Trimethoprim	Oral	CPRD GOLD
56259	Trimethoprim 100mg tablets (Sigma Pharmaceuticals Plc)	Trimethoprim	Sulphonamides And Trimethoprim	Oral	CPRD GOLD
34542	Trimethoprim 100mg tablets (Teva UK Ltd)	Trimethoprim	Sulphonamides And Trimethoprim	Oral	CPRD GOLD
57080	Trimethoprim 100mg tablets (Waymade Healthcare Plc)	Trimethoprim	Sulphonamides And Trimethoprim	Oral	CPRD GOLD
37	Trimethoprim 200mg tablets	Trimethoprim	Sulphonamides And Trimethoprim	Oral	CPRD GOLD
27255	Trimethoprim 200mg tablets (A A H Pharmaceuticals Ltd)	Trimethoprim	Sulphonamides And Trimethoprim	Oral	CPRD GOLD
50120	Trimethoprim 200mg tablets (Accord Healthcare Ltd)	Trimethoprim	Sulphonamides And Trimethoprim	Oral	CPRD GOLD
34392	Trimethoprim 200mg tablets (Actavis UK Ltd)	Trimethoprim	Sulphonamides And Trimethoprim	Oral	CPRD GOLD
50797	Trimethoprim 200mg tablets (Alliance Healthcare (Distribution) Ltd)	Trimethoprim	Sulphonamides And Trimethoprim	Oral	CPRD GOLD
39933	Trimethoprim 200mg tablets (Almus Pharmaceuticals Ltd)	Trimethoprim	Sulphonamides And Trimethoprim	Oral	CPRD GOLD
51510	Trimethoprim 200mg tablets (Bristol Laboratories Ltd)	Trimethoprim	Sulphonamides And Trimethoprim	Oral	CPRD GOLD
64028	Trimethoprim 200mg tablets (Crescent Pharma Ltd)	Trimethoprim	Sulphonamides And Trimethoprim	Oral	CPRD GOLD

65487	Trimethoprim 200mg tablets (DE Pharmaceuticals)	Trimethoprim	Sulphonamides And Trimethoprim	Oral	CPRD GOLD
33997	Trimethoprim 200mg tablets (IVAX Pharmaceuticals UK Ltd)	Trimethoprim	Sulphonamides And Trimethoprim	Oral	CPRD GOLD
34379	Trimethoprim 200mg tablets (Kent Pharmaceuticals Ltd)	Trimethoprim	Sulphonamides And Trimethoprim	Oral	CPRD GOLD
65497	Trimethoprim 200mg tablets (Mawdsley-Brooks & Company Ltd)	Trimethoprim	Sulphonamides And Trimethoprim	Oral	CPRD GOLD
53599	Trimethoprim 200mg tablets (Phoenix Healthcare Distribution Ltd)	Trimethoprim	Sulphonamides And Trimethoprim	Oral	CPRD GOLD
62630	Trimethoprim 200mg tablets (Ranbaxy (UK) Ltd)	Trimethoprim	Sulphonamides And Trimethoprim	Oral	CPRD GOLD
34633	Trimethoprim 200mg tablets (Sandoz Ltd)	Trimethoprim	Sulphonamides And Trimethoprim	Oral	CPRD GOLD
32908	Trimethoprim 200mg tablets (Teva UK Ltd)	Trimethoprim	Sulphonamides And Trimethoprim	Oral	CPRD GOLD
57981	Trimethoprim 200mg tablets (Waymade Healthcare Plc)	Trimethoprim	Sulphonamides And Trimethoprim	Oral	CPRD GOLD
52669	Trimethoprim 200mg/5ml oral solution	Trimethoprim	Sulphonamides And Trimethoprim	Oral	CPRD GOLD
55986	Trimethoprim 200mg/5ml oral suspension	Trimethoprim	Sulphonamides And Trimethoprim	Oral	CPRD GOLD
57642	Trimethoprim 20mg/5ml oral solution	Trimethoprim	Sulphonamides And Trimethoprim	Oral	CPRD GOLD
51725	Trimethoprim 20mg/5ml oral suspension	Trimethoprim	Sulphonamides And Trimethoprim	Oral	CPRD GOLD
477	Trimethoprim 50mg/5ml oral suspension sugar free	Trimethoprim	Sulphonamides And Trimethoprim	Oral	CPRD GOLD
34252	Trimethoprim 50mg/5ml oral suspension sugar free (A A H Pharmaceuticals Ltd)	Trimethoprim	Sulphonamides And Trimethoprim	Oral	CPRD GOLD
53828	Trimethoprim 50mg/5ml oral suspension sugar free (Actavis UK Ltd)	Trimethoprim	Sulphonamides And Trimethoprim	Oral	CPRD GOLD
53275	Trimethoprim 50mg/5ml oral suspension sugar free (Alliance Healthcare (Distribution) Ltd)	Trimethoprim	Sulphonamides And Trimethoprim	Oral	CPRD GOLD
60808	Trimethoprim 50mg/5ml oral suspension sugar free (Almus Pharmaceuticals Ltd)	Trimethoprim	Sulphonamides And Trimethoprim	Oral	CPRD GOLD
53276	Trimethoprim 50mg/5ml oral suspension sugar free (Kent Pharmaceuticals Ltd)	Trimethoprim	Sulphonamides And Trimethoprim	Oral	CPRD GOLD
67361	Trimethoprim 50mg/5ml oral suspension sugar free (Phoenix Healthcare Distribution Ltd)	Trimethoprim	Sulphonamides And Trimethoprim	Oral	CPRD GOLD
61714	Trimethoprim 50mg/5ml oral suspension sugar free (Pinewood Healthcare)	Trimethoprim	Sulphonamides And Trimethoprim	Oral	CPRD GOLD
53284	Trimethoprim 50mg/5ml oral suspension sugar free (Sigma Pharmaceuticals Plc)	Trimethoprim	Sulphonamides And Trimethoprim	Oral	CPRD GOLD
29351	Trimethoprim 50mg/5ml oral suspension sugar free (Teva UK Ltd)	Trimethoprim	Sulphonamides And Trimethoprim	Oral	CPRD GOLD
57116	Trimethoprim 50mg/5ml oral suspension sugar free (Waymade Healthcare Plc)	Trimethoprim	Sulphonamides And Trimethoprim	Oral	CPRD GOLD
21640	Trimopan 100mg tablets (Teva UK Ltd)	Trimethoprim	Sulphonamides And Trimethoprim	Oral	CPRD GOLD
10046	Trimopan 200mg tablets (Teva UK Ltd)	Trimethoprim	Sulphonamides And Trimethoprim	Oral	CPRD GOLD
15081	Ipral 100mg Tablet (E R Squibb and Sons Ltd)	Trimethoprim	Sulphonamides And Trimethoprim/Urinary-tract Infections	Oral	CPRD GOLD
14998	Ipral 200mg Tablet (E R Squibb and Sons Ltd)	Trimethoprim	Sulphonamides And Trimethoprim/Urinary-tract Infections	Oral	CPRD GOLD
14367	Ipral 50mg/5ml Liquid (E R Squibb and Sons Ltd)	Trimethoprim	Sulphonamides And Trimethoprim/Urinary-tract Infections	Oral	CPRD GOLD

280	Monotrim 50mg/5ml Liquid (Solvay Healthcare)	Trimethoprim	Sulphonamides And Trimethoprim/Urinary-tract Infections	Oral	CPRD GOLD
25497	Syraprim 100mg Tablet (Wellcome Medical Division)	Trimethoprim	Sulphonamides And Trimethoprim/Urinary-tract Infections	Oral	CPRD GOLD
27048	Syraprim 300mg Tablet (Wellcome Medical Division)	Trimethoprim	Sulphonamides And Trimethoprim/Urinary-tract Infections	Oral	CPRD GOLD
34878	Trimethoprim 100mg Tablet (C P Pharmaceuticals Ltd)	Trimethoprim	Sulphonamides And Trimethoprim/Urinary-tract Infections	Oral	CPRD GOLD
41544	Trimethoprim 100mg Tablet (IVAX Pharmaceuticals UK Ltd)	Trimethoprim	Sulphonamides And Trimethoprim/Urinary-tract Infections	Oral	CPRD GOLD
34455	Trimethoprim 200mg Tablet (C P Pharmaceuticals Ltd)	Trimethoprim	Sulphonamides And Trimethoprim/Urinary-tract Infections	Oral	CPRD GOLD
43537	Trimethoprim 200mg Tablet (Celltech Pharma Europe Ltd)	Trimethoprim	Sulphonamides And Trimethoprim/Urinary-tract Infections	Oral	CPRD GOLD
43505	Trimethoprim 200mg Tablet (Numark Management Ltd)	Trimethoprim	Sulphonamides And Trimethoprim/Urinary-tract Infections	Oral	CPRD GOLD
31227	Trimethoprim 200mg Tablet (Regent Laboratories Ltd)	Trimethoprim	Sulphonamides And Trimethoprim/Urinary-tract Infections	Oral	CPRD GOLD
8073	Trimethoprim 300mg Tablet	Trimethoprim	Sulphonamides And Trimethoprim/Urinary-tract Infections	Oral	CPRD GOLD
29532	Trimogal 100mg Tablet (Lagap)	Trimethoprim	Sulphonamides And Trimethoprim/Urinary-tract Infections	Oral	CPRD GOLD
24324	Trimogal 200mg Tablet (Lagap)	Trimethoprim	Sulphonamides And Trimethoprim/Urinary-tract Infections	Oral	CPRD GOLD
7616	Trimopan 50mg/5ml Liquid (Berk Pharmaceuticals Ltd)	Trimethoprim	Sulphonamides And Trimethoprim/Urinary-tract Infections	Oral	CPRD GOLD
21805	Triprimix 200 Tablet (Ashbourne Pharmaceuticals Ltd)	Trimethoprim	Sulphonamides And Trimethoprim/Urinary-tract Infections	Oral	CPRD GOLD
26113	Fosfomycin 2g Sachets	Fosfomycin	Urinary-tract Infections	Oral	CPRD GOLD
27986	Monuril 2g Paediatric sachet (Pharmax Ltd)	Fosfomycin	Urinary-tract Infections	Oral	CPRD GOLD
55112	Fosfomycin 500mg capsules	Fosfomycin calcium	Urinary-tract Infections	Oral	CPRD GOLD
21487	Fosfomycin 3g granules sachets	Fosfomycin trometamol	Urinary-tract Infections	Oral	CPRD GOLD
71169	Fosfomycin 3g granules sachets (AMCo)	Fosfomycin trometamol	Urinary-tract Infections	Oral	CPRD GOLD
64501	Monuril 3g granules sachets (Lexon (UK) Ltd)	Fosfomycin trometamol	Urinary-tract Infections	Oral	CPRD GOLD
68216	Monuril 3g granules sachets (Zambon S.p.A.)	Fosfomycin trometamol	Urinary-tract Infections	Oral	CPRD GOLD
12379	Monuril 3g Sachets (Pharmax Ltd)	Fosfomycin trometamol	Urinary-tract Infections	Oral	CPRD GOLD
4282	Hiprex 1g tablets (Meda Pharmaceuticals Ltd)	Methenamine hippurate	Urinary-tract Infections	Oral	CPRD GOLD
4290	Methenamine hippurate 1g tablets	Methenamine hippurate	Urinary-tract Infections	Oral	CPRD GOLD
29795	Methenamine hippurate 300mg Tablet	Methenamine Hippurate	Urinary-tract Infections	Oral	CPRD GOLD
2541	Furadantin 100mg tablets (AMCo)	Nitrofurantoin	Urinary-tract Infections	Oral	CPRD GOLD
272	Furadantin 25mg/5ml oral suspension (Mercury Pharma Group Ltd)	Nitrofurantoin	Urinary-tract Infections	Oral	CPRD GOLD
2023	Furadantin 50mg tablets (AMCo)	Nitrofurantoin	Urinary-tract Infections	Oral	CPRD GOLD
67981	Genfura 100mg tablets (Genesis Pharmaceuticals Ltd)	Nitrofurantoin	Urinary-tract Infections	Oral	CPRD GOLD

69434	Genfura 50mg tablets (Genesis Pharmaceuticals Ltd)	Nitrofurantoin	Urinary-tract Infections	Oral	CPRD GOLD
7525	Macrobid 100mg modified-release capsules (AMCo)	Nitrofurantoin	Urinary-tract Infections	Oral	CPRD GOLD
65803	Macrobid 100mg modified-release capsules (Waymade Healthcare Plc)	Nitrofurantoin	Urinary-tract Infections	Oral	CPRD GOLD
2036	Macrodantin 100mg capsules (AMCo)	Nitrofurantoin	Urinary-tract Infections	Oral	CPRD GOLD
1825	Macrodantin 50mg capsules (AMCo)	Nitrofurantoin	Urinary-tract Infections	Oral	CPRD GOLD
65251	Macrodantin 50mg capsules (Waymade Healthcare Plc)	Nitrofurantoin	Urinary-tract Infections	Oral	CPRD GOLD
466	Nitrofurantoin 100mg capsules	Nitrofurantoin	Urinary-tract Infections	Oral	CPRD GOLD
61642	Nitrofurantoin 100mg capsules (Alliance Healthcare (Distribution) Ltd)	Nitrofurantoin	Urinary-tract Infections	Oral	CPRD GOLD
60713	Nitrofurantoin 100mg capsules (AMCo)	Nitrofurantoin	Urinary-tract Infections	Oral	CPRD GOLD
6370	Nitrofurantoin 100mg modified-release capsules	Nitrofurantoin	Urinary-tract Infections	Oral	CPRD GOLD
2887	Nitrofurantoin 100mg tablets	Nitrofurantoin	Urinary-tract Infections	Oral	CPRD GOLD
35850	Nitrofurantoin 100mg tablets (A A H Pharmaceuticals Ltd)	Nitrofurantoin	Urinary-tract Infections	Oral	CPRD GOLD
41397	Nitrofurantoin 100mg tablets (Actavis UK Ltd)	Nitrofurantoin	Urinary-tract Infections	Oral	CPRD GOLD
70380	Nitrofurantoin 100mg tablets (Mawdsley-Brooks & Company Ltd)	Nitrofurantoin	Urinary-tract Infections	Oral	CPRD GOLD
67762	Nitrofurantoin 100mg tablets (Mylan)	Nitrofurantoin	Urinary-tract Infections	Oral	CPRD GOLD
53638	Nitrofurantoin 100mg tablets (Teva UK Ltd)	Nitrofurantoin	Urinary-tract Infections	Oral	CPRD GOLD
64690	Nitrofurantoin 100mg/5ml oral solution	Nitrofurantoin	Urinary-tract Infections	Oral	CPRD GOLD
65207	Nitrofurantoin 24mg/5ml oral suspension	Nitrofurantoin	Urinary-tract Infections	Oral	CPRD GOLD
56621	Nitrofurantoin 25mg/5ml oral solution	Nitrofurantoin	Urinary-tract Infections	Oral	CPRD GOLD
2198	Nitrofurantoin 25mg/5ml Oral suspension	Nitrofurantoin	Urinary-tract Infections	Oral	CPRD GOLD
53659	Nitrofurantoin 25mg/5ml oral suspension	Nitrofurantoin	Urinary-tract Infections	Oral	CPRD GOLD
48353	Nitrofurantoin 25mg/5ml oral suspension sugar free	Nitrofurantoin	Urinary-tract Infections	Oral	CPRD GOLD
69479	Nitrofurantoin 25mg/5ml oral suspension sugar free (Alliance Healthcare (Distribution) Ltd)	Nitrofurantoin	Urinary-tract Infections	Oral	CPRD GOLD
35673	Nitrofurantoin 25mg/5ml oral suspension sugar free (AMCo)	Nitrofurantoin	Urinary-tract Infections	Oral	CPRD GOLD
64389	Nitrofurantoin 30mg/5ml oral solution	Nitrofurantoin	Urinary-tract Infections	Oral	CPRD GOLD
68651	Nitrofurantoin 30mg/5ml oral suspension	Nitrofurantoin	Urinary-tract Infections	Oral	CPRD GOLD
60795	Nitrofurantoin 35mg/5ml oral solution	Nitrofurantoin	Urinary-tract Infections	Oral	CPRD GOLD
51726	Nitrofurantoin 40mg/5ml oral suspension	Nitrofurantoin	Urinary-tract Infections	Oral	CPRD GOLD
210	Nitrofurantoin 50mg capsules	Nitrofurantoin	Urinary-tract Infections	Oral	CPRD GOLD
63588	Nitrofurantoin 50mg capsules (A A H Pharmaceuticals Ltd)	Nitrofurantoin	Urinary-tract Infections	Oral	CPRD GOLD
61907	Nitrofurantoin 50mg capsules (Alliance Healthcare (Distribution) Ltd)	Nitrofurantoin	Urinary-tract Infections	Oral	CPRD GOLD
60252	Nitrofurantoin 50mg capsules (AMCo)	Nitrofurantoin	Urinary-tract Infections	Oral	CPRD GOLD
62647	Nitrofurantoin 50mg Tablet (Biorex Laboratories Ltd)	Nitrofurantoin	Urinary-tract Infections	Oral	CPRD GOLD
778	Nitrofurantoin 50mg tablets	Nitrofurantoin	Urinary-tract Infections	Oral	CPRD GOLD

53094	Nitrofurantoin 50mg tablets (A A H Pharmaceuticals Ltd)	Nitrofurantoin	Urinary-tract Infections	Oral	CPRD GOLD
40164	Nitrofurantoin 50mg tablets (Actavis UK Ltd)	Nitrofurantoin	Urinary-tract Infections	Oral	CPRD GOLD
51959	Nitrofurantoin 50mg tablets (Alliance Healthcare (Distribution) Ltd)	Nitrofurantoin	Urinary-tract Infections	Oral	CPRD GOLD
66013	Nitrofurantoin 50mg tablets (Almus Pharmaceuticals Ltd)	Nitrofurantoin	Urinary-tract Infections	Oral	CPRD GOLD
53171	Nitrofurantoin 50mg tablets (Dr Reddy's Laboratories (UK) Ltd)	Nitrofurantoin	Urinary-tract Infections	Oral	CPRD GOLD
57669	Nitrofurantoin 50mg tablets (Genesis Pharmaceuticals Ltd)	Nitrofurantoin	Urinary-tract Infections	Oral	CPRD GOLD
67759	Nitrofurantoin 50mg tablets (Mylan)	Nitrofurantoin	Urinary-tract Infections	Oral	CPRD GOLD
54325	Nitrofurantoin 50mg tablets (Phoenix Healthcare Distribution Ltd)	Nitrofurantoin	Urinary-tract Infections	Oral	CPRD GOLD
70796	Nitrofurantoin 50mg tablets (Teva UK Ltd)	Nitrofurantoin	Urinary-tract Infections	Oral	CPRD GOLD
57779	Nitrofurantoin 50mg tablets (Waymade Healthcare Plc)	Nitrofurantoin	Urinary-tract Infections	Oral	CPRD GOLD
58469	Nitrofurantoin 5mg/5ml oral solution	Nitrofurantoin	Urinary-tract Infections	Oral	CPRD GOLD
57248	Nitrofurantoin 5mg/5ml oral suspension	Nitrofurantoin	Urinary-tract Infections	Oral	CPRD GOLD
59497	Nitrofurantoin 7mg/5ml oral suspension	Nitrofurantoin	Urinary-tract Infections	Oral	CPRD GOLD
67095	Nitrofurantoin 9mg/5ml oral suspension	Nitrofurantoin	Urinary-tract Infections	Oral	CPRD GOLD
16284	Urantoin 100mg tablets (Dr Reddy's Laboratories (UK) Ltd)	Nitrofurantoin	Urinary-tract Infections	Oral	CPRD GOLD
61709	Sodium citrate 1.5g/5ml oral solution	Sodium citrate	Sodium Citrate/Urinary-tract Infections	Oral	CPRD GOLD
24146	Sodium citrate powder	Sodium citrate	Sodium Citrate/Urinary-tract Infections/Alkalinisa tion Of Urine/Extemporaneo us Item	Route of administrat ion not applicable	CPRD GOLD

Supplementary Table 11: Smoking status codes

medcodeid	snomedctconceptid	term	smokingstatus	database
78013015	46802002	Smokers' cough	current smoker	CPRD Aurum
128130017	77176002	Smoker	current smoker	CPRD Aurum
216212011	134406006	Smoking reduced	current smoker	CPRD Aurum
338608011	225323000	Smoking cessation advice	current smoker	CPRD Aurum
342443012	228377000	Smokes drugs	current smoker	CPRD Aurum
342444018	228378005	Smokes drugs in cigarette form	current smoker	CPRD Aurum
342445017	228379002	Smokes drugs through a pipe	current smoker	CPRD Aurum
1484932017	390900001	Smoking cessation milestones	current smoker	CPRD Aurum
1484934016	390902009	Smoking status at 4 weeks	current smoker	CPRD Aurum
1484935015	390903004	Smoking status between 4 and 52 weeks	current smoker	CPRD Aurum
1484936019	390904005	Smoking status at 52 weeks	current smoker	CPRD Aurum
1773560017	401160008	Smoking cessation programme start date	current smoker	CPRD Aurum
2669652019	266929003	Smoking started	current smoker	CPRD Aurum
2670126018	308438006	Smoking restarted	current smoker	CPRD Aurum
137711000006111	137711000006107	Smoker (Read codes)	current smoker	CPRD Aurum
137721000006115	266918002	Smoker - amount smoked	current smoker	CPRD Aurum
137771000006119	137771000006103	Smoking Age Started	current smoker	CPRD Aurum
137791000006118	308438006	Smoking restarted	current smoker	CPRD Aurum
303501000000115	200221000000105	Smoking cessation advice provided by community pharmacist	current smoker	CPRD Aurum
482771000000118	713700008	Smoking cessation drug therapy	current smoker	CPRD Aurum
489931000000114	710081004	Smoking cessation therapy NOS	current smoker	CPRD Aurum
492511000000117	710081004	Smoking cessation therapy	current smoker	CPRD Aurum
736241000000116	374361000000100	Smoking cessation enhanced services administration	current smoker	CPRD Aurum
818061000006111	225323000	Smoking cessation education	current smoker	CPRD Aurum
852131000006112	852131000006108	Smoking cessation-practice nurse support	current smoker	CPRD Aurum
904071000006117	904071000006101	Smoking cessation 4 week follow up	current smoker	CPRD Aurum
904081000006119	904081000006103	Smoking cessation 52 week follow up	current smoker	CPRD Aurum
904141000006112	904141000006108	Smoking cessation 4 week F/U not completed	current smoker	CPRD Aurum
904151000006114	904151000006105	Smoking cessation 4 week F/U completed	current smoker	CPRD Aurum
904161000006111	904161000006107	Smoking cessation 52 week F/U completed	current smoker	CPRD Aurum
904171000006116	904171000006100	Smoking cessation 52 week F/U not completed	current smoker	CPRD Aurum
904181000006118	904181000006102	Smoking cessation counselling	current smoker	CPRD Aurum
904191000006115	904191000006104	Smoking cessation motivation score	current smoker	CPRD Aurum
904201000006117	904201000006101	Smoking cessation confidence score	current smoker	CPRD Aurum
904221000006110	904221000006106	Smoking cessation bupropion therapy	current smoker	CPRD Aurum
904231000006113	904231000006109	Smoking cessation counselling by telephone	current smoker	CPRD Aurum
904241000006115	904241000006104	Smoking cessation counselling in person	current smoker	CPRD Aurum
961581000006114	961581000006105	Smokes/uses tobacco products	current smoker	CPRD Aurum
1175011000000110	1087441000000100	Smoking cessation programme declined	current smoker	CPRD Aurum
1176421000000110	527151000000107	Smoking cessation advice declined	current smoker	CPRD Aurum
1538681000006110	1538681000006100	Smoke	current smoker	CPRD Aurum
1591651000006110	1591651000006100	Smoking cessation referral declined	current smoker	CPRD Aurum
1626121000006110	1626121000006100	Smoking cessation drug therapy - varenicline	current smoker	CPRD Aurum

1704491000006110	374391000000106	Smoking cessation enhanced services administration monitoring template completed	current smoker	CPRD Aurum
1708051000000110	766931000000106	Smoking status at 12 weeks	current smoker	CPRD Aurum
1741641000006110	374391000000106	Smoking cessation ESA monitoring template completed	current smoker	CPRD Aurum
1746211000006110	1746211000006100	Smoking status at 12 weeks	current smoker	CPRD Aurum
1819411000006110	1819411000006100	Smoking increased	current smoker	CPRD Aurum
1823811000006110	1823811000006100	Smoking cessation 12 week follow up	current smoker	CPRD Aurum
1825071000006110	1825071000006100	Smoking cessation 12 week F/U not completed	current smoker	CPRD Aurum
1825081000006110	1825081000006100	Smoking cessation 12 week F/U completed	current smoker	CPRD Aurum
1979491000006110	1979491000006100	Smoking cessation follow-up	current smoker	CPRD Aurum
2141561000000110	822591000000108	Smoking cessation drug therapy declined	current smoker	CPRD Aurum
2203381000000110	850331000000104	Smoking cessation 12 week follow-up	current smoker	CPRD Aurum
2690191000006110	11947005	Smoking-pipe maker	current smoker	CPRD Aurum
3544141000006110	64197008	Smoke	current smoker	CPRD Aurum
4948531000006110	225934006	Smokes in bed	current smoker	CPRD Aurum
5074201000006110	235033006	Smokers keratosis	current smoker	CPRD Aurum
5979541000006110	308511002	Smoking monitoring call	current smoker	CPRD Aurum
5979551000006110	308512009	Smoking monitoring status	current smoker	CPRD Aurum
5999811000006110	310429001	Smoking monitoring invitation	current smoker	CPRD Aurum
6282351000006110	365981007	Smoking	current smoker	CPRD Aurum
6427931000006110	384742004	Smoking cessation assistance	current smoker	CPRD Aurum
7368651000006110	449345000	Smoked before confirmation of pregnancy	current smoker	CPRD Aurum
7375991000006110	449868002	Smokes tobacco daily	current smoker	CPRD Aurum
7504551000006110	698101006	Smoking device	current smoker	CPRD Aurum
8196151000006110	521721000000109	Smoking cessation leaflet given	current smoker	CPRD Aurum
8221521000006110	720401000000103	Smoking cessation	current smoker	CPRD Aurum
11793611000000000	384742004	Smoking cessation behaviour support	current smoker	CPRD Aurum
14616531000000000	77176002	Smoking	current smoker	CPRD Aurum
137761000006114	137761000006105	Smoking Age Ceased	ex-smoker	CPRD Aurum
852121000006114	852121000006105	Smoking cessation-maintain abstinence	ex-smoker	CPRD Aurum
904011000006114	904011000006105	Smoking cessation declaration signed by patient	ex-smoker	CPRD Aurum
1009271000006110	1009271000006100	Non Smoker - Nos	non-smoker	CPRD Aurum
1123751000000110	505681000000109	Non-smoker annual review - enhanced services administration	non-smoker	CPRD Aurum
1154431000000110	505681000000109	Non-smoker annual review	non-smoker	CPRD Aurum
14866014	8392000	Non-smoker	non-smoker	CPRD Aurum
250374013	160618006	Current non-smoker	non-smoker	CPRD Aurum
397732011	266919005	Never smoked tobacco	non-smoker	CPRD Aurum
5495921000006110	266919005	Never smoked	non-smoker	CPRD Aurum
6718071000006110	405746006	Current non smoker but past smoking history unknown	non-smoker	CPRD Aurum
7965041000006110	221000119102	Never smoked any substance	non-smoker	CPRD Aurum
854951000006113	854951000006109	Grade A non-smoker	non-smoker	CPRD Aurum
904111000006113	904111000006109	Carbon monoxide validation confirms non-smoker	non-smoker	CPRD Aurum
	**readcode**	**readcode_desc**	**smokingstatus**	**database**
	1371	Never smoked tobacco	non-smoker	CPRD GOLD
	1371.11	Non-smoker	non-smoker	CPRD GOLD

	T5093N	SMOKED NEVER	non-smoker	CPRD GOLD
	1377	Ex-trivial smoker (<1/day)	ex-smoker	CPRD GOLD
	1378	Ex-light smoker (1-9/day)	ex-smoker	CPRD GOLD
	1379	Ex-moderate smoker (10-19/day)	ex-smoker	CPRD GOLD
	137A.00	Ex-heavy smoker (20-39/day)	ex-smoker	CPRD GOLD
	137B.00	Ex-very heavy smoker (40+/day)	ex-smoker	CPRD GOLD
	137F.00	Ex-smoker - amount unknown	ex-smoker	CPRD GOLD
	137K.00	Stopped smoking	ex-smoker	CPRD GOLD
	137L.00	Current non-smoker	non-smoker	CPRD GOLD
	137N.00	Ex pipe smoker	ex-smoker	CPRD GOLD
	137O.00	Ex cigar smoker	ex-smoker	CPRD GOLD
	137S.00	Ex smoker	ex-smoker	CPRD GOLD
	137T.00	Date ceased smoking	ex-smoker	CPRD GOLD
	T5091	STOPPED SMOKING	ex-smoker	CPRD GOLD
	T5091ES	FORMER SMOKER	ex-smoker	CPRD GOLD
	T5091HS	EX HEAVY SMOKER	ex-smoker	CPRD GOLD
	T5093	SMOKER NON	non-smoker	CPRD GOLD
	137..11	Smoker - amount smoked	current smoker	CPRD GOLD
	1372	Trivial smoker - < 1 cig/day	current smoker	CPRD GOLD
	1372.11	Occasional smoker	current smoker	CPRD GOLD
	1373	Light smoker - 1-9 cigs/day	current smoker	CPRD GOLD
	1374	Moderate smoker - 10-19 cigs/d	current smoker	CPRD GOLD
	1375	Heavy smoker - 20-39 cigs/day	current smoker	CPRD GOLD
	1376	Very heavy smoker - 40+cigs/d	current smoker	CPRD GOLD
	137C.00	Keeps trying to stop smoking	current smoker	CPRD GOLD
	137D.00	Admitted tobacco cons untrue ?	current smoker	CPRD GOLD
	137G.00	Trying to give up smoking	current smoker	CPRD GOLD
	137H.00	Pipe smoker	current smoker	CPRD GOLD
	137J.00	Cigar smoker	current smoker	CPRD GOLD
	137M.00	Rolls own cigarettes	current smoker	CPRD GOLD
	137P.00	Cigarette smoker	current smoker	CPRD GOLD
	137P.11	Smoker	current smoker	CPRD GOLD
	137Q.00	Smoking started	current smoker	CPRD GOLD
	137Q.11	Smoking restarted	current smoker	CPRD GOLD
	137R.00	Current smoker	current smoker	CPRD GOLD
	137V.00	Smoking reduced	current smoker	CPRD GOLD
	137X.00	Cigarette consumption	current smoker	CPRD GOLD
	137Y.00	Cigar consumption	current smoker	CPRD GOLD
	137Z.00	Tobacco consumption NOS	current smoker	CPRD GOLD
	137a.00	Pipe tobacco consumption	current smoker	CPRD GOLD
	137b.00	Ready to stop smoking	current smoker	CPRD GOLD
	137c.00	Thinking about stopping smoking	current smoker	CPRD GOLD
	137d.00	Not interested in stopping smoking	current smoker	CPRD GOLD
	137e.00	Smoking restarted	current smoker	CPRD GOLD
	137f.00	Reason for restarting smoking	current smoker	CPRD GOLD
	137h.00	Minutes from waking to first tobacco consumption	current smoker	CPRD GOLD

	T509	SMOKER	current smoker	CPRD GOLD
	T509 SR	SMOKING RESTARTED	current smoker	CPRD GOLD
	T5090OR	SMOKER OWN ROLLED	current smoker	CPRD GOLD
	T5090XC	SMOKER CIGARETTES	current smoker	CPRD GOLD
	T5092	SMOKING ADVISED TO STOP	current smoker	CPRD GOLD
	T5092S	SMOKING WISHES TO STOP	current smoker	CPRD GOLD
	T5092SA	SMOKING WANTS TO STOP	current smoker	CPRD GOLD
	T510	EXCESSIVE SMOKING	current smoker	CPRD GOLD
	T510 HS	HEAVY SMOKER (20-PLUS PER DAY)	current smoker	CPRD GOLD
	T510 SE	SMOKING EXCESSIVE	current smoker	CPRD GOLD
	T510 SH	SMOKER HEAVY (20-PLUS PER DAY)	current smoker	CPRD GOLD
	T511	SMOKER MODERATE (LESS THAN 20 PER DAY)	current smoker	CPRD GOLD
	T5112	SMOKER (20 PER DAY)	current smoker	CPRD GOLD
	T5113	SMOKER (15 PER DAY)	current smoker	CPRD GOLD
	T5114	SMOKER (10 PER DAY)	current smoker	CPRD GOLD
	T5115	SMOKER (LESS THAN 10 PER DAY)	current smoker	CPRD GOLD
	T5115M	SMOKER MILD (5 OR LESS PER DAY)	current smoker	CPRD GOLD
	T5116	SMOKER(OCCASIONAL)	current smoker	CPRD GOLD
	T5117	SMOKER (30 PER DAY)	current smoker	CPRD GOLD
	T512	SMOKER PIPE	current smoker	CPRD GOLD
	T513	SMOKER CIGARS	current smoker	CPRD GOLD
	137..00	Tobacco consumption	current smoker	CPRD GOLD
	137W.00	Chews tobacco	current smoker	CPRD GOLD
	137g.00	Cigarette pack-years	current smoker	CPRD GOLD
	13WK.00	No smokers in the household	non-smoker	CPRD GOLD
	13cA.00	Smokes drugs	current smoker	CPRD GOLD
	13p..00	Smoking cessation milestones	current smoker	CPRD GOLD
	13p0.00	Negotiated date for cessation of smoking	current smoker	CPRD GOLD
	13p1.00	Smoking status at 4 weeks	current smoker	CPRD GOLD
	13p2.00	Smoking status between 4 and 52 weeks	current smoker	CPRD GOLD
	13p3.00	Smoking status at 52 weeks	current smoker	CPRD GOLD
	13p4.00	Smoking free weeks	current smoker	CPRD GOLD
	13p5.00	Smoking cessation programme start date	current smoker	CPRD GOLD
	5020M	SMOKERS' THROAT	current smoker	CPRD GOLD
	5287MK	SMOKERS' MOUTH PATCHES	current smoker	CPRD GOLD
	6791	Health ed. - smoking	current smoker	CPRD GOLD
	67A3.00	Pregnancy smoking advice	current smoker	CPRD GOLD
	67H1.00	Lifestyle advice regarding smoking	current smoker	CPRD GOLD
	745H.00	Smoking cessation therapy	current smoker	CPRD GOLD
	745H000	Nicotine replacement therapy using nicotine patches	current smoker	CPRD GOLD
	745H100	Nicotine replacement therapy using nicotine gum	current smoker	CPRD GOLD
	745H200	Nicotine replacement therapy using nicotine inhalator	current smoker	CPRD GOLD
	745H300	Nicotine replacement therapy using nicotine lozenges	current smoker	CPRD GOLD
	8B2B.00	Nicotine replacement therapy	current smoker	CPRD GOLD
	8B3Y.00	Over the counter nicotine replacement therapy	current smoker	CPRD GOLD

	8B3f.00	Nicotine replacement therapy provided free	current smoker	CPRD GOLD
	8BP3.00	Nicotine replacement therapy provided by community pharmacis	current smoker	CPRD GOLD
	8CAL.00	Smoking cessation advice	current smoker	CPRD GOLD
	8CAg.00	Smoking cessation advice provided by community pharmacist	current smoker	CPRD GOLD
	8H7i.00	Referral to smoking cessation advisor	current smoker	CPRD GOLD
	8HTK.00	Referral to stop-smoking clinic	current smoker	CPRD GOLD
	8I2I.00	Nicotine replacement therapy contraindicated	current smoker	CPRD GOLD
	8I39.00	Nicotine replacement therapy refused	current smoker	CPRD GOLD
	9N2k.00	Seen by smoking cessation advisor	current smoker	CPRD GOLD
	9N4M.00	DNA - Did not attend smoking cessation clinic	current smoker	CPRD GOLD
	9OO..11	Stop smoking clinic admin.	current smoker	CPRD GOLD
	9OO..12	Stop smoking monitoring admin.	current smoker	CPRD GOLD
	9OO1.00	Attends stop smoking monitor.	current smoker	CPRD GOLD
	9OO2.00	Refuses stop smoking monitor	current smoker	CPRD GOLD
	9OO3.00	Stop smoking monitor default	current smoker	CPRD GOLD
	9OO4.00	Stop smoking monitor 1st lettr	current smoker	CPRD GOLD
	9OO5.00	Stop smoking monitor 2nd lettr	current smoker	CPRD GOLD
	9OO6.00	Stop smoking monitor 3rd lettr	current smoker	CPRD GOLD
	9OO7.00	Stop smoking monitor verb.inv.	current smoker	CPRD GOLD
	9OO8.00	Stop smoking monitor phone inv	current smoker	CPRD GOLD
	9OO9.00	Stop smoking monitoring delete	current smoker	CPRD GOLD
	9OOA.00	Stop smoking monitor.chck done	current smoker	CPRD GOLD
	9OOZ.00	Stop smoking monitor admin.NOS	current smoker	CPRD GOLD
	H310100	Smokers' cough	current smoker	CPRD GOLD
	L5091S	SMOKING STARTED	current smoker	CPRD GOLD
	Y060 J1	STOP SMOKING GROUP	current smoker	CPRD GOLD
	Y060 JJ	CLINIC ANTI-SMOKING	current smoker	CPRD GOLD
	Y060 KA	SMOKING CLINIC	current smoker	CPRD GOLD
	Y0601JJ	CLINIC ANTI-SMOKING ATTENDANCE	current smoker	CPRD GOLD
	Y0601KA	SMOKING CLINIC ATTENDANCE	current smoker	CPRD GOLD
	ZG23300	Advice on smoking	current smoker	CPRD GOLD
	ZRaM.00	Motives for smoking scale	current smoker	CPRD GOLD
	ZRh4.00	Reasons for smoking scale	current smoker	CPRD GOLD
	ZRh4.11	RFS - Reasons for smoking scale	current smoker	CPRD GOLD
	137K000	Recently stopped smoking	ex-smoker	CPRD GOLD
	137i.00	Ex-tobacco chewer	ex-smoker	CPRD GOLD
	137j.00	Ex-cigarette smoker	ex-smoker	CPRD GOLD
	137l.00	Ex roll-up cigarette smoker	ex-smoker	CPRD GOLD
	137m.00	Failed attempt to stop smoking	ex-smoker	CPRD GOLD
	137n.00	Total time smoked	current smoker	CPRD GOLD
	137o.00	Waterpipe tobacco consumption	current smoker	CPRD GOLD
	13p7.00	Smoking status at 12 weeks	current smoker	CPRD GOLD
	9ko..00	Current smoker annual review - enhanced services admin	current smoker	CPRD GOLD
	9ko..11	Current smoker annual review	current smoker	CPRD GOLD

Supplementary Table 12: Dementia codes

medcodeid	description	Snomed_ct_code	Source
2386018	Jakob-Creutzfeldt disease	792004	CPRD Aurum
21256010	Presenile dementia	12348006	CPRD Aurum
22408016	Pick's disease	13092008	CPRD Aurum
26545010	Senile dementia	15662003	CPRD Aurum
45046017	Alzheimer's disease	26929004	CPRD Aurum
51470011	Normal pressure hydrocephalus	30753002	CPRD Aurum
149347010	Binswanger's disease	90099008	CPRD Aurum
251625013	H/O: dementia	161465002	CPRD Aurum
294635013	Uncomplicated senile dementia	191449005	CPRD Aurum
294637017	Uncomplicated presenile dementia	191451009	CPRD Aurum
294638010	Presenile dementia with delirium	191452002	CPRD Aurum
294641018	Presenile dementia with paranoia	191454001	CPRD Aurum
294642013	Presenile dementia with depression	191455000	CPRD Aurum
294643015	Presenile dementia NOS	12348006	CPRD Aurum
294644014	Senile dementia with depressive or paranoid features	191457008	CPRD Aurum
294645010	Senile dementia with paranoia	191458003	CPRD Aurum
294646011	Senile dementia with depression	191459006	CPRD Aurum
294647019	Senile dementia with depressive or paranoid features NOS	191457008	CPRD Aurum
294648012	Senile dementia with delirium	191461002	CPRD Aurum
294652012	Uncomplicated arteriosclerotic dementia	191463004	CPRD Aurum
294653019	Arteriosclerotic dementia with delirium	191464005	CPRD Aurum
294654013	Arteriosclerotic dementia with paranoia	191465006	CPRD Aurum
294655014	Arteriosclerotic dementia with depression	191466007	CPRD Aurum
294656010	Arteriosclerotic dementia NOS	56267009	CPRD Aurum
294660013	Senile or presenile psychoses NOS	268612007	CPRD Aurum
294688019	Drug-induced dementia	191493005	CPRD Aurum
294718018	Dementia in conditions EC	191519005	CPRD Aurum
295668011	[X]Dementia in Alzheimer's disease	26929004	CPRD Aurum
295671015	[X]Dementia in Alzheimer's dis, atypical or mixed type	26929004	CPRD Aurum
295672010	[X]Dementia in Alzheimer's disease, unspecified	26929004	CPRD Aurum
295680015	[X]Other vascular dementia	429998004	CPRD Aurum
295681016	[X]Vascular dementia, unspecified	429998004	CPRD Aurum
295684012	[X]Dementia in other diseases classified elsewhere	191519005	CPRD Aurum
295685013	[X]Dementia in Pick's disease	21921000000000	CPRD Aurum
295686014	[X]Dementia in Creutzfeldt-Jakob disease	429458009	CPRD Aurum
295687017	[X]Dementia in Huntington's disease	442344002	CPRD Aurum
295688010	[X]Dementia in Parkinson's disease	425390006	CPRD Aurum
295690011	[X]Dementia in other specified diseases classif elsewhere	191519005	CPRD Aurum
295714013	[X]Delirium superimposed on dementia	2776000	CPRD Aurum
299325013	[X]Other Alzheimer's disease	26929004	CPRD Aurum
345092016	Frontotemporal degeneration	230273006	CPRD Aurum
346929012	Alcoholic dementia NOS	281004	CPRD Aurum
401760017	Other alcoholic dementia	281004	CPRD Aurum
497559016	Arteriosclerotic dementia	56267009	CPRD Aurum

499946014	Alzheimer's disease with early onset	416780008	CPRD Aurum
500317011	Alzheimer's disease with late onset	416975007	CPRD Aurum
1235534016	Binswanger's encephalopathy	90099008	CPRD Aurum
148381000006115	Senile/presenile dementia	52448006	CPRD Aurum
294111000000119	Exception reporting: dementia quality indicators	715881000000000	CPRD Aurum
294321000000111	Excepted from dementia quality indicators: Informed dissent	716131000000000	CPRD Aurum
299641000000112	[X]Lewy body dementia	80098002	CPRD Aurum
359081000006118	[X] Presenile dementia NOS	12348006	CPRD Aurum
359101000006114	[X] Primary degenerative dementia NOS	279982005	CPRD Aurum
359141000006111	[X] Senile dementia NOS	15662003	CPRD Aurum
359151000006113	[X] Senile dementia, depressed or paranoid type	191457008	CPRD Aurum
359241000006119	[X] Unspecified dementia	52448006	CPRD Aurum
362941000006113	[X]Alcoholic dementia NOS	281004	CPRD Aurum
363021000006113	[X]Alzheimer's dementia unspec	26929004	CPRD Aurum
363031000006111	[X]Alzheimer's disease type 1	416975007	CPRD Aurum
363041000006118	[X]Alzheimer's disease type 2	416780008	CPRD Aurum
363791000006112	[X]Arteriosclerotic dementia	56267009	CPRD Aurum
376531000006119	[X]Dementia in Alzheimer's disease with early onset	416780008	CPRD Aurum
376541000006112	[X]Dementia in Alzheimer's disease with late onset	416975007	CPRD Aurum
376571000006116	[X]Dementia in human immunodef virus [HIV] disease	421529006	CPRD Aurum
398571000006112	[X]Mixed cortical and subcortical vascular dementia	230287006	CPRD Aurum
399031000006111	[X]Multi-infarct dementia	56267009	CPRD Aurum
406841000000115	Dementia monitoring administration	713821000000000	CPRD Aurum
407461000000119	Dementia monitoring	248711000000000	CPRD Aurum
408361000000111	Dementia monitoring first letter	715821000000000	CPRD Aurum
408401000000119	Dementia annual review	249181000000000	CPRD Aurum
408421000000111	Dementia monitoring second letter	717471000000000	CPRD Aurum
408461000000115	Dementia monitoring third letter	716671000000000	CPRD Aurum
408481000000112	Dementia monitoring verbal invite	716221000000000	CPRD Aurum
408501000000115	Dementia monitoring telephone invite	716991000000000	CPRD Aurum
423221000006117	[X]Predominantly cortical dementia	56267009	CPRD Aurum
423351000006115	[X]Presenile dementia,Alzheimer's type	416780008	CPRD Aurum
423381000006111	[X]Primary degen dementia of Alzheimer's type, senile onset	416975007	CPRD Aurum
423391000006114	[X]Primary degen dementia, Alzheimer's type, presenile onset	416780008	CPRD Aurum
425901000006116	[X]Senile dementia,Alzheimer's type	416975007	CPRD Aurum
428201000006119	[X]Subcortical vascular dementia	230286002	CPRD Aurum
431681000006117	[X]Vascular dementia	429998004	CPRD Aurum
431691000006119	[X]Vascular dementia of acute onset	230285003	CPRD Aurum
542651000006110	Cerebral degeneration due to Jakob - Creutzfeldt disease	192818008	CPRD Aurum
696161000006115	Multi infarct dementia	56267009	CPRD Aurum
745381000006119	Lewy body disease	80098002	CPRD Aurum
753321000006116	Korsakoff's non-alcoholic psychosis	17262008	CPRD Aurum
882171000006115	Dementia	268612007	CPRD Aurum
882181000006117	Other senile/presenile dement.	268612007	CPRD Aurum
882191000006119	Senile and presenile dementias	268612007	CPRD Aurum
882201000006116	Senile dementia - simple type	191449005	CPRD Aurum

882211000006118	Senile dementia-acute confused	191461002	CPRD Aurum
905791000006115	[RFC] Alzheimer's disease	905791000000000	CPRD Aurum
914921000006117	[D] Vascular dementia	914921000000000	CPRD Aurum
914931000006119	[D] Dementia with Lewy bodies	914931000000000	CPRD Aurum
914941000006112	[D] Dementia	914941000000000	CPRD Aurum
914951000006114	[D] Dementia in Alzheimer's disease	914951000000000	CPRD Aurum
915111000006112	Dementia review	915111000000000	CPRD Aurum
915121000006116	Dementia review with third party	915121000000000	CPRD Aurum
939491000006118	[RFC] Dementia	939491000000000	CPRD Aurum
999961000006114	Excepted from dementia quality indicators: Patient unsuitabl	716341000000000	CPRD Aurum
1774581000006110	Refer to dementia care advisor	1774580000000000	CPRD Aurum
1805751000006110	Variant Creutzfeldt-Jakob disease	304603007	CPRD Aurum
1820331000006110	History of Creutzfeldt-Jakob disease	1820330000000000	CPRD Aurum
1823871000006110	Dementia confirmed	1823870000000000	CPRD Aurum
1834091000006110	Dementia care plan	1834090000000000	CPRD Aurum
1856601000006110	Dementia monitoring in primary care	1856600000000000	CPRD Aurum
1856611000006110	Dementia monitoring in secondary care	1856610000000000	CPRD Aurum
1856631000006110	Did not attend dementia monitoring	1856630000000000	CPRD Aurum
1897891000006110	Sporadic Creutzfeldt-Jakob disease	1897890000000000	CPRD Aurum
1916981000006110	Dementia medication review	1916980000000000	CPRD Aurum
1949611000006110	Dementia post diagnostic support (Scotland)	1949610000000000	CPRD Aurum
1949621000006110	Dementia stage at diagnosis	1949620000000000	CPRD Aurum
1949631000006110	Dementia stage at diagnosis - early (mild)	1949630000000000	CPRD Aurum
1949641000006110	Dementia stage at diagnosis - mid (moderate)	1949640000000000	CPRD Aurum
1949651000006110	Dementia stage at diagnosis - late (severe)	1949650000000000	CPRD Aurum
1949661000006110	Dementia stage at diagnosis - undetermined	1949660000000000	CPRD Aurum
1949671000006110	Dementia stage at diagnosis - not known	1949670000000000	CPRD Aurum
1949681000006110	Person centred dementia support plan (Scotland)	1949680000000000	CPRD Aurum
1949691000006110	Dementia support plan (Scot) in place at 12 months	1949690000000000	CPRD Aurum
1949701000006110	Dementia support plan (Scot) partly in place at 12 months	1949700000000000	CPRD Aurum
1949711000006110	Dementia support plan (Scot) absent at 12 months	1949710000000000	CPRD Aurum
1949721000006110	Dementia post diagnostic support (PDS) - transition arrangements	1949720000000000	CPRD Aurum
1949731000006110	Dementia PDS transition - progress to supported self management	1949730000000000	CPRD Aurum
1949751000006110	Dementia PDS transition - further PDS required	1949750000000000	CPRD Aurum
1949761000006110	Dementia PDS transition - referred for community care support	1949760000000000	CPRD Aurum
1949771000006110	Dementia post diagnostic support (PDS)(Scot)- 5 pillar model	1949770000000000	CPRD Aurum
1950411000006110	Dementia PDS(Scot)-understanding illness/managing symptms status	1950410000000000	CPRD Aurum
1950421000006110	Dementia PDS (Scot) understanding illness status - pillar met	1950420000000000	CPRD Aurum
1950431000006110	Dementia PDS (Scot) understanding illness - pillar partially met	1950430000000000	CPRD Aurum
1950451000006110	Dementia PDS understanding illness-pillar not met(service reasn)	1950450000000000	CPRD Aurum
1950461000006110	Dementia PDS understanding illness - pillar not met (pt choice)	1950460000000000	CPRD Aurum
1950481000006110	Dementia PDS understanding illness - other pillar status	1950480000000000	CPRD Aurum
1950491000006110	Dementia PDS understanding illness - pillar status not known	1950490000000000	CPRD Aurum
1950501000006110	Dementia PDS (Scot) - planning for future care status	1950500000000000	CPRD Aurum
1950511000006110	Dementia PDS (Scot) planning future care status - pillar met	1950510000000000	CPRD Aurum
1950521000006110	Dementia PDS (Scot) planning future care - pillar partially met	1950520000000000	CPRD Aurum

1950531000006110	Dementia PDS planning future care-pillar not met(service reason)	1950530000000000	CPRD Aurum
1950541000006110	Dementia PDS planning future care - pillar not met (pt choice)	1950540000000000	CPRD Aurum
1950551000006110	Dementia PDS planning future care - other pillar status	1950550000000000	CPRD Aurum
1950561000006110	Dementia PDS planning future care - pillar status not known	1950560000000000	CPRD Aurum
1950571000006110	Dementia PDS (Scot) - peer support status	1950570000000000	CPRD Aurum
1950581000006110	Dementia PDS (Scot) peer support status - pillar met	1950580000000000	CPRD Aurum
1950591000006110	Dementia PDS (Scot) peer support status - pillar partially met	1950590000000000	CPRD Aurum
1950601000006110	Dementia PDS (Scot) peer support-pillar not met (service reason)	1950600000000000	CPRD Aurum
1950611000006110	Dementia PDS (Scot) peer support - pillar not met (pt choice)	1950610000000000	CPRD Aurum
1950621000006110	Dementia PDS (Scot) peer support - other pillar status	1950620000000000	CPRD Aurum
1950631000006110	Dementia PDS (Scot) peer support - pillar status not known	1950630000000000	CPRD Aurum
1950641000006110	Dementia PDS (Scot) - supporting community connections status	1950640000000000	CPRD Aurum
1950651000006110	Dementia PDS (Scot) support community connections - pillar met	1950650000000000	CPRD Aurum
1950661000006110	Dementia PDS support community connections -pillar partially met	1950660000000000	CPRD Aurum
1950671000006110	Dementia PDS supprt comm. connectns-pillar not met(servce reasn)	1950670000000000	CPRD Aurum
1950681000006110	Dementia PDS support comm. connectns-pillar not met (pt choice)	1950680000000000	CPRD Aurum
1950691000006110	Dementia PDS support comm. connectns - other pillar status	1950690000000000	CPRD Aurum
1950701000006110	Dementia PDS support comm. connectns - pillar status not known	1950700000000000	CPRD Aurum
1950711000006110	Dementia PDS (Scot) - planning future decision-making status	1950710000000000	CPRD Aurum
1950721000006110	Dementia PDS planning future decision-making - pillar met	1950720000000000	CPRD Aurum
1950731000006110	Dementia PDS planning future decisn-making -pillar partially met	1950730000000000	CPRD Aurum
1950741000006110	Dementia PDS plan.futur decisn-mkng-pillar not met(servce reasn)	1950740000000000	CPRD Aurum
1950751000006110	Dementia PDS plan.future decisn-making-pillar not met(pt choice)	1950750000000000	CPRD Aurum
1950761000006110	Dementia PDS plan future decision-making - other pillar status	1950760000000000	CPRD Aurum
1950771000006110	Dementia PDS plan future decision-making-pillar status not known	1950770000000000	CPRD Aurum
1950781000006110	Dementia link worker details	1950780000000000	CPRD Aurum
1950791000006110	Dementia link worker - mental health CPN	1950790000000000	CPRD Aurum
1950801000006110	No longer has a dementia link worker	1950800000000000	CPRD Aurum
1950811000006110	Dementia link worker - mental health OT	1950810000000000	CPRD Aurum
1950821000006110	Dementia link worker - other mental health worker	1950820000000000	CPRD Aurum
1950841000006110	Dementia link worker - third sector (Alzheimer Scotland)	1950840000000000	CPRD Aurum
1950851000006110	Dementia link worker - other third sector worker	1950850000000000	CPRD Aurum
1950861000006110	Dementia link worker - social worker	1950860000000000	CPRD Aurum
1950871000006110	Dementia link worker - other	1950870000000000	CPRD Aurum
1971401000006110	Dementia in Alzheimer's disease with early onset, without additional symptoms	1971400000000000	CPRD Aurum
1971541000006110	Dementia in Alzheimer's disease with early onset, other symptoms, predominantly delusional	1971540000000000	CPRD Aurum
1971661000006110	Mental & behav dis due to seds/hypntcs: resid & late-onset psychot dis, dementia	1971660000000000	CPRD Aurum
1971701000006110	Mental & behav dis due to use opioids: resid & late-onset psychot dis, dementia	1971700000000000	CPRD Aurum
1971771000006110	Dementia in Alzheimer's disease with early onset, other symptoms, predominantly hallucinatory	1971770000000000	CPRD Aurum
1972021000006110	Unspecified dementia, without additional symptoms	1972020000000000	CPRD Aurum
1972041000006110	Unspecified dementia, other symptoms, predominantly delusional	1972040000000000	CPRD Aurum
1972061000006110	Unspecified dementia, other symptoms, predominantly hallucinatory	1972060000000000	CPRD Aurum
1972071000006110	Unspecified dementia, other symptoms, predominantly depressive	1972070000000000	CPRD Aurum
1972081000006110	Unspecified dementia, other mixed symptoms	1972080000000000	CPRD Aurum

1972131000006110	Dementia in Alzheimer's disease with early onset, other symptoms, predominantly depressive	1972130000000000	CPRD Aurum
1972141000006110	Dementia in Alzheimer's disease with early onset, other mixed symptoms	1972140000000000	CPRD Aurum
1972171000006110	Dementia in Alzheimer's disease with late onset, without additional symptoms	1972170000000000	CPRD Aurum
1972181000006110	Dementia in Alzheimer's disease with late onset, other symptoms, predominantly delusional	1972180000000000	CPRD Aurum
1972191000006110	Dementia in Alzheimer's disease with late onset, other symptoms, predominantly hallucinatory	1972190000000000	CPRD Aurum
1972201000006110	Dementia in Alzheimer's disease with late onset, other symptoms, predominantly depressive	1972200000000000	CPRD Aurum
1972211000006110	Dementia in Alzheimer's disease with late onset, other mixed symptoms	1972210000000000	CPRD Aurum
1972231000006110	Dementia in Alzheimer's dis, atypical or mixed type, without additional symptoms	1972230000000000	CPRD Aurum
1972251000006110	Dementia in Alzheimer's dis, atypical or mixed type, other symptoms, predominantly delusional	1972250000000000	CPRD Aurum
1972291000006110	Dementia in Alzheimer's dis, atypical or mixed type, other symptoms, predominantly hallucinatory	1972290000000000	CPRD Aurum
1972311000006110	Dementia in Alzheimer's dis, atypical or mixed type, other symptoms, predominantly depressive	1972310000000000	CPRD Aurum
1972341000006110	Dementia in Alzheimer's dis, atypical or mixed type, other mixed symptoms	1972340000000000	CPRD Aurum
1972371000006110	Dementia in Alzheimer's disease, unspecified, without additional symptoms	1972370000000000	CPRD Aurum
1972401000006110	Dementia in Alzheimer's disease, unspecified, other symptoms, predominantly delusional	1972400000000000	CPRD Aurum
1972421000006110	Dementia in Alzheimer's disease, unspecified, other symptoms, predominantly hallucinatory	1972420000000000	CPRD Aurum
1972431000006110	Mental & behav dis due to cannabinoids: resid & late-onset psychot dis, dementia	1972430000000000	CPRD Aurum
1972451000006110	Dementia in Alzheimer's disease, unspecified, other symptoms, predominantly depressive	1972450000000000	CPRD Aurum
1972471000006110	Dementia in Alzheimer's disease, unspecified, other mixed symptoms	1972470000000000	CPRD Aurum
1972481000006110	Vascular dementia of acute onset, without additional symptoms	1972480000000000	CPRD Aurum
1972501000006110	Vascular dementia of acute onset, other symptoms, predominantly delusional	1972500000000000	CPRD Aurum
1972521000006110	Vascular dementia of acute onset, other symptoms, predominantly hallucinatory	1972520000000000	CPRD Aurum
1972541000006110	Vascular dementia of acute onset, other symptoms, predominantly depressive	1972540000000000	CPRD Aurum
1972571000006110	Vascular dementia of acute onset, other mixed symptoms	1972570000000000	CPRD Aurum
1972601000006110	Multi-infarct dementia, without additional symptoms	1972600000000000	CPRD Aurum
1972621000006110	Multi-infarct dementia, other symptoms, predominantly delusional	1972620000000000	CPRD Aurum
1972641000006110	Multi-infarct dementia, other symptoms, predominantly hallucinatory	1972640000000000	CPRD Aurum
1972661000006110	Multi-infarct dementia, other symptoms, predominantly depressive	1972660000000000	CPRD Aurum
1972681000006110	Multi-infarct dementia, other mixed symptoms	1972680000000000	CPRD Aurum
1972711000006110	Subcortical vascular dementia, without additional symptoms	1972710000000000	CPRD Aurum
1972731000006110	Subcortical vascular dementia, other symptoms, predominantly delusional	1972730000000000	CPRD Aurum
1972751000006110	Subcortical vascular dementia, other symptoms, predominantly hallucinatory	1972750000000000	CPRD Aurum
1972771000006110	Subcortical vascular dementia, other symptoms, predominantly depressive	1972770000000000	CPRD Aurum
1972791000006110	Subcortical vascular dementia, other mixed symptoms	1972790000000000	CPRD Aurum
1972821000006110	Mixed cortical and subcortical vascular dementia, without additional symptoms	1972820000000000	CPRD Aurum
1972831000006110	Mixed cortical and subcortical vascular dementia, other symptoms, predominantly delusional	1972830000000000	CPRD Aurum
1972871000006110	Mixed cortical and subcortical vascular dementia, other symptoms, predominantly hallucinatory	1972870000000000	CPRD Aurum
1972911000006110	Mixed cortical and subcortical vascular dementia, other symptoms, predominantly depressive	1972910000000000	CPRD Aurum
1972931000006110	Mixed cortical and subcortical vascular dementia, other mixed symptoms	1972930000000000	CPRD Aurum

1973171000006110	Mental and behav dis due to vol solvents: resid & late-onset psychotic dis, dementia	1973170000000000	CPRD Aurum
1973221000006110	Other vascular dementia, without additional symptoms	1973220000000000	CPRD Aurum
1973271000006110	Other vascular dementia, other symptoms, predominantly delusional	1973270000000000	CPRD Aurum
1973341000006110	Other vascular dementia, other symptoms, predominantly hallucinatory	1973340000000000	CPRD Aurum
1973381000006110	Other vascular dementia, other symptoms, predominantly depressive	1973380000000000	CPRD Aurum
1973401000006110	Other vascular dementia, other mixed symptoms	1973400000000000	CPRD Aurum
1973461000006110	Vascular dementia, unspecified, without additional symptoms	1973460000000000	CPRD Aurum
1973501000006110	Vascular dementia, unspecified, other symptoms, predominantly delusional	1973500000000000	CPRD Aurum
1973531000006110	Vascular dementia, unspecified, other symptoms, predominantly hallucinatory	1973530000000000	CPRD Aurum
1973551000006110	Vascular dementia, unspecified, other symptoms, predominantly depressive	1973550000000000	CPRD Aurum
1973711000006110	Mental and behav dis due to hallucinogens: resid & late-onset psychot dis, dementia	1973710000000000	CPRD Aurum
1973941000006110	Mental & behav dis due to use cocaine: resid & late-onset psychot dis, dementia	1973940000000000	CPRD Aurum
1974271000006110	Mental and behav dis mlti drg use/oth psych subs: resid/late psychot dis, dementia	1974270000000000	CPRD Aurum
1974931000006110	Mental & behav dis due to tobacco: resid & late-onset psychot dis, dementia	1974930000000000	CPRD Aurum
1975591000006110	Mental & behav dis due to use alcohol: resid & late-onset psychot dis, dementia	1975590000000000	CPRD Aurum
1976091000006110	Mental and behav dis due to other stimulants inc caffeine: resid/late-onset psycht dis, dementia	1976090000000000	CPRD Aurum
1976831000006110	Vascular dementia, unspecified, other mixed symptoms	1976830000000000	CPRD Aurum
2248021000000110	Dementia care plan	736371006	CPRD Aurum
2290431000000110	Antipsychotic drug therapy for dementia	700214004	CPRD Aurum
2345801000000110	Dementia advance care plan declined	956881000000000	CPRD Aurum
2345881000000110	Dementia advance care plan agreed	1095120000000000	CPRD Aurum
2345931000000110	Review of dementia advance care plan	956861000000000	CPRD Aurum
2366171000000110	Sporadic Creutzfeldt-Jakob disease	713060000	CPRD Aurum
2366181000000110	Sporadic CJD (Creutzfeldt-Jakob disease)	713060000	CPRD Aurum
2403101000000110	Dementia medication review	938551000000000	CPRD Aurum
2439591000000110	Dementia care plan agreed	956841000000000	CPRD Aurum
2439631000000110	Dementia care plan reviewed	956861000000000	CPRD Aurum
2439671000000110	Dementia care plan declined	956881000000000	CPRD Aurum
2439711000000110	Dementia care plan review declined	956901000000000	CPRD Aurum
2445471000000110	Dementia advance care plan	959361000000000	CPRD Aurum
2445691000000110	Dementia advance care plan review declined	959461000000000	CPRD Aurum
2502971000006110	Dementia associated with alcoholism	281004	CPRD Aurum
2502981000006110	Alcohol-induced persisting dementia	281004	CPRD Aurum
2510951000006110	Creutzfeldt-Jakob disease	792004	CPRD Aurum
2510981000006110	CJD - Creutzfeldt-Jakob disease	792004	CPRD Aurum
2510991000006110	JCD - Jakob-Creutzfeldt disease	792004	CPRD Aurum
2511001000006110	Transmissible virus dementia	792004	CPRD Aurum
2511011000006110	Creutzfeldt Jakob disease	792004	CPRD Aurum
2620431000000110	Shared care prescribing of drugs for dementia	719787003	CPRD Aurum
2620471000000110	Shared care prescribing of drugs for dementia declined	720022007	CPRD Aurum
2707661000006110	Pick disease	13092008	CPRD Aurum
2707671000006110	Picks disease	13092008	CPRD Aurum
2714871000000110	Signposting to dementia support service	1083390000000000	CPRD Aurum

2743011000000110	Dementia advance care plan agreed	1095120000000000	CPRD Aurum
2748441000006110	SD - Senile dementia	15662003	CPRD Aurum
2773801000006110	Non-alcoholic Korsakoff's psychosis	17262008	CPRD Aurum
2773841000006110	Non-alcoholic Korsakoff psychosis	17262008	CPRD Aurum
2931231000006110	AD - Alzheimer's disease	26929004	CPRD Aurum
2931241000006110	Alzheimer disease	26929004	CPRD Aurum
2931251000006110	Alzheimer dementia	26929004	CPRD Aurum
2966081000006110	Nuchal dystonia-dementia syndrome	28978003	CPRD Aurum
2995751000006110	NPH - Normal pressure hydrocephalus	30753002	CPRD Aurum
3341641000006110	Dementia paralytica	51928006	CPRD Aurum
3341701000006110	Paralytic dementia	51928006	CPRD Aurum
3350441000006110	Organic dementia	52448006	CPRD Aurum
3414231000006110	MID - Multi-infarct dementia	56267009	CPRD Aurum
3414251000006110	VAD - Vascular dementia	56267009	CPRD Aurum
3414261000006110	Multi infarct dementia	56267009	CPRD Aurum
3630091000006110	Wernicke-Korsakoff syndrome	69482004	CPRD Aurum
3630121000006110	Korsakoff psychosis	69482004	CPRD Aurum
3802621000006110	Lewy body variant of Alzheimer's disease	80098002	CPRD Aurum
3802631000006110	SDLT - Senile dementia of the Lewy body type	80098002	CPRD Aurum
3802641000006110	LBD - Lewy body disease	80098002	CPRD Aurum
3802651000006110	Dementia of the Lewy body type	80098002	CPRD Aurum
3802661000006110	DLBD - Diffuse Lewy body disease	80098002	CPRD Aurum
3802671000006110	Cortical Lewy body disease	80098002	CPRD Aurum
3802681000006110	CLBD - Cortical Lewy body disease	80098002	CPRD Aurum
3964591000006110	Subcortical leucoencephalopathy	90099008	CPRD Aurum
3964601000006110	Subcortical leukoencephalopathy	90099008	CPRD Aurum
3964611000006110	Binswanger's dementia	90099008	CPRD Aurum
3964651000006110	Subcortical arteriosclerotic encephalopathy	90099008	CPRD Aurum
3964661000006110	Subcortical atherosclerotic dementia	90099008	CPRD Aurum
4539871000006110	History of dementia	161465002	CPRD Aurum
4768981000006110	Cerebral degeneration due to Creutzfeldt-Jakob disease	192818008	CPRD Aurum
5931631000006110	nvCJD - New variant of Creutzfeldt-Jakob disease	304603007	CPRD Aurum
5931641000006110	vCJD - variant Creutzfeldt-Jakob disease	304603007	CPRD Aurum
5931651000006110	Creutzfeldt-Jakob variant disease	304603007	CPRD Aurum
6897211000006110	Primary degenerative dementia of the Alzheimer type, presenile onset	416780008	CPRD Aurum
6897221000006110	Primary degenerative dementia of the Alzheimer type, early onset	416780008	CPRD Aurum
6897241000006110	Dementia of the Alzheimers type with early onset	416780008	CPRD Aurum
6897251000006110	Presenile dementia, Alzheimer's type	416780008	CPRD Aurum
6897271000006110	Dementia in Alzheimer's disease - type 2	416780008	CPRD Aurum
6900181000006110	Primary degenerative dementia of the Alzheimer type, senile onset	416975007	CPRD Aurum
6900191000006110	Primary degenerative dementia of the Alzheimer type, late onset	416975007	CPRD Aurum
6900201000006110	Dementia of the Alzheimers type, late onset	416975007	CPRD Aurum
6900221000006110	SDAT - Senile dementia, Alzheimer's type	416975007	CPRD Aurum
6900241000006110	Dementia in Alzheimer's disease - type 1	416975007	CPRD Aurum
6973421000006110	Dementia associated with AIDS	421529006	CPRD Aurum
6973431000006110	Acquired immune deficiency syndrome-related dementia	421529006	CPRD Aurum

6973441000006110	AIDS - Acquired immune deficiency syndrome dementia complex	421529006	CPRD Aurum
6973451000006110	ADC - Acquired immune deficiency syndrome dementia complex	421529006	CPRD Aurum
6973461000006110	Acquired immune deficiency syndrome dementia complex	421529006	CPRD Aurum
6973471000006110	Dementia associated with acquired immunodeficiency syndrome	421529006	CPRD Aurum
7043651000006110	Dementia associated with Parkinson's Disease	425390006	CPRD Aurum
7043661000006110	Dementia associated with Parkinson Disease	425390006	CPRD Aurum
7103601000006110	Dementia due to Creutzfeldt-Jakob disease	429458009	CPRD Aurum
7263011000006110	Dementia due to Huntington disease	442344002	CPRD Aurum
7263021000006110	Dementia due to Huntingtons disease	442344002	CPRD Aurum
7699251000006110	Sporadic Jakob-Creutzfeldt disease	713060000	CPRD Aurum
8009521000006110	Dementia due to Picks disease	21921000000000	CPRD Aurum
8009531000006110	Dementia due to Pick disease	21921000000000	CPRD Aurum
8009541000006110	Dementia co-occurrent and due to Pick's disease	21921000000000	CPRD Aurum
12626321000000000	QOF (Quality and Outcomes Framework) dementia quality indicator-related care invitation	1110900000000000	CPRD Aurum
**medcode**	**description**	**readcode**	**database**
1350	Senile/presenile dementia	E00..12	CPRD GOLD
1916	Senile dementia	E00..11	CPRD GOLD
1917	Alzheimer's disease	F110.00	CPRD GOLD
4357	[X] Senile dementia NOS	Eu02z14	CPRD GOLD
4693	[X] Unspecified dementia	Eu02z00	CPRD GOLD
5095	Binswanger's disease	F21y200	CPRD GOLD
5931	H/O: dementia	1461	CPRD GOLD
6578	[X]Vascular dementia	Eu01.00	CPRD GOLD
7323	Uncomplicated senile dementia	E000.00	CPRD GOLD
7572	Lewy body disease	F116.00	CPRD GOLD
7664	[X]Dementia in Alzheimer's disease	Eu00.00	CPRD GOLD
8195	[X]Alzheimer's dementia unspec	Eu00z11	CPRD GOLD
8634	Multi infarct dementia	E004.11	CPRD GOLD
8934	[X]Subcortical vascular dementia	Eu01200	CPRD GOLD
9509	[X]Dementia in Parkinson's disease	Eu02300	CPRD GOLD
9565	[X]Arteriosclerotic dementia	Eu01.11	CPRD GOLD
10288	Normal pressure hydrocephalus	F113000	CPRD GOLD
11136	Pick's disease	F111.00	CPRD GOLD
11175	[X]Multi-infarct dementia	Eu01100	CPRD GOLD
11379	[X]Senile dementia,Alzheimer's type	Eu00112	CPRD GOLD
12621	[X]Dementia in other diseases classified elsewhere	Eu02.00	CPRD GOLD
12710	Dementia annual review	6AB..00	CPRD GOLD
15165	Presenile dementia	E001.00	CPRD GOLD
16797	Alzheimer's disease with early onset	F110000	CPRD GOLD
18386	Senile dementia with paranoia	E002000	CPRD GOLD
19393	[X]Vascular dementia, unspecified	Eu01z00	CPRD GOLD
19477	Arteriosclerotic dementia	E004.00	CPRD GOLD
21887	Senile dementia with depression	E002100	CPRD GOLD
23835	Korsakoff's non-alcoholic psychosis	E040.11	CPRD GOLD
25386	Dementia in conditions EC	E041.00	CPRD GOLD
25704	[X]Presenile dementia,Alzheimer's type	Eu00011	CPRD GOLD

26270	[X]Lewy body dementia	Eu02500	CPRD GOLD
26323	[X]Alcoholic dementia NOS	Eu10711	CPRD GOLD
27342	Alcoholic dementia NOS	E012.11	CPRD GOLD
27677	Presenile dementia with depression	E001300	CPRD GOLD
27759	[X] Senile dementia, depressed or paranoid type	Eu02z16	CPRD GOLD
28402	[X]Dementia in Pick's disease	Eu02000	CPRD GOLD
29386	[X]Dementia in Alzheimer's disease, unspecified	Eu00z00	CPRD GOLD
30032	Presenile dementia with paranoia	E001200	CPRD GOLD
30641	Excepted from dementia quality indicators: Patient unsuitabl	9hD0.00	CPRD GOLD
30706	[X]Dementia in Alzheimer's dis, atypical or mixed type	Eu00200	CPRD GOLD
31016	[X]Mixed cortical and subcortical vascular dementia	Eu01300	CPRD GOLD
32057	Alzheimer's disease with late onset	F110100	CPRD GOLD
34944	[X] Primary degenerative dementia NOS	Eu02z13	CPRD GOLD
37014	[X]Dementia in Huntington's disease	Eu02200	CPRD GOLD
37015	Senile dementia with delirium	E003.00	CPRD GOLD
38286	Jakob-Creutzfeldt disease	A411.00	CPRD GOLD
38438	Presenile dementia NOS	E001z00	CPRD GOLD
38678	[X]Dementia in Alzheimer's disease with late onset	Eu00100	CPRD GOLD
40805	Excepted from dementia quality indicators: Informed dissent	9hD1.00	CPRD GOLD
41089	Senile dementia with depressive or paranoid features NOS	E002z00	CPRD GOLD
41185	[X]Dementia in human immunodef virus [HIV] disease	Eu02400	CPRD GOLD
42279	Arteriosclerotic dementia NOS	E004z00	CPRD GOLD
42602	Uncomplicated presenile dementia	E001000	CPRD GOLD
43089	Uncomplicated arteriosclerotic dementia	E004000	CPRD GOLD
43292	Arteriosclerotic dementia with depression	E004300	CPRD GOLD
43346	[X]Primary degen dementia of Alzheimer's type, senile onset	Eu00113	CPRD GOLD
44341	Exception reporting: dementia quality indicators	9hD..00	CPRD GOLD
44674	Senile dementia with depressive or paranoid features	E002.00	CPRD GOLD
46488	[X]Vascular dementia of acute onset	Eu01000	CPRD GOLD
46762	[X]Alzheimer's disease type 1	Eu00111	CPRD GOLD
48501	[X] Presenile dementia NOS	Eu02z11	CPRD GOLD
48531	Cerebral degeneration due to Jakob - Creutzfeldt disease	F11x700	CPRD GOLD
49263	[X]Dementia in Alzheimer's disease with early onset	Eu00000	CPRD GOLD
49513	Presenile dementia with delirium	E001100	CPRD GOLD
49674	Dementia monitoring first letter	9Ou1.00	CPRD GOLD
53446	[X]Delirium superimposed on dementia	Eu04100	CPRD GOLD
54106	[X]Dementia in Creutzfeldt-Jakob disease	Eu02100	CPRD GOLD
54505	Other alcoholic dementia	E012.00	CPRD GOLD
55023	Dementia monitoring	66h..00	CPRD GOLD
55222	Language disorder of dementia	ZS7C500	CPRD GOLD
55313	[X]Other vascular dementia	Eu01y00	CPRD GOLD
55467	Arteriosclerotic dementia with paranoia	E004200	CPRD GOLD
55838	[X]Predominantly cortical dementia	Eu01111	CPRD GOLD
56912	Arteriosclerotic dementia with delirium	E004100	CPRD GOLD
59122	[X]Other Alzheimer's disease	Fyu3000	CPRD GOLD
60059	[X]Primary degen dementia, Alzheimer's type, presenile onset	Eu00012	CPRD GOLD

61528	[X]Alzheimer's disease type 2	Eu00013	CPRD GOLD
62132	Drug-induced dementia	E02y100	CPRD GOLD
64267	[X]Dementia in other specified diseases classif elsewhere	Eu02y00	CPRD GOLD
65235	Dementia monitoring telephone invite	9Ou5.00	CPRD GOLD
68194	Binswanger's encephalopathy	F21y211	CPRD GOLD
83576	Dementia monitoring second letter	9Ou2.00	CPRD GOLD
85853	Dementia monitoring administration	9Ou..00	CPRD GOLD
89036	Dementia monitoring third letter	9Ou3.00	CPRD GOLD
89037	Dementia monitoring verbal invite	9Ou4.00	CPRD GOLD
104534	Frontotemporal degeneration	F118.00	CPRD GOLD
106311	Dementia care plan	8CMZ.00	CPRD GOLD
108228	Dementia advance care plan agreed	8CSA.00	CPRD GOLD
108268	Review of dementia advance care plan	8CMG200	CPRD GOLD
108391	Dementia advance care plan declined	8IAe000	CPRD GOLD
109047	Antipsychotic drug therapy for dementia	8BPa.00	CPRD GOLD
109288	Sporadic Creutzfeldt-Jakob disease	A411000	CPRD GOLD
109708	Dementia care plan reviewed	8CMZ100	CPRD GOLD
109731	Dementia care plan agreed	8CMZ000	CPRD GOLD
109737	Dementia medication review	8BM0200	CPRD GOLD
109786	Dementia care plan declined	8CMZ200	CPRD GOLD
109790	Dementia advance care plan	8CMe000	CPRD GOLD
110075	Dementia advance care plan review declined	8IAe200	CPRD GOLD
110123	Dementia care plan review declined	8CMZ300	CPRD GOLD

Supplementary Table 13: Learning disability codes

medcodeid	description	Snomed_ct_code	Database
13594019	Thought retardation	7627009	CPRD Aurum
413177014	Educationally subnormal	276854003	CPRD Aurum
2474674015	Learning disabilities health assessment	413126003	CPRD Aurum
2474708013	Learning disabilities health action plan declined	413162002	CPRD Aurum
2474709017	Learning disabilities health action plan reviewed	413163007	CPRD Aurum
2548475019	On learning disability register	416075005	CPRD Aurum
882781000006116	Moderate mental retardation	61152003	CPRD Aurum
882791000006118	Severe mental retardation	40700009	CPRD Aurum
906941000006119	[RFC] Learning disabilities	906941000006103	CPRD Aurum
1009521000006110	Cause of learning disabilities	1009521000006100	CPRD Aurum
1009531000006110	Cause of learning disabilities: Down's syndrome	1009531000006100	CPRD Aurum
1009541000006110	Cause of learning disabilities: Tuberous sclerosis	1009541000006100	CPRD Aurum
1009561000006110	Cause of learning disabilities: Meningitis/encephalitis	1009561000006100	CPRD Aurum
1009571000006110	Cause of learning disabilities: Fragile X syndrome	1009571000006100	CPRD Aurum
1009591000006110	Cause of learning disabilities: Brain tumour	1009591000006100	CPRD Aurum
1009601000006110	Cause of learning disabilities: Congenital hydrocephalus	1009601000006100	CPRD Aurum
1009631000006110	Cause of learning disabilities: Prader-Willi syndrome	1009631000006100	CPRD Aurum
1009671000006110	Cause of learning disablts: Unknown/awaiting investigation	1009671000006100	CPRD Aurum
1009691000006110	Cause of learning disabilities: Other	1009691000006100	CPRD Aurum
1142901000000110	Learning disabilities annual health assessment declined	514021000000103	CPRD Aurum
1162851000000110	Learning disability annual health check invitation	520801000000100	CPRD Aurum
1563991000006110	Learning disability - specialty	1563991000006100	CPRD Aurum
1620441000006110	Learning disability	1620441000006100	CPRD Aurum
1823961000006110	Learning disability confirmed	1823961000006100	CPRD Aurum
1856971000006110	Did not attend learning disability monitoring	1856971000006100	CPRD Aurum
2379351000000110	Died in learning disability unit	713050009	CPRD Aurum
2008481000006110	Learning disabilities: reasonable adjustments	2008481000006100	CPRD Aurum
329968011	Cretinism	217710005	CPRD Aurum
1775531012	Hypogonadism, diabetes mellitus, alopecia, mental retardation and electrocardiographic abnormalities	237616002	CPRD Aurum
2534201018	Learning disabilities health action plan completed	712491005	CPRD Aurum
395071000006112	[X]Learning disorder NOS	1855002	CPRD Aurum
398241000006118	[X]Mental retardation with autistic features	231536004	CPRD Aurum
398411000006116	[X]Mild mental subnormality	86765009	CPRD Aurum
1129781000000110	[X]Severe learning disability	508171000000105	CPRD Aurum
1129811000000110	[X]Moderate learning disability	984671000000103	CPRD Aurum
1550051000000110	[X]Profound learning disability	984681000000101	CPRD Aurum
600791000006112	Cretinism	217710005	CPRD Aurum
151009017	Mental retardation	110359009	CPRD Aurum
295661017	Other specified mental retardation	110359009	CPRD Aurum
296586012	[X]Other mental retardation	110359009	CPRD Aurum
296592018	[X]Other mental retardation, other impairments of behaviour	110359009	CPRD Aurum
401902015	[X]Unspecified mental retardation	110359009	CPRD Aurum
398201000006115	[X]Mental deficiency NOS	110359009	CPRD Aurum
398231000006111	[X]Mental retardation	110359009	CPRD Aurum

398251000006116	[X]Mental subnormality NOS	110359009	CPRD Aurum
404851000006117	[X]Oth mental retard sig impairment behav req attent/treatmt	110359009	CPRD Aurum
404861000006115	[X]Oth mental retard with statement no or min impairm behav	110359009	CPRD Aurum
430061000006113	[X]Unsp mental retard with statement no or min impairm behav	110359009	CPRD Aurum
430071000006118	[X]Unsp mental retardation without mention impairment behav	110359009	CPRD Aurum
431231000006115	[X]Unspecified mental retardatn, other impairments of behav	110359009	CPRD Aurum
395051000006119	[X]Learn acquisition disab NOS	110359009	CPRD Aurum
3910931000006110	Mild learning disability, intelligence quotient in range 50-70	86765009	CPRD Aurum
3003351000006110	Profound learning impairment, intelligence quotient less than 20	31216003	CPRD Aurum
3493651000006110	Moderate mental retardation (Intelligence Quotient 35-49)	61152003	CPRD Aurum
2621261000006110	Retardation of thought, function	7627009	CPRD Aurum
5108961000006110	Endemic cretinism - neurological type	237566004	CPRD Aurum
3003321000006110	Profound learning disability with intelligence quotient less than 20	31216003	CPRD Aurum
3003331000006110	Profound learning impairment with intelligence quotient less than 20	31216003	CPRD Aurum
3155151000006110	Severe learning disability	40700009	CPRD Aurum
3493661000006110	Moderate learning disability, intelligence quotient in range 35-49	61152003	CPRD Aurum
5108941000006110	Endemic cretinism - mixed type	237565000	CPRD Aurum
7567011000006110	Lubs X-linked mental retardation syndrome	702816000	CPRD Aurum
7577251000006110	Microcephaly, mental retardation and distinct features, with or without Hirschsprung disease	703535000	CPRD Aurum
12181621000000000	Severe intellectual development disorder	40700009	CPRD Aurum
4976471000006110	Intellectual disability	228156007	CPRD Aurum
11998101000000000	Intellectual developmental disorder	110359009	CPRD Aurum
3910941000006110	Mild learning impairment, intelligence quotient in range 50-70	86765009	CPRD Aurum
3720391000006110	Endemic cretinism - hypothyroid	75065003	CPRD Aurum
8191411000006110	[X]Severe learning disability	508171000000105	CPRD Aurum
2528331000006110	Learning disorder	1855002	CPRD Aurum
3155201000006110	Severe learning impairment, intelligence quotient in range 20-34	40700009	CPRD Aurum
8337591000006110	Significant intellectual disability	931001000000105	CPRD Aurum
3493671000006110	Moderate learning impairment, intelligence quotient in range 35-49	61152003	CPRD Aurum
12177541000000000	Profound intellectual development disorder	31216003	CPRD Aurum
2621241000006110	Retardation of thought	7627009	CPRD Aurum
12337641000000000	X-linked intellectual disability with marfanoid habitus	422437002	CPRD Aurum
7577261000006110	Hirschsprung disease-mental retardation syndrome	703535000	CPRD Aurum
3493681000006110	Moderate learning disability	61152003	CPRD Aurum
5591451000006110	ESN - Educationally subnormal	276854003	CPRD Aurum
3910921000006110	Mild mental retardation (Intelligence Quotient 50-70)	86765009	CPRD Aurum
3003311000006110	Profound mental retardation (Intelligence Quotient below 20)	31216003	CPRD Aurum
6989771000006110	X-linked mental retardation with marfanoid habitus syndrome	422437002	CPRD Aurum
12487761000000000	Mental retardation	91138005	CPRD Aurum
12703941000000000	Severe mental retardation (I.Q. 20-34)	40700009	CPRD Aurum
3980931000006110	MR - Mental retardation	91138005	CPRD Aurum
12703921000000000	Profound mental retardation (I.Q. below 20)	31216003	CPRD Aurum
12703791000000000	Moderate mental retardation (I.Q. 35-49)	61152003	CPRD Aurum
12728531000000000	Other specified mental retardation NOS	686511000000102	CPRD Aurum
146051000006113	Severe mental retardation, IQ in range 20-34	40700009	CPRD Aurum

201751000006110	Profound mental retardation with IQ less than 20	31216003	CPRD Aurum
398391000006116	[X]Mild mental retardation without mention impairment behav	86765009	CPRD Aurum
398781000006115	[X]Mod mental retardation without mention impairment behav	61152003	CPRD Aurum
398821000006114	[X]Moderate mental subnormality	61152003	CPRD Aurum
417681000006116	[X]Overactive disorder assoc mental retard/stereotype movts	35919005	CPRD Aurum
423501000006112	[X]Profound mental retardation	31216003	CPRD Aurum
423521000006119	[X]Profound mental subnormality	31216003	CPRD Aurum
426541000006119	[X]Sev mental retardation without mention impairment behav	40700009	CPRD Aurum
426591000006111	[X]Severe mental retardation	40700009	CPRD Aurum
426611000006117	[X]Severe mental subnormality	40700009	CPRD Aurum
785941000006115	Imbecile	61152003	CPRD Aurum
296557014	[X]Mild mental retardation, other impairments of behaviour	86765009	CPRD Aurum
296565012	[X]Mod retard oth behav impair	61152003	CPRD Aurum
296574014	[X]Severe mental retardation, other impairments of behaviour	40700009	CPRD Aurum
398661000006116	[X]Mld mental retard with statement no or min impairm behav	86765009	CPRD Aurum
423481000006119	[X]Profound ment retard sig impairmnt behav req attent/treat	31216003	CPRD Aurum
12190771000000000	Moderate intellectual disability	61152003	CPRD Aurum
12202441000000000	Mild intellectual disability	86765009	CPRD Aurum
882801000006117	Mental subnormality NOS	686511000000102	CPRD Aurum
988941000006119	Mental subnormality NOS	91138005	CPRD Aurum
398761000006113	[X]Mod mental retard sig impairment behav req attent/treatmt	61152003	CPRD Aurum
398771000006118	[X]Mod mental retard with statement no or min impairm behav	61152003	CPRD Aurum
426521000006114	[X]Sev mental retard sig impairment behav req attent/treatmt	40700009	CPRD Aurum
426531000006112	[X]Sev mental retard with statement no or min impairm behav	40700009	CPRD Aurum
882761000006114	Mental subnormality	91138005	CPRD Aurum
398811000006118	[X]Moderate mental retardation	61152003	CPRD Aurum
700071000006118	Moderate mental retardation, IQ in range 35-49	61152003	CPRD Aurum
1821361000006110	Intellectual functioning disability	228156007	CPRD Aurum
1855811000006110	Learning disability monitoring in primary care	1855811000006100	CPRD Aurum
1755021000006110	Learning disability annual health check invitation second letter	712801000000108	CPRD Aurum
1009651000006110	Cause of learning disabilities: Rett syndrome	1009651000006100	CPRD Aurum
923881000006119	General development delay	923881000006103	CPRD Aurum
342177013	Intellectual functioning disability	228156007	CPRD Aurum
376951000006119	[X]Developmental Gerstmann's syndrome	229676007	CPRD Aurum
395061000006117	[X]Learning disability NOS	1855002	CPRD Aurum
398381000006119	[X]Mild mental retardation	86765009	CPRD Aurum
1175631000000110	Exception reporting: learning disability quality indicators	717271000000102	CPRD Aurum
1741521000006110	Did not attend learning disabilities annual health assessmnt	514041000000105	CPRD Aurum
295662012	Other specified mental retardation NOS	110359009	CPRD Aurum
12359591000000000	Hirschsprung disease-intellectual disability syndrome	703535000	CPRD Aurum
3003361000006110	Profound learning disability	31216003	CPRD Aurum
12704331000000000	Endemic cretinism	75065003	CPRD Aurum
302211000000112	Learning disabilities annual health assessment	199751000000100	CPRD Aurum
12703781000000000	Mild mental retardation (I.Q. 50-70)	86765009	CPRD Aurum
12487771000000000	Mental retardation NOS	91138005	CPRD Aurum
2435151000000110	Intellectual development disorder of unknown aetiology	954731000000103	CPRD Aurum

2249771000000110	Under care of community learning disability team	870651000000103	CPRD Aurum
1855831000006110	Learning disability monitoring in secondary care	1855831000006100	CPRD Aurum
1856981000006110	Learning disability follow-up	1856981000006100	CPRD Aurum
1667721000000110	[X]Global developmental delay	224958001	CPRD Aurum
1162931000000110	Learning disability annual health check telephone invitation	520841000000102	CPRD Aurum
1009621000006110	Cause of learning disabilities: Phenylketonuria	1009621000006100	CPRD Aurum
882771000006119	Mild mental retardation	86765009	CPRD Aurum
189611000000113	Learning disabilities health action plan offered	112861000000101	CPRD Aurum
398651000006118	[X]Mld mental retard sig impairment behav req attent/treatmt	86765009	CPRD Aurum
423491000006116	[X]Profound ment retrd wth statement no or min impairm behav	31216003	CPRD Aurum
12181611000000000	Severe intellectual disability	40700009	CPRD Aurum
12177531000000000	Profound intellectual disability	31216003	CPRD Aurum
295664013	Mental retardation NOS	110359009	CPRD Aurum
9881000006115	Other specified mental retardation	110359009	CPRD Aurum
411791000006118	[X]Other mental retardation without mention impairment behav	110359009	CPRD Aurum
430081000006115	[X]Unsp mentl retard sig impairment behav req attent/treatmt	110359009	CPRD Aurum
8337571000006110	Significant intellectual development disorder	931001000000105	CPRD Aurum
12321891000000000	Intellectual disability, congenital heart disease, blepharophimosis, blepharoptosis and hypoplastic teeth	412787009	CPRD Aurum
12359021000000000	Lubs X-linked intellectual disability syndrome	702816000	CPRD Aurum
3910951000006110	Mild learning disability	86765009	CPRD Aurum
12202451000000000	Mild intellectual development disorder	86765009	CPRD Aurum
1755031000006110	Learning disability annual health check invitation third letter	712821000000104	CPRD Aurum
1162891000000110	Learning disability annual health check verbal invitation	520821000000109	CPRD Aurum
1164251000000110	Learning disability annual health check letter invitation	521421000000100	CPRD Aurum
1177021000000110	Learning disabilities annual health check declined	514021000000103	CPRD Aurum
1177051000000110	Did not attend learning disabilities annual health check	514041000000105	CPRD Aurum
1009551000006110	Cause of learning disabilities: Birth trauma	1009551000006100	CPRD Aurum
1009581000006110	Cause of learning disabilities: Late effect of head injury	1009581000006100	CPRD Aurum
1009611000006110	Cause of learning disabilities: Microcephaly	1009611000006100	CPRD Aurum
1009641000006110	Cause of learning disabilities: Smith-Magenis syndrome	1009641000006100	CPRD Aurum
1009661000006110	Cause of learning disabilities: Congenital rubella	1009661000006100	CPRD Aurum
1009681000006110	Cause of learning disabilities: Unknown/despite investigation	1009681000006100	CPRD Aurum
1009801000006110	Level of support for person with adult learning disablts. (ALD)	1009801000006100	CPRD Aurum
908941000006118	[RFC] Learning disability	908941000006102	CPRD Aurum
189601000000111	Learning disabilities administration status	112851000000104	CPRD Aurum
507246016	Mild mental retardation, IQ in range 50-70	86765009	CPRD Aurum
415431010	Goitrous cretin	278503003	CPRD Aurum
12008041000000000	Intellectual development disorder screening	763625008	CPRD Aurum
1222495016	[D]Global retardation	224958001	CPRD Aurum
8337561000006110	Significant learning disability	931001000000105	CPRD Aurum
1550041000000110	[X]Mild learning disability	984661000000105	CPRD Aurum
1755011000006110	Learning disability annual health check invtation 1st letter	712781000000107	CPRD Aurum
12190781000000000	Moderate intellectual development disorder	61152003	CPRD Aurum
2528351000006110	General learning disability	1855002	CPRD Aurum
3003341000006110	Profound learning disability, intelligence quotient less than 20	31216003	CPRD Aurum
3155181000006110	Severe mental retardation (Intelligence Quotient 20-34)	40700009	CPRD Aurum

3155191000006110	Severe learning disability, intelligence quotient in range 20-34	40700009	CPRD Aurum
423361000006118	[X]Prfnd mental retardation without mention impairment behav	31216003	CPRD Aurum
423511000006110	[X]Profound mental retardation, other impairments of behavr	31216003	CPRD Aurum
2730411000000110	Mild intellectual development disorder with significant impairment of behaviour	1089841000000100	CPRD Aurum
5095461000006110	Prune belly syndrome with pulmonic stenosis, mental retardation and deafness	236529001	CPRD Aurum
2452421000000110	Learning disability care plan	962361000000105	CPRD Aurum
8459511000006110	Learning disability annual health check invitation email	1083091000000100	CPRD Aurum
8195041000006110	Cause of learning disability	518831000000103	CPRD Aurum
6762801000006110	Learning disability	408468001	CPRD Aurum
2740451000000110	Mild mental retardation with impairment of behaviour	1093991000000100	CPRD Aurum
8459481000006110	Learning disability annual health check invitation SMS (short message service) text message	1083061000000100	CPRD Aurum
3505252012	Alpha-thalassaemia intellectual disability syndrome linked to chromosome 16	734349003	CPRD Aurum
2730191000000110	Profound intellectual development disorder with impairment of behaviour	1089731000000100	CPRD Aurum
2730211000000110	Severe intellectual development disorder without significant impairment of behaviour	1089741000000100	CPRD Aurum
2730271000000110	Severe intellectual development disorder with impairment of behaviour	1089771000000100	CPRD Aurum
2730291000000110	Moderate intellectual development disorder without significant impairment of behaviour	1089781000000100	CPRD Aurum
2730311000000110	Moderate intellectual development disorder with significant impairment of behaviour	1089791000000100	CPRD Aurum
2730351000000110	Moderate intellectual development disorder with minimal impairment of behaviour	1089811000000100	CPRD Aurum
2730391000000110	Mild intellectual development disorder without significant impairment of behaviour	1089831000000100	CPRD Aurum
2730431000000110	Mild intellectual development disorder with minimal impairment of behaviour	1089851000000100	CPRD Aurum
2740341000000110	Severe mental retardation without significant impairment of behaviour	1089741000000100	CPRD Aurum
2740461000000110	Mild intellectual development disorder with impairment of behaviour	1093991000000100	CPRD Aurum
2740521000000110	Intellectual development disorder with significant impairment of behaviour	1094011000000100	CPRD Aurum
2740551000000110	Intellectual development disorder with minimal impairment of behaviour	1094021000000100	CPRD Aurum
2740581000000110	Intellectual development disorder with impairment of behaviour	1094031000000100	CPRD Aurum
3267011000006110	Mental subnormality	47437004	CPRD Aurum
7282231000006110	Nonverbal learning disorder	443735008	CPRD Aurum
7750931000006110	FRAXE intellectual disability syndrome	716709002	CPRD Aurum
7758091000006110	X-linked epilepsy with learning disability and behaviour disorder syndrome	717223008	CPRD Aurum
7780351000006110	X-linked intellectual disability Schimke type	719010001	CPRD Aurum
7782551000006110	Syndromic X-linked intellectual disability type 7	719160009	CPRD Aurum
7810331000006110	Alport syndrome, intellectual disability, midface hypoplasia, elliptocytosis syndrome	720982007	CPRD Aurum
7828621000006110	Spastic paraplegia, intellectual disability, palmoplantar hyperkeratosis syndrome	722209002	CPRD Aurum
7832001000006110	Male hypergonadotropic hypogonadism, intellectual disability, skeletal anomaly syndrome	722459008	CPRD Aurum
12009571000000000	Intellectual disability, brachydactyly, Pierre Robin syndrome	763744009	CPRD Aurum
12022541000000000	Intellectual disability Birk-Barel type	764861005	CPRD Aurum
12077271000000000	PPP2R5D-related intellectual disability	768677000	CPRD Aurum
12178711000000000	Hyperphosphatasaemia with intellectual disability	33982008	CPRD Aurum
13522691000000000	Intellectual disability with strabismus syndrome	773405004	CPRD Aurum
13530051000000000	AHDC1-related intellectual disability, obstructive sleep apnoea, mild dysmorphism syndrome	774068004	CPRD Aurum

13622001000000000	Severe intellectual disability, progressive spastic diplegia syndrome	782723007	CPRD Aurum
13622051000000000	Intellectual disability, facial dysmorphism syndrome due to SETD5 haploinsufficiency	782736007	CPRD Aurum
13622561000000000	Congenital muscular dystrophy with intellectual disability and severe epilepsy	782772000	CPRD Aurum
13632231000000000	White matter hypoplasia, corpus callosum agenesis, intellectual disability syndrome	783703004	CPRD Aurum
13697811000000000	Significant learning disability	1239331000000100	CPRD Aurum
13784441000000000	Learning disability	110359009	CPRD Aurum
13963471000000000	Autism spectrum disorder with disorder of intellectual development and impaired functional language without loss of previously acquired skills	870267006	CPRD Aurum
13964071000000000	Autism spectrum disorder with disorder of intellectual development and impaired functional language	870305003	CPRD Aurum
14259191000000000	Intellectual disability care plan	962361000000105	CPRD Aurum
**medcode**	**description**	**readcode**	**database**
302	Moderate mental retardation, IQ in range 35-49	E310.00	CPRD GOLD
1278	Imbecile	E310.11	CPRD GOLD
1362	Mental retardation	E3...00	CPRD GOLD
1680	Educationally subnormal	E30..11	CPRD GOLD
1787	Mild mental retardation, IQ in range 50-70	E30..00	CPRD GOLD
4477	[X]Learning disability NOS	Eu81z11	CPRD GOLD
4825	Severe mental retardation, IQ in range 20-34	E311.00	CPRD GOLD
6123	[X]Moderate mental retardation	Eu71.00	CPRD GOLD
11866	Intellectual functioning disability	Z7CBE00	CPRD GOLD
16855	[X]Learning disorder NOS	Eu81z12	CPRD GOLD
19436	Learning disability	ZS34.11	CPRD GOLD
19445	Learning disabilities administration status	9HB..00	CPRD GOLD
22645	Thought retardation	1Ba2.00	CPRD GOLD
22760	On learning disability register	918e.00	CPRD GOLD
27533	Cretinism	C03z.12	CPRD GOLD
27691	[X]Severe mental subnormality	Eu72.11	CPRD GOLD
28740	[X]Mild mental retardation	Eu70.00	CPRD GOLD
28962	[X]Mental retardation	Eu7..00	CPRD GOLD
30362	[D]Global retardation	R034y11	CPRD GOLD
32511	Learning disabilities health assessment	9HB3.00	CPRD GOLD
32820	[X]Unsp mental retardation without mention impairment behav	Eu7zz00	CPRD GOLD
32952	Learning disabilities annual health assessment	9HB5.00	CPRD GOLD
33949	[X]Mild mental subnormality	Eu70.12	CPRD GOLD
34174	[X]Mental retardation with autistic features	Eu84112	CPRD GOLD
34734	[X]Moderate mental subnormality	Eu71.11	CPRD GOLD
36045	[X]Learn acquisition disab NOS	Eu81z13	CPRD GOLD
36143	[X]Severe mental retardation	Eu72.00	CPRD GOLD
37867	Mental retardation NOS	E3z..00	CPRD GOLD
37887	[X]Mental deficiency NOS	Eu7z.11	CPRD GOLD
37911	[X]Mental subnormality NOS	Eu7z.12	CPRD GOLD
39016	[X]Mild mental retardation, other impairments of behaviour	Eu70y00	CPRD GOLD
39412	[X]Mld mental retard sig impairment behav req attent/treatmt	Eu70100	CPRD GOLD
41391	Learning disabilities health action plan offered	9HB1.00	CPRD GOLD
42520	[X]Other mental retardation, other impairments of behaviour	Eu7yy00	CPRD GOLD

42589	[X]Unspecified mental retardation	Eu7z.00	CPRD GOLD
42886	[X]Unsp mental retard with statement no or min impairm behav	Eu7z000	CPRD GOLD
43436	Learning disabilities health action plan completed	9HB4.00	CPRD GOLD
43445	Learning disabilities health action plan reviewed	9HB2.00	CPRD GOLD
43447	Learning disabilities health action plan declined	9HB0.00	CPRD GOLD
45133	Profound mental retardation with IQ less than 20	E312.00	CPRD GOLD
46504	[X]Mld mental retard with statement no or min impairm behav	Eu70000	CPRD GOLD
47449	Goitrous cretin	C031.00	CPRD GOLD
50606	[X]Mild mental retardation without mention impairment behav	Eu70z00	CPRD GOLD
50751	[X]Sev mental retard sig impairment behav req attent/treatmt	Eu72100	CPRD GOLD
50947	[X]Sev mental retard with statement no or min impairm behav	Eu72000	CPRD GOLD
51268	[X]Profound mental retardation	Eu73.00	CPRD GOLD
52602	[X]Overactive disorder assoc mental retard/stereotype movts	Eu84400	CPRD GOLD
54179	Other specified mental retardation NOS	E31z.00	CPRD GOLD
54881	[X]Mod mental retard sig impairment behav req attent/treatmt	Eu71100	CPRD GOLD
55560	[X]Sev mental retardation without mention impairment behav	Eu72z00	CPRD GOLD
55848	[X]Severe mental retardation, other impairments of behaviour	Eu72y00	CPRD GOLD
56547	[X]Oth mental retard sig impairment behav req attent/treatmt	Eu7y100	CPRD GOLD
56577	Other specified mental retardation	E31..00	CPRD GOLD
57162	[X]Developmental Gerstmann's syndrome	Eu81213	CPRD GOLD
57199	Other specified mental retardation	E3y..00	CPRD GOLD
59407	[X]Mod retard oth behav impair	Eu71y00	CPRD GOLD
60062	[X]Prfnd mental retardation without mention impairment behav	Eu73z00	CPRD GOLD
60473	[X]Mod mental retardation without mention impairment behav	Eu71z00	CPRD GOLD
60913	[X]Mod mental retard with statement no or min impairm behav	Eu71000	CPRD GOLD
63273	[X]Other mental retardation without mention impairment behav	Eu7yz00	CPRD GOLD
65468	[X]Profound mental subnormality	Eu73.11	CPRD GOLD
66383	[X]Unspecified mental retardatn, other impairments of behav	Eu7zy00	CPRD GOLD
66783	[X]Unsp mentl retard sig impairment behav req attent/treatmt	Eu7z100	CPRD GOLD
67513	Cretinism	C03..11	CPRD GOLD
70008	[X]Oth mental retard with statement no or min impairm behav	Eu7y000	CPRD GOLD
70102	[X]Profound ment retrd wth statement no or min impairm behav	Eu73000	CPRD GOLD
71196	[X]Other mental retardation	Eu7y.00	CPRD GOLD
90276	[X]Profound mental retardation, other impairments of behavr	Eu73y00	CPRD GOLD
98100	[X]Profound ment retard sig impairmnt behav req attent/treat	Eu73100	CPRD GOLD
98293	[X]Severe learning disability	Eu81500	CPRD GOLD
98342	[X]Moderate learning disability	Eu81400	CPRD GOLD
99774	[X]Mild learning disability	Eu81600	CPRD GOLD
100648	[X]Profound learning disability	Eu81700	CPRD GOLD
100729	Did not attend learning disabilities annual health assessmnt	9HB7.00	CPRD GOLD
100730	Learning disabilities annual health assessment declined	9HB6.00	CPRD GOLD
100899	[X]Global developmental delay	Eu85.00	CPRD GOLD
100965	Learning disabilities annual health check declined	9HB6.11	CPRD GOLD
100980	Exception reporting: learning disability quality indicators	9hL..00	CPRD GOLD
102234	Did not attend learning disabilities annual health check	9HB7.11	CPRD GOLD
106219	Learning disability annual health check invitation	9mA..00	CPRD GOLD

106247	Learning disability annual health check letter invitation	9mA2.00	CPRD GOLD
106248	Learning disability annual health check telephone invitation	9mA1.00	CPRD GOLD
106249	Learning disability annual health check verbal invitation	9mA0.00	CPRD GOLD
106272	Learning disability annual health check invtation 1st letter	9mA2000	CPRD GOLD
106274	Learning disability annual health check invtation 2nd letter	9mA2100	CPRD GOLD
106276	Learning disability annual health check invtation 3rd letter	9mA2200	CPRD GOLD
110427	Intellectual development disorder of unknown aetiology	13VC900	CPRD GOLD
110792	Under care of community learning disability team	9Nh4.00	CPRD GOLD

Supplementary Table 14: Stroke codes

medcodeid	description	Snomed_ct_code	Database
265689012	Evacuation of haematoma from temporal lobe of brain	171473007	CPRD Aurum
265690015	Evacuation of haematoma from cerebellum	171474001	CPRD Aurum
265692011	Evacuation of intracerebral haematoma NEC	10458001	CPRD Aurum
300242011	Ruptured berry aneurysm	195154000	CPRD Aurum
300244012	Subarachnoid haemorrhage from carotid siphon and bifurcation	195155004	CPRD Aurum
300253017	Subarachnoid haemorrhage from vertebral artery	195160000	CPRD Aurum
300257016	Subarachnoid haemorrhage NOS	21454007	CPRD Aurum
300276019	External capsule haemorrhage	195167002	CPRD Aurum
300277011	Intracerebral haemorrhage, intraventricular	195168007	CPRD Aurum
300287010	Intracerebral haemorrhage NOS	274100004	CPRD Aurum
300366019	Cerebellar stroke syndrome	195213000	CPRD Aurum
300406018	Sequelae of subarachnoid haemorrhage	195240000	CPRD Aurum
300407010	Sequelae of intracerebral haemorrhage	195241001	CPRD Aurum
300935019	[X]Subarachnoid haemorrhage from other intracranial arteries	21454007	CPRD Aurum
300936018	[X]Other subarachnoid haemorrhage	21454007	CPRD Aurum
300939013	[X]Other intracerebral haemorrhage	274100004	CPRD Aurum
300956017	[X]Intracerebral haemorrhage in hemisphere, unspecified	274100004	CPRD Aurum
345675012	Lobar cerebral haemorrhage	230710000	CPRD Aurum
359985011	Intracerebral haemorrhage in fetus or newborn	240313004	CPRD Aurum
405339016	Stroke and cerebrovascular accident unspecified	230690007	CPRD Aurum
449935012	Admission to stroke unit	306803007	CPRD Aurum
451371010	H/O: Stroke in last year	308067002	CPRD Aurum
481028017	Subarachnoid haemorrhage	21454007	CPRD Aurum
483988011	Bulbar haemorrhage	732923001	CPRD Aurum
495394013	Cortical haemorrhage	49422009	CPRD Aurum
496232015	Internal capsule haemorrhage	52201006	CPRD Aurum
502878012	Cerebellar haemorrhage	75038005	CPRD Aurum
503469016	Pontine haemorrhage	7713009	CPRD Aurum
1227591017	[V]Personal history of stroke	275526006	CPRD Aurum
1227592012	[V]Personal history of cerebrovascular accident (CVA)	266995000	CPRD Aurum
2476091017	H/O: stroke	275526006	CPRD Aurum
122371000006118	Stroke due to intracerebral haemorrhage	274100004	CPRD Aurum
122401000006115	Stroke unspecified	230690007	CPRD Aurum
123441000006112	Subarachnoid haemorrh from intracranial artery, unspecif	21454007	CPRD Aurum
123481000006118	Subarachnoid haemorrhage from anterior communicating artery	21454007	CPRD Aurum
123491000006115	Subarachnoid haemorrhage from basilar artery	276284000	CPRD Aurum
123511000006114	Subarachnoid haemorrhage from middle cerebral artery	21454007	CPRD Aurum
123521000006118	Subarachnoid haemorrhage from posterior communicating artery	21454007	CPRD Aurum
163261000006119	Right sided intracerebral haemorrhage, unspecified	195168007	CPRD Aurum
306621000000116	Seen in stroke clinic	201501000000108	CPRD Aurum
428181000006115	[X]Subarachnoid haemorrh from intracranial artery, unspecif	21454007	CPRD Aurum
495061000006113	Aspiration of haematoma of brain tissue	171502001	CPRD Aurum
503791000006114	Basal nucleus haemorrhage	195165005	CPRD Aurum
524511000006116	Brain stem stroke syndrome	195212005	CPRD Aurum

605471000006112	CVA - cerebrovascular accid due to intracerebral haemorrhage	274100004	CPRD Aurum
605491000006113	CVA - Cerebrovascular accident unspecified	230690007	CPRD Aurum
605501000006117	CVA unspecified	230690007	CPRD Aurum
744901000006114	Intracerebral haemorrhage	274100004	CPRD Aurum
744921000006116	Intracerebral haemorrhage in hemisphere, unspecified	274100004	CPRD Aurum
746571000006116	Intracerebral haemorrhage, multiple localized	195169004	CPRD Aurum
748941000006115	Left sided intracerebral haemorrhage, unspecified	274100004	CPRD Aurum
809421000006116	H/O: CVA/stroke	275526006	CPRD Aurum
884421000006119	Cerebral haemorrhage	274100004	CPRD Aurum
884451000006111	Cerebral haemorrhage NOS	700251000000105	CPRD Aurum
884521000006115	Stroke/CVA - undefined	685631000000102	CPRD Aurum
884531000006117	Stroke	685631000000102	CPRD Aurum
894091000006111	Cerebral haemorrhage - birth	206196005	CPRD Aurum
907581000006119	[RFC] Stroke/CVA	907581000006103	CPRD Aurum
907591000006116	[RFC] Stroke	907591000006100	CPRD Aurum
908801000006114	[RFC] Stroke	908801000006105	CPRD Aurum
968341000006116	Annual Stroke/CVA blood test	968341000006100	CPRD Aurum
989201000006117	Cerebral haemorrhage	274100004	CPRD Aurum
989211000006119	Cerebral haemorrhage NOS	274100004	CPRD Aurum
1160471000000110	Stroke 6 month review	519751000000106	CPRD Aurum
2126181000000110	Stroke annual review	699270006	CPRD Aurum
2622631000006110	Intrapontine haemorrhage	7713009	CPRD Aurum
2622641000006110	Intrapontine hemorrhage	7713009	CPRD Aurum
2622651000006110	Pontine hemorrhage	7713009	CPRD Aurum
2666411000006110	Evacuation of intracerebral hematoma	10458001	CPRD Aurum
2836981000006110	Perinatal subarachnoid hemorrhage	21202004	CPRD Aurum
2837001000006110	Subarachnoid haemorrhage from any perinatal cause	21202004	CPRD Aurum
2837011000006110	Perinatal intracranial subarachnoid haemorrhage	21202004	CPRD Aurum
2841141000006110	SAH - Subarachnoid haemorrhage	21454007	CPRD Aurum
2841151000006110	SAH - Subarachnoid hemorrhage	21454007	CPRD Aurum
2841161000006110	Subarachnoid intracranial haemorrhage	21454007	CPRD Aurum
2841171000006110	Subarachnoid intracranial hemorrhage	21454007	CPRD Aurum
2871241000006110	Ventricular haemorrhage	23276006	CPRD Aurum
2871261000006110	Intraventricular hemorrhage	23276006	CPRD Aurum
2871271000006110	Intraventricular haemorrhage	23276006	CPRD Aurum
3346051000006110	Internal capsule hemorrhage	52201006	CPRD Aurum
3687811000006110	Cerebral hemisphere haemorrhage	73020009	CPRD Aurum
3719851000006110	Cerebellar hemorrhage	75038005	CPRD Aurum
3850501000006110	Epidural hemorrhage	82999001	CPRD Aurum
4056931000006110	Brain stem haemorrhage	95454007	CPRD Aurum
4057041000006110	Brain stem stroke	95457000	CPRD Aurum
4540671000006110	H/O subarachnoid hemorrhage	161515009	CPRD Aurum
4540681000006110	History of subarachnoid hemorrhage	161515009	CPRD Aurum
4540691000006110	History of subarachnoid haemorrhage	161515009	CPRD Aurum
4644231000006110	Evacuation of hematoma from temporal lobe of brain	171473007	CPRD Aurum
4644251000006110	Evacuation of hematoma from cerebellum	171474001	CPRD Aurum

4644321000006110	Aspiration of brain haematoma	171502001	CPRD Aurum
4644331000006110	Aspiration of brain hematoma	171502001	CPRD Aurum
4777701000006110	Ruptured saccular aneurysm	195154000	CPRD Aurum
4777721000006110	Subarachnoid hemorrhage from carotid siphon and bifurcation	195155004	CPRD Aurum
4777741000006110	Subarachnoid hemorrhage from vertebral artery	195160000	CPRD Aurum
4777751000006110	Intracranial subarachnoid hemorrhage from vertebral artery	195160000	CPRD Aurum
4777761000006110	Intracranial subarachnoid haemorrhage from vertebral artery	195160000	CPRD Aurum
4777831000006110	External capsule hemorrhage	195167002	CPRD Aurum
4777851000006110	Intracerebral hemorrhage, intraventricular	195168007	CPRD Aurum
4777861000006110	Intracerebral haemorrhage with intraventricular haemorrhage	195168007	CPRD Aurum
4777871000006110	Intracerebral hemorrhage with intraventricular hemorrhage	195168007	CPRD Aurum
4777881000006110	Intracerebral haemorrhage, multiple localised	195169004	CPRD Aurum
4777891000006110	Intracerebral hemorrhage, multiple localized	195169004	CPRD Aurum
4778181000006110	Left sided cerebral hemisphere cerebrovascular accident	195216008	CPRD Aurum
4778201000006110	Right sided cerebral hemisphere cerebrovascular accident	195217004	CPRD Aurum
4778331000006110	Sequelae of subarachnoid hemorrhage	195240000	CPRD Aurum
4837741000006110	Cerebellar (nontraumatic) and posterior fossa hemorrhage of fetus and newborn	206419007	CPRD Aurum
4837751000006110	Cerebellar (nontraumatic) and posterior fossa haemorrhage of fetus and newborn	206419007	CPRD Aurum
5010981000006110	Stroke	230690007	CPRD Aurum
5011161000006110	Lacunar stroke	230698000	CPRD Aurum
5011291000006110	Haemorrhagic cerebral infarction	230706003	CPRD Aurum
5011331000006110	Anterior cerebral circulation haemorrhagic infarction	230707007	CPRD Aurum
5011401000006110	Lobar cerebral hemorrhage	230710000	CPRD Aurum
5011581000006110	Subarachnoid haemorrhage due to ruptured aneurysm	230719004	CPRD Aurum
5058411000006110	Ruptured cerebral aneurysm	233983001	CPRD Aurum
5111331000006110	Pituitary haemorrhage	237702003	CPRD Aurum
5146111000006110	Intracerebral haemorrhage in fetus or newborn	240313004	CPRD Aurum
5518041000006110	Spontaneous subarachnoid haemorrhage	270907008	CPRD Aurum
5560161000006110	Intracerebral hemorrhage (ICH)	274100004	CPRD Aurum
5583021000006110	Subarachnoid haemorrhage from middle cerebral artery aneurysm	276280009	CPRD Aurum
5583061000006110	Subarachnoid haemorrhage from anterior communicating artery aneurysm	276282001	CPRD Aurum
5583081000006110	Subarachnoid haemorrhage from posterior communicating artery aneurysm	276283006	CPRD Aurum
5583101000006110	Subarachnoid haemorrhage from basilar artery aneurysm	276284000	CPRD Aurum
5583111000006110	Subarachnoid hemorrhage from basilar artery aneurysm	276284000	CPRD Aurum
5597491000006110	Ruptured aneurysm of middle cerebral artery	277316004	CPRD Aurum
5597601000006110	Ruptured aneurysm of basilar artery	277324009	CPRD Aurum
5597611000006110	Ruptured aneurysm of posterior inferior cerebellar artery	277325005	CPRD Aurum
5898441000006110	Haematoma of brain	301764006	CPRD Aurum
5898461000006110	Cerebellar haematoma	301765007	CPRD Aurum
5960971000006110	Seen by stroke service	306810001	CPRD Aurum
6593131000006110	Nontraumatic epidural hemorrhage	397809001	CPRD Aurum
6837051000006110	Basal ganglion stroke	413102000	CPRD Aurum
7134241000006110	Intraparenchymal haematoma of brain	431266005	CPRD Aurum
7378401000006110	Traumatic intracerebral hemorrhage	450418003	CPRD Aurum
7519441000006110	Cerebrovascular accident annual review	699270006	CPRD Aurum

7951271000006110	Haemorrhage of medulla oblongata	732923001	CPRD Aurum
7951281000006110	Hemorrhage of medulla oblongata	732923001	CPRD Aurum
8089851000006110	Nontraumatic intracerebral haemorrhage	291571000119106	CPRD Aurum
8195421000006110	Cerebrovascular accident (CVA) 6 month review	519751000000106	CPRD Aurum
8195431000006110	Stroke/cerebrovascular accident 6 month review	519751000000106	CPRD Aurum
8221321000006110	Stroke and TIA	720191000000104	CPRD Aurum
8265471000006110	Acute stroke care	817701000000103	CPRD Aurum
9912271000006110	Cerebellar stroke	16371781000100000	CPRD Aurum
11903571000000000	Stroke due to intracerebral haemorrhage	274100004	CPRD Aurum
11919571000000000	[X]Other intracerebral haemorrhage	274100004	CPRD Aurum
11926191000000000	Intracerebral (nontraumatic) haemorrhage of fet and newborn	206417009	CPRD Aurum
12223101000000000	Intracerebral haemorrhage	274100004	CPRD Aurum
12223111000000000	Intracerebral hemorrhage	274100004	CPRD Aurum
12223121000000000	ICH - intracerebral haemorrhage	274100004	CPRD Aurum
12223131000000000	ICH - intracerebral hemorrhage	274100004	CPRD Aurum
12722481000000000	Right sided intracerebral haemorrhage, unspecified	308128006	CPRD Aurum
12727691000000000	Stroke and cerebrovascular accident unspecified	685631000000102	CPRD Aurum
12761901000000000	Subarachnoid hemorrhage	21454007	CPRD Aurum
12762021000000000	Cerebral hemorrhage	274100004	CPRD Aurum
13031191000000000	Cerebral hemorrhage	274100004	CPRD Aurum

medcode	description	readcode	database
1786	Subarachnoid haemorrhage	G60..00	CPRD GOLD
1298	CVA unspecified	G66..11	CPRD GOLD
1469	Stroke and cerebrovascular accident unspecified	G66..00	CPRD GOLD
3535	Intracerebral haemorrhage NOS	G61z.00	CPRD GOLD
5051	Intracerebral haemorrhage	G61..00	CPRD GOLD
5871	H/O: stroke	14A7.12	CPRD GOLD
6116	CVA - Cerebrovascular accident unspecified	G66..13	CPRD GOLD
6253	Stroke unspecified	G66..12	CPRD GOLD
6960	CVA - cerebrovascular accid due to intracerebral haemorrhage	G61..11	CPRD GOLD
7017	Evacuation of intracerebral haematoma NEC	7004300	CPRD GOLD
7138	[V]Personal history of cerebrovascular accident (CVA)	ZV12512	CPRD GOLD
7912	Pontine haemorrhage	G614.00	CPRD GOLD
8443	Brain stem stroke syndrome	G663.00	CPRD GOLD
9696	Subarachnoid haemorrhage from posterior communicating artery	G604.00	CPRD GOLD
13564	Cerebellar haemorrhage	G613.00	CPRD GOLD
17322	Cerebellar stroke syndrome	G664.00	CPRD GOLD
17326	Subarachnoid haemorrh from intracranial artery, unspecif	G60X.00	CPRD GOLD
18604	Stroke due to intracerebral haemorrhage	G61..12	CPRD GOLD
19201	Right sided intracerebral haemorrhage, unspecified	G61X100	CPRD GOLD
19348	[V]Personal history of stroke	ZV12511	CPRD GOLD
19412	Subarachnoid haemorrhage from middle cerebral artery	G602.00	CPRD GOLD
23580	Subarachnoid haemorrhage NOS	G60z.00	CPRD GOLD
28314	Left sided intracerebral haemorrhage, unspecified	G61X000	CPRD GOLD
29939	Ruptured berry aneurysm	G600.00	CPRD GOLD
30045	External capsule haemorrhage	G616.00	CPRD GOLD

30202	Intracerebral haemorrhage, intraventricular	G617.00	CPRD GOLD
31060	Intracerebral haemorrhage in hemisphere, unspecified	G61X.00	CPRD GOLD
31500	Evacuation of haematoma from temporal lobe of brain	7004100	CPRD GOLD
31595	Cortical haemorrhage	G610.00	CPRD GOLD
32959	Seen in stroke clinic	9N0p.00	CPRD GOLD
34135	H/O: CVA/stroke	14A7.00	CPRD GOLD
36559	Intracerebral haemorrhage in fetus or newborn	Q200011	CPRD GOLD
40338	Internal capsule haemorrhage	G611.00	CPRD GOLD
41910	Subarachnoid haemorrhage from basilar artery	G605.00	CPRD GOLD
42331	Subarachnoid haemorrhage from anterior communicating artery	G603.00	CPRD GOLD
43682	Evacuation of haematoma from cerebellum	7004200	CPRD GOLD
44740	Sequelae of subarachnoid haemorrhage	G680.00	CPRD GOLD
46179	Aspiration of haematoma of brain tissue	7008200	CPRD GOLD
46316	Basal nucleus haemorrhage	G612.00	CPRD GOLD
48149	Sequelae of intracerebral haemorrhage	G681.00	CPRD GOLD
53810	[X]Other intracerebral haemorrhage	Gyu6200	CPRD GOLD
56007	Subarachnoid haemorrhage from carotid siphon and bifurcation	G601.00	CPRD GOLD
57315	Intracerebral haemorrhage, multiple localized	G618.00	CPRD GOLD
60692	Subarachnoid haemorrhage from vertebral artery	G606.00	CPRD GOLD
62342	Bulbar haemorrhage	G615.00	CPRD GOLD
65745	[X]Other subarachnoid haemorrhage	Gyu6100	CPRD GOLD
66873	H/O: Stroke in last year	14AK.00	CPRD GOLD
96630	[X]Intracerebral haemorrhage in hemisphere, unspecified	Gyu6F00	CPRD GOLD
105100	Stroke 6 month review	662M100	CPRD GOLD
105520	Admission to stroke unit	8Hd6.00	CPRD GOLD
107440	Lobar cerebral haemorrhage	G619.00	CPRD GOLD
107886	Stroke annual review	662e.11	CPRD GOLD
108630	[X]Subarachnoid haemorrh from intracranial artery, unspecif	Gyu6E00	CPRD GOLD
108668	[X]Subarachnoid haemorrhage from other intracranial arteries	Gyu6000	CPRD GOLD

Supplementary Table 15: Diabetes codes

medcodeid	description	Snomed_ct_code	Source
11927551000000000	Insulin dependent diabetes mellitus with ulcer	19429009	CPRD Aurum
11931861000000000	Pre-existing type 2 diabetes mellitus in pregnancy	237627000	CPRD Aurum
12085891000000000	Clinically significant macular oedema of right eye co-occurrent and due to diabetes mellitus	769221001	CPRD Aurum
12085901000000000	Clinically significant macular edema of right eye due to diabetes mellitus	769221001	CPRD Aurum
12085911000000000	Clinically significant macular edema of right eye co-occurrent and due to diabetes mellitus	769221001	CPRD Aurum
12085921000000000	Clinically significant macular oedema of left eye due to diabetes mellitus	769222008	CPRD Aurum
12085931000000000	Clinically significant macular oedema of left eye co-occurrent and due to diabetes mellitus	769222008	CPRD Aurum
12085941000000000	Clinically significant macular edema of left eye co-occurrent and due to diabetes mellitus	769222008	CPRD Aurum
12085951000000000	Clinically significant macular edema of left eye due to diabetes mellitus	769222008	CPRD Aurum
12086281000000000	Diabetic maculopathy of right eye	769244003	CPRD Aurum
12086291000000000	Maculopathy of right eye due to diabetes mellitus	769244003	CPRD Aurum
12086301000000000	Disorder of right macula co-occurrent and due to diabetes mellitus	769244003	CPRD Aurum
12086321000000000	Maculopathy of left eye due to diabetes mellitus	769245002	CPRD Aurum
12086331000000000	Diabetic maculopathy of left eye	769245002	CPRD Aurum
12086341000000000	Disorder of left macula co-occurrent and due to diabetes mellitus	769245002	CPRD Aurum
12166101000000000	Retinopathy co-occurrent and due to diabetes mellitus	4855003	CPRD Aurum
12166111000000000	Retinopathy due to diabetes mellitus	4855003	CPRD Aurum
12189881000000000	Proliferative retinopathy with diabetes mellitus	59276001	CPRD Aurum
12189891000000000	PDR - proliferative diabetic retinopathy	59276001	CPRD Aurum
12189901000000000	Proliferative retinopathy co-occurrent and due to diabetes mellitus	59276001	CPRD Aurum
12220681000000000	Preproliferative retinopathy co-occurrent and due to diabetes mellitus	193349004	CPRD Aurum
12220691000000000	Advanced maculopathy co-occurrent and due to diabetes mellitus	193350004	CPRD Aurum
12220701000000000	Advanced maculopathy with diabetes mellitus	193350004	CPRD Aurum
12221241000000000	Disorder of macula co-occurrent and due to diabetes mellitus	232020009	CPRD Aurum
12221251000000000	Maculopathy due to diabetes mellitus	232020009	CPRD Aurum
12221261000000000	Maculopathy with diabetes mellitus	232020009	CPRD Aurum
12224581000000000	Advanced retinal disease co-occurrent and due to diabetes mellitus	311782002	CPRD Aurum
12224591000000000	Advanced retinal disease with diabetes mellitus	311782002	CPRD Aurum
12224751000000000	Severe nonproliferative retinopathy co-occurrent and due to diabetes mellitus	312905005	CPRD Aurum
12224761000000000	Severe NPDR (nonproliferative diabetic retinopathy)	312905005	CPRD Aurum
12224811000000000	Macular oedema co-occurrent and due to diabetes mellitus	312912001	CPRD Aurum
12224821000000000	Macular edema co-occurrent and due to diabetes mellitus	312912001	CPRD Aurum
12224831000000000	Macular edema due to diabetes mellitus	312912001	CPRD Aurum
12224841000000000	Macular oedema due to diabetes mallitus	312912001	CPRD Aurum
12295441000000000	Nonproliferative retinopathy co-occurrent and due to diabetes mellitus	390834004	CPRD Aurum
12331051000000000	Mononeuropathy with type 2 diabetes mellitus	420436000	CPRD Aurum
12331061000000000	Mononeuropathy co-occurrent and due to type 2 diabetes mellitus	420436000	CPRD Aurum
12331221000000000	Exudative maculopathy with type 1 diabetes mellitus	420486006	CPRD Aurum
12331231000000000	Exudative maculopathy co-occurrent and due to type 1 diabetes mellitus	420486006	CPRD Aurum
12332271000000000	Retinopathy with type 1 diabetes mellitus	420789003	CPRD Aurum
12332281000000000	Retinopathy co-occurrent and due to type 1 diabetes mellitus	420789003	CPRD Aurum

12332751000000000	Mononeuropathy with type 1 diabetes mellitus	420918009	CPRD Aurum
12332761000000000	Mononeuropathy co-occurrent and due to type 1 diabetes mellitus	420918009	CPRD Aurum
12334131000000000	Neurological disorder co-occurrent and due to type 2 diabetes mellitus	421326000	CPRD Aurum
12334611000000000	Neurological disorder with type 1 diabetes mellitus	421468001	CPRD Aurum
12334621000000000	Neurological disorder co-occurrent and due to type 1 diabetes mellitus	421468001	CPRD Aurum
12335671000000000	Exudative maculopathy with type 2 diabetes mellitus	421779007	CPRD Aurum
12335681000000000	Exudative maculopathy co-occurrent and due to type 2 diabetes mellitus	421779007	CPRD Aurum
12336531000000000	Retinopathy with type 2 diabetes mellitus	422034002	CPRD Aurum
12336541000000000	Retinopathy co-occurrent and due to type 2 diabetes mellitus	422034002	CPRD Aurum
12336871000000000	Disorder of eye with type 2 diabetes mellitus	422099009	CPRD Aurum
12336881000000000	Disorder of eye co-occurrent and due to type 2 diabetes mellitus	422099009	CPRD Aurum
12363661000000000	Gastroparesis with type 1 diabetes mellitus	713702000	CPRD Aurum
12363671000000000	Gastroparesis co-occurrent and due to type 1 diabetes mellitus	713702000	CPRD Aurum
12363681000000000	Gastroparesis with type 2 diabetes mellitus	713703005	CPRD Aurum
12363691000000000	Gastroparesis co-occurrent and due to type 2 diabetes mellitus	713703005	CPRD Aurum
12363721000000000	Polyneuropathy co-occurrent and due to type 1 diabetes mellitus	713705003	CPRD Aurum
12363731000000000	Polyneuropathy co-occurrent and due to type 2 diabetes mellitus	713706002	CPRD Aurum
12370271000000000	Disorder of eye with type 1 diabetes mellitus	739681000	CPRD Aurum
12370281000000000	Disorder of eye co-occurrent and due to type 1 diabetes mellitus	739681000	CPRD Aurum
12485441000000000	NIDDM with peripheral circulatory disorder	422166005	CPRD Aurum
12489851000000000	Type II diabetes mellitus with exudative maculopathy	421779007	CPRD Aurum
12702361000000000	Diabetic cataract associated with type 1 diabetes mellitus	421920002	CPRD Aurum
12702371000000000	Mononeuropathy associated with type 1 diabetes mellitus	420918009	CPRD Aurum
12702381000000000	Diabetic oculopathy due to type I diabetes mellitus	739681000	CPRD Aurum
12702391000000000	Diabetic cataract associated with type 2 diabetes mellitus	420756003	CPRD Aurum
12702401000000000	Mononeuropathy associated with type 2 diabetes mellitus	420436000	CPRD Aurum
12702411000000000	Type II diabetes mellitus with neurological complications	421326000	CPRD Aurum
12702421000000000	Type II diabetes mellitus with ophthalmic complications	422099009	CPRD Aurum
12704941000000000	Neurological disorder associated with type 1 diabetes mellitus	421468001	CPRD Aurum
12704951000000000	Diabetic retinopathy associated with type 1 diabetes mellitus	420789003	CPRD Aurum
12704961000000000	Diabetic oculopathy associated with type 2 diabetes mellitus	422099009	CPRD Aurum
12704971000000000	Neurologic disorder associated with type 2 diabetes mellitus	421326000	CPRD Aurum
12704981000000000	Diabetic retinopathy associated with type 2 diabetes mellitus	422034002	CPRD Aurum
12704991000000000	Ketoacidotic coma in type 2 diabetes mellitus	421847006	CPRD Aurum
12705001000000000	Exudative maculopathy associated with type 1 diabetes mellitus	420486006	CPRD Aurum
12705011000000000	Exudative maculopathy associated with type 2 diabetes mellitus	421779007	CPRD Aurum
12705021000000000	Clinically significant macular oedema of right eye due to diabetes mellitus	769221001	CPRD Aurum
12705071000000000	Gastroparesis due to type 1 diabetes mellitus	713702000	CPRD Aurum
12705081000000000	Gastroparesis due to type 2 diabetes mellitus	713703005	CPRD Aurum
12762301000000000	Diabetic oculopathy associated with type 2 diabetes mellitus	422099009	CPRD Aurum
1223147012	Insulin treated Type II diabetes mellitus	237599002	CPRD Aurum
1223148019	Insulin treated non-insulin dependent diabetes mellitus	237599002	CPRD Aurum
1484867016	Non proliferative diabetic retinopathy	390834004	CPRD Aurum
1484887015	O/E - diabetic maculopathy present both eyes	390854003	CPRD Aurum
1488898011	Hyperosmolar non-ketotic state in type 2 diabetes mellitus	395204000	CPRD Aurum

1665751000000110	Type 1 diabetic dietary review	754101000000103	CPRD Aurum
1667891000000110	Hyperosmolar non-ketotic state in type II diabetes mellitus	395204000	CPRD Aurum
1667921000000110	Type II diabetes mellitus with gastroparesis	713703005	CPRD Aurum
1667941000000110	Type I diabetes mellitus with gastroparesis	713702000	CPRD Aurum
169731000006118	Retinal abnormality - diabetes related	4855003	CPRD Aurum
1713231000006110	QDiabetes (QDScore) type 2 diabetes 10 year risk	1713231000006100	CPRD Aurum
1780311019	Type 1 diabetes mellitus with persistent microalbuminuria	401110002	CPRD Aurum
1780981000006110	Diabetic on non-insulin injectable	1780981000006100	CPRD Aurum
1785163015	High risk proliferative diabetic retinopathy	312907002	CPRD Aurum
1785332013	Background diabetic retinopathy	390834004	CPRD Aurum
1966311000006110	Diabetic on oral treatment and glucagon-like peptide 1	976341000000101	CPRD Aurum
197761014	Type 2 diabetes mellitus	44054006	CPRD Aurum
197984010	Type 1 diabetes mellitus	46635009	CPRD Aurum
1988741000006110	Pre-existing type 2 diabetes mellitus in pregnancy	237627000	CPRD Aurum
2159948010	Diabetic retinopathy 12 month review	408384004	CPRD Aurum
2159949019	Diabetic retinopathy 6 month review	408385003	CPRD Aurum
2159973010	O/E - right eye background diabetic retinopathy	408409007	CPRD Aurum
2159974016	O/E - left eye background diabetic retinopathy	408410002	CPRD Aurum
2159975015	O/E - right eye preproliferative diabetic retinopathy	408411003	CPRD Aurum
2159976019	O/E - left eye preproliferative diabetic retinopathy	408412005	CPRD Aurum
2159977011	O/E - right eye proliferative diabetic retinopathy	408413000	CPRD Aurum
2159978018	O/E - left eye proliferative diabetic retinopathy	408414006	CPRD Aurum
2159979014	O/E - right eye diabetic maculopathy	769244003	CPRD Aurum
2159980012	O/E - left eye diabetic maculopathy	769245002	CPRD Aurum
2288011000000110	Type I diabetes mellitus in remission	703137001	CPRD Aurum
2288041000000110	Type 1 diabetes mellitus in remission	703137001	CPRD Aurum
2288061000000110	Type II diabetes mellitus in remission	703138006	CPRD Aurum
2288071000000110	Type 2 diabetes mellitus in remission	703138006	CPRD Aurum
2359401000000110	Conversion to non-insulin injectable medication	918891000000108	CPRD Aurum
2460251000000110	Diabetic on non-insulin injectable medication	719566006	CPRD Aurum
2474726011	Pan retinal photocoagulation for diabetes	413180006	CPRD Aurum
2549896013	O/E - sight threatening diabetic retinopathy	417677008	CPRD Aurum
2576431000006110	DR - Diabetic retinopathy	4855003	CPRD Aurum
264679015	Diabetic on diet only	170745003	CPRD Aurum
264681018	Diabetic on oral treatment	170746002	CPRD Aurum
280511000006113	Non-insulin dependent diabetes mellitus with hypoglyca coma	719216001	CPRD Aurum
280521000006117	Non-insulin dependent diabetes mellitus with mononeuropathy	420436000	CPRD Aurum
280531000006119	Non-insulin dependent diabetes mellitus with nephropathy	420279001	CPRD Aurum
280541000006112	Non-insulin dependent diabetes mellitus with polyneuropathy	713706002	CPRD Aurum
280551000006114	Non-insulin dependent diabetes mellitus with ulcer	190389009	CPRD Aurum
280561000006111	Non-insulin-dependent d m with peripheral angiopath	314902007	CPRD Aurum
280571000006116	Non-insulin dependent diabetes mellitus	44054006	CPRD Aurum
280581000006118	Non-insulin-dependent diabetes mellitus with multiple comps	190388001	CPRD Aurum
280591000006115	Non-insulin-dependent diabetes mellitus with neuro comps	421326000	CPRD Aurum
281161000006114	Non-insulin depend diabetes mellitus with diabetic cataract	420756003	CPRD Aurum

281171000006119	Non-insulin dependent diabetes mellitus - poor control	443694000	CPRD Aurum
281181000006116	Non-insulin dependent d m with neuropathic arthropathy	314904008	CPRD Aurum
281211000006117	Non-insulin dependent diabetes mellitus with gangrene	421631007	CPRD Aurum
2906401000006110	Diabetic retinal microaneurysm	25412000	CPRD Aurum
292538019	Type I diabetes mellitus with ulcer	190368000	CPRD Aurum
292540012	Type 1 diabetes mellitus with ulcer	190368000	CPRD Aurum
292541011	Type 1 diabetes mellitus with gangrene	420825003	CPRD Aurum
292543014	Type I diabetes mellitus with gangrene	420825003	CPRD Aurum
292548017	Type 1 diabetes mellitus - poor control	444073006	CPRD Aurum
292550013	Type I diabetes mellitus - poor control	444073006	CPRD Aurum
292551012	Type I diabetes mellitus maturity onset	190372001	CPRD Aurum
292553010	Type 1 diabetes mellitus maturity onset	190372001	CPRD Aurum
292576013	Type II diabetes mellitus with multiple complications	190388001	CPRD Aurum
292577016	Type 2 diabetes mellitus with multiple complications	190388001	CPRD Aurum
292579018	Type 2 diabetes mellitus with ulcer	190389009	CPRD Aurum
292581016	Type II diabetes mellitus with ulcer	190389009	CPRD Aurum
292582011	Type II diabetes mellitus with gangrene	421631007	CPRD Aurum
292583018	Type 2 diabetes mellitus with gangrene	421631007	CPRD Aurum
292589019	Type II diabetes mellitus - poor control	443694000	CPRD Aurum
292590011	Type 2 diabetes mellitus - poor control	443694000	CPRD Aurum
2967831017	Pre-existing type 1 diabetes mellitus in pregnancy	199223000	CPRD Aurum
297754014	Preproliferative diabetic retinopathy	193349004	CPRD Aurum
297755010	Advanced diabetic maculopathy	193350004	CPRD Aurum
297758012	Diabetic retinopathy NOS	4855003	CPRD Aurum
299601000000114	Type 1 diabetes mellitus with gastroparesis	713702000	CPRD Aurum
299621000000117	Type 2 diabetes mellitus with gastroparesis	713703005	CPRD Aurum
306113018	Pre-existing diabetes mellitus, non-insulin-dependent	199230006	CPRD Aurum
347657010	Diabetic maculopathy	232020009	CPRD Aurum
3537388019	Diabetic oculopathy due to type 1 diabetes mellitus	739681000	CPRD Aurum
429970018	Unstable type I diabetes mellitus	290002008	CPRD Aurum
429971019	Unstable type 1 diabetes mellitus	290002008	CPRD Aurum
429972014	Unstable insulin dependent diabetes mellitus	11530004	CPRD Aurum
455408014	Advanced diabetic retinal disease	311782002	CPRD Aurum
457325013	Type I diabetes mellitus without complication	313435000	CPRD Aurum
457326014	Type 1 diabetes mellitus without complication	313435000	CPRD Aurum
457328010	Non-insulin-dependent diabetes mellitus without complication	313436004	CPRD Aurum
457329019	Type 2 diabetes mellitus without complication	313436004	CPRD Aurum
457330012	Type II diabetes mellitus without complication	313436004	CPRD Aurum
459161015	Type 1 diabetes mellitus with hypoglycaemic coma	314771006	CPRD Aurum
459162010	Insulin dependent diabetes mellitus with hypoglycaemic coma	237632004	CPRD Aurum
459163017	Type I diabetes mellitus with hypoglycaemic coma	314771006	CPRD Aurum
459167016	Type 2 diabetes mellitus with hypoglycaemic coma	719216001	CPRD Aurum
459169018	Type II diabetes mellitus with hypoglycaemic coma	719216001	CPRD Aurum
459292011	Type 1 diabetes mellitus with arthropathy	314893005	CPRD Aurum
459293018	Insulin dependent diabetes mellitus with arthropathy	39710007	CPRD Aurum
459294012	Type I diabetes mellitus with arthropathy	314893005	CPRD Aurum

459295013	Type I diabetes mellitus with neuropathic arthropathy	7177100011910<br>0	CPRD Aurum
459296014	Type 1 diabetes mellitus with neuropathic arthropathy	7177100011910<br>0	CPRD Aurum
459306016	Type II diabetes mellitus with peripheral angiopathy	314902007	CPRD Aurum
459308015	Type 2 diabetes mellitus with peripheral angiopathy	314902007	CPRD Aurum
459309011	Non-insulin dependent diabetes mellitus with arthropathy	314903002	CPRD Aurum
459310018	Type 2 diabetes mellitus with arthropathy	314903002	CPRD Aurum
459311019	Type II diabetes mellitus with arthropathy	314903002	CPRD Aurum
459312014	Type II diabetes mellitus with neuropathic arthropathy	314904008	CPRD Aurum
459313016	Type 2 diabetes mellitus with neuropathic arthropathy	314904008	CPRD Aurum
4636411000006110	Diet controlled diabetes mellitus	170745003	CPRD Aurum
4757991000006110	Type 1 diabetes mellitus with hyperosmolar coma	190330002	CPRD Aurum
4758011000006110	Type 2 diabetes mellitus with hyperosmolar coma	190331003	CPRD Aurum
4771421000006110	PPDR - Preproliferative diabetic retinopathy	193349004	CPRD Aurum
4796321000006110	Pre-existing type 2 diabetes mellitus	199230006	CPRD Aurum
493773010	NIDDM - Non-insulin dependent diabetes mellitus	44054006	CPRD Aurum
493774016	Type II diabetes mellitus	44054006	CPRD Aurum
494564012	Type I diabetes mellitus	46635009	CPRD Aurum
5110001000006110	Pregnancy and type 2 diabetes mellitus	237627000	CPRD Aurum
5754581000006110	Brittle type 1 diabetes mellitus	290002008	CPRD Aurum
5754611000006110	Brittle type I diabetes mellitus	290002008	CPRD Aurum
5754621000006110	Labile type I diabetes mellitus	290002008	CPRD Aurum
587111000006111	Non-insulin-dependent diabetes mellitus with ophthalm comps	422099009	CPRD Aurum
587521000006111	Non-insulin-dependent diabetes mellitus with renal comps	420279001	CPRD Aurum
6022721000006110	Proliferative diabetic retinopathy - high risk	312907002	CPRD Aurum
6022921000006110	Diabetic macular oedema	312912001	CPRD Aurum
6022931000006110	Diabetic macular edema	312912001	CPRD Aurum
6050041000006110	Insulin dependent diabetes mellitus with hypoglycaemic coma	314771006	CPRD Aurum
6050061000006110	Type I diabetes mellitus with hypoglycemic coma	314771006	CPRD Aurum
6050071000006110	Insulin dependent diabetes mellitus with hypoglycemic coma	314771006	CPRD Aurum
6050081000006110	Type 1 diabetes mellitus with hypoglycemic coma	314771006	CPRD Aurum
6051171000006110	Insulin dependent diabetes mellitus with arthropathy	314893005	CPRD Aurum
641581000006115	Non-insulin-dependent diabetes mellitus with retinopathy	422034002	CPRD Aurum
6515171000006110	Nonproliferative diabetic retinopathy	390834004	CPRD Aurum
6515201000006110	NPDR - Non proliferative diabetic retinopathy	390834004	CPRD Aurum
6515211000006110	BDR - Background diabetic retinopathy	390834004	CPRD Aurum
6515451000006110	On examination - diabetic maculopathy present both eyes	390854003	CPRD Aurum
6630591000006110	Diabetic retinal venous beading	399866003	CPRD Aurum
674961000006118	NIDDM with peripheral circulatory disorder	422166005	CPRD Aurum
6761781000006110	On examination - right eye background diabetic retinopathy	408409007	CPRD Aurum
6761801000006110	On examination - left eye background diabetic retinopathy	408410002	CPRD Aurum
6761821000006110	On examination - right eye preproliferative diabetic retinopathy	408411003	CPRD Aurum
6761841000006110	On examination - left eye preproliferative diabetic retinopathy	408412005	CPRD Aurum
6761861000006110	On examination - right eye proliferative diabetic retinopathy	408413000	CPRD Aurum
6761881000006110	On examination - left eye proliferative diabetic retinopathy	408414006	CPRD Aurum
6837961000006110	Panretinal photocoagulation for diabetes	413180006	CPRD Aurum

6866541000006110	On examination - left eye stable treated proliferative diabetic retinopathy	414894003	CPRD Aurum
6866901000006110	On examination - right eye stable treated proliferative diabetic retinopathy	414910007	CPRD Aurum
6910411000006110	On examination - sight threatening diabetic retinopathy	417677008	CPRD Aurum
6951081000006110	Ketoacidosis in type I diabetes mellitus	420270002	CPRD Aurum
6951281000006110	Kidney disorder associated with type 2 diabetes mellitus	420279001	CPRD Aurum
6954121000006110	Mononeuropathy associated with type II diabetes mellitus	420436000	CPRD Aurum
6954991000006110	Exudative maculopathy associated with type I diabetes mellitus	420486006	CPRD Aurum
6955501000006110	Persistent proteinuria associated with type I diabetes mellitus	420514000	CPRD Aurum
6959091000006110	Persistent microalbuminuria associated with type II diabetes mellitus	420715001	CPRD Aurum
6959801000006110	Diabetic cataract associated with type II diabetes mellitus	420756003	CPRD Aurum
6960431000006110	Diabetic retinopathy associated with type I diabetes mellitus	420789003	CPRD Aurum
6960441000006110	Diabetes type 1 with retinopathy	420789003	CPRD Aurum
6961081000006110	Gangrene associated with type 1 diabetes mellitus	420825003	CPRD Aurum
6961091000006110	Gangrene associated with type I diabetes mellitus	420825003	CPRD Aurum
6961901000006110	Disorder associated with type I diabetes mellitus	420868002	CPRD Aurum
6962751000006110	Mononeuropathy associated with type I diabetes mellitus	420918009	CPRD Aurum
6965561000006110	Ketoacidotic coma in type I diabetes mellitus	421075007	CPRD Aurum
6969921000006110	Neurologic disorder associated with type II diabetes mellitus	421326000	CPRD Aurum
6970621000006110	Peripheral circulatory disorder associated with type 1 diabetes mellitus	421365002	CPRD Aurum
6970631000006110	Peripheral circulatory disorder associated with type I diabetes mellitus	421365002	CPRD Aurum
6972441000006110	Neurological disorder associated with type I diabetes mellitus	421468001	CPRD Aurum
6975211000006110	Gangrene associated with type 2 diabetes mellitus	421631007	CPRD Aurum
6975221000006110	Gangrene associated with type II diabetes mellitus	421631007	CPRD Aurum
6977421000006110	Ketoacidosis in type II diabetes mellitus	421750000	CPRD Aurum
6977971000006110	Exudative maculopathy associated with type II diabetes mellitus	421779007	CPRD Aurum
6979991000006110	Renal disorder associated with type I diabetes mellitus	421893009	CPRD Aurum
6980001000006110	Kidney disorder associated with type 1 diabetes mellitus	421893009	CPRD Aurum
6980431000006110	Diabetic cataract associated with type I diabetes mellitus	421920002	CPRD Aurum
6981621000006110	Persistent proteinuria associated with type II diabetes mellitus	421986006	CPRD Aurum
6982151000006110	Disorder due to type II diabetes mellitus	422014003	CPRD Aurum
6982511000006110	Diabetic retinopathy associated with type II diabetes mellitus	422034002	CPRD Aurum
6982521000006110	Diabetes type 2 with retinopathy	422034002	CPRD Aurum
6983691000006110	Diabetic oculopathy associated with type II diabetes mellitus	422099009	CPRD Aurum
6985001000006110	Peripheral circulatory disorder associated with type 2 diabetes mellitus	422166005	CPRD Aurum
6985011000006110	Peripheral circulatory disorder associated with type II diabetes mellitus	422166005	CPRD Aurum
6986151000006110	Multiple complications of type I diabetes mellitus	422228004	CPRD Aurum
72651000006114	Unstable insulin dependent diabetes mellitus	11530004	CPRD Aurum
72711000006117	Unstable type 1 diabetes mellitus	290002008	CPRD Aurum
72721000006113	Unstable type I diabetes mellitus	290002008	CPRD Aurum
7281511000006110	Type 2 diabetes mellitus uncontrolled	443694000	CPRD Aurum
7287611000006110	Type 1 diabetes mellitus uncontrolled	444073006	CPRD Aurum
7287621000006110	Type I diabetes mellitus uncontrolled	444073006	CPRD Aurum
733161000000116	Impaired vision due to diabetic retinopathy	373041000000101	CPRD Aurum
73466011	Non-insulin dependent diabetes mellitus	44054006	CPRD Aurum

7708091000006110	Polyneuropathy due to type 1 diabetes mellitus	713705003	CPRD Aurum
771331000006116	Insulin dependent diabetes mellitus with arthropathy	39710007	CPRD Aurum
771341000006114	Insulin dependent diabetes mellitus with diabetic cataract	43959009	CPRD Aurum
771351000006111	Insulin dependent diabetes mellitus with gangrene	422275004	CPRD Aurum
771361000006113	Insulin dependent diabetes mellitus with hypoglycaemic coma	237632004	CPRD Aurum
771371000006118	Insulin dependent diabetes mellitus with mononeuropathy	230577008	CPRD Aurum
771381000006115	Insulin dependent diabetes mellitus with multiple complicatn	385041000000108	CPRD Aurum
771391000006117	Insulin dependent diabetes mellitus with nephropathy	127013003	CPRD Aurum
771401000006115	Insulin dependent diabetes mellitus with polyneuropathy	49455004	CPRD Aurum
771411000006117	Insulin dependent diabetes mellitus with retinopathy	4855003	CPRD Aurum
771421000006113	Insulin dependent diabetes mellitus with ulcer	422183001	CPRD Aurum
772131000006111	Insulin dependent diab mell with neuropathic arthropathy	201724008	CPRD Aurum
772141000006118	Insulin dependent diab mell with peripheral angiopathy	421895002	CPRD Aurum
772151000006116	Insulin dependent diabetes maturity onset	73211009	CPRD Aurum
772161000006119	Insulin dependent diabetes mellitus	73211009	CPRD Aurum
772171000006114	Insulin dependent diabetes mellitus	73211009	CPRD Aurum
772181000006112	Insulin dependent diabetes mellitus - poor control	268519009	CPRD Aurum
77727018	Insulin dependent diabetes mellitus	73211009	CPRD Aurum
787111000006112	IDDM-Insulin dependent diabetes mellitus	73211009	CPRD Aurum
7966931000006110	Angina associated with type 2 diabetes mellitus	791000119109	CPRD Aurum
7966941000006110	Angina associated with type II diabetes mellitus	791000119109	CPRD Aurum
7966951000006110	Diabetic angina pectoris associated with type 2 diabetes mellitus	791000119109	CPRD Aurum
8014491000006110	Peripheral vascular disease due to type I diabetes	31211000119101	CPRD Aurum
8022441000006110	Neuropathic arthropathy due to type 1 diabetes mellitus	71771000119100	CPRD Aurum
8022461000006110	Diabetic neuropathic arthropathy due to type 1 diabetes mellitus	71771000119100	CPRD Aurum
840951000006119	Insulin treated Type 2 diabetes mellitus	237599002	CPRD Aurum
841011000006112	High risk non proliferative diabetic retinopathy	312905005	CPRD Aurum
841351000006110	Insulin treated Type II diabetes mellitus	237599002	CPRD Aurum
84281000006115	Type 1 diabetes mellitus	46635009	CPRD Aurum
84291000006117	Type 1 diabetes mellitus - poor control	444073006	CPRD Aurum
84301000006116	Type 1 diabetes mellitus maturity onset	190372001	CPRD Aurum
84311000006118	Type 1 diabetes mellitus with arthropathy	314893005	CPRD Aurum
84321000006114	Type 1 diabetes mellitus with diabetic cataract	421920002	CPRD Aurum
84331000006112	Type 1 diabetes mellitus with gangrene	420825003	CPRD Aurum
84341000006119	Type 1 diabetes mellitus with hypoglycaemic coma	314771006	CPRD Aurum
84351000006117	Type 1 diabetes mellitus with mononeuropathy	420918009	CPRD Aurum
84361000006115	Type 1 diabetes mellitus with multiple complications	422228004	CPRD Aurum
84371000006110	Type 1 diabetes mellitus with nephropathy	421893009	CPRD Aurum
84381000006113	Type 1 diabetes mellitus with neurological complications	421468001	CPRD Aurum
84391000006111	Type 1 diabetes mellitus with neuropathic arthropathy	71771000119100	CPRD Aurum
84401000006113	Type 1 diabetes mellitus with ophthalmic complications	739681000	CPRD Aurum
84411000006111	Type 1 diabetes mellitus with peripheral angiopathy	31211000119101	CPRD Aurum
84421000006115	Type 1 diabetes mellitus with polyneuropathy	713705003	CPRD Aurum
84431000006117	Type 1 diabetes mellitus with renal complications	421893009	CPRD Aurum

84441000006110	Type 1 diabetes mellitus with retinopathy	420789003	CPRD Aurum
84451000006112	Type 1 diabetes mellitus with ulcer	190368000	CPRD Aurum
84461000006114	Type 1 diabetes mellitus without complication	313435000	CPRD Aurum
84471000006119	Type 2 diabetes mellitus	44054006	CPRD Aurum
84481000006116	Type 2 diabetes mellitus - poor control	443694000	CPRD Aurum
84491000006118	Type 2 diabetes mellitus with arthropathy	314903002	CPRD Aurum
84501000006114	Type 2 diabetes mellitus with diabetic cataract	420756003	CPRD Aurum
84511000006112	Type 2 diabetes mellitus with gangrene	421631007	CPRD Aurum
84521000006116	Type 2 diabetes mellitus with hypoglycaemic coma	719216001	CPRD Aurum
84531000006118	Type 2 diabetes mellitus with mononeuropathy	420436000	CPRD Aurum
84541000006111	Type 2 diabetes mellitus with multiple complications	190388001	CPRD Aurum
84551000006113	Type 2 diabetes mellitus with nephropathy	420279001	CPRD Aurum
84561000006110	Type 2 diabetes mellitus with neurological complications	421326000	CPRD Aurum
84571000006115	Type 2 diabetes mellitus with neuropathic arthropathy	314904008	CPRD Aurum
84581000006117	Type 2 diabetes mellitus with ophthalmic complications	422099009	CPRD Aurum
84591000006119	Type 2 diabetes mellitus with peripheral angiopathy	314902007	CPRD Aurum
84601000006110	Type 2 diabetes mellitus with polyneuropathy	713706002	CPRD Aurum
84611000006113	Type 2 diabetes mellitus with renal complications	420279001	CPRD Aurum
84621000006117	Type 2 diabetes mellitus with retinopathy	422034002	CPRD Aurum
84631000006119	Type 2 diabetes mellitus with ulcer	190389009	CPRD Aurum
84641000006112	Type 2 diabetes mellitus without complication	313436004	CPRD Aurum
84651000006114	Type I diabetes mellitus	46635009	CPRD Aurum
84661000006111	Type I diabetes mellitus - poor control	444073006	CPRD Aurum
84671000006116	Type I diabetes mellitus maturity onset	190372001	CPRD Aurum
84681000006118	Type I diabetes mellitus with arthropathy	314893005	CPRD Aurum
84691000006115	Type I diabetes mellitus with diabetic cataract	421920002	CPRD Aurum
84701000006115	Type I diabetes mellitus with gangrene	420825003	CPRD Aurum
84711000006117	Type I diabetes mellitus with hypoglycaemic coma	314771006	CPRD Aurum
84721000006113	Type I diabetes mellitus with mononeuropathy	420918009	CPRD Aurum
84731000006111	Type I diabetes mellitus with multiple complications	422228004	CPRD Aurum
84741000006118	Type I diabetes mellitus with nephropathy	421893009	CPRD Aurum
84751000006116	Type I diabetes mellitus with neurological complications	421468001	CPRD Aurum
84761000006119	Type I diabetes mellitus with neuropathic arthropathy	71771000119100	CPRD Aurum
84771000006114	Type I diabetes mellitus with ophthalmic complications	739681000	CPRD Aurum
84781000006112	Type I diabetes mellitus with peripheral angiopathy	31211000119101	CPRD Aurum
84791000006110	Type I diabetes mellitus with polyneuropathy	713705003	CPRD Aurum
84801000006111	Type I diabetes mellitus with renal complications	421893009	CPRD Aurum
84811000006114	Type I diabetes mellitus with retinopathy	420789003	CPRD Aurum
84821000006118	Type I diabetes mellitus with ulcer	190368000	CPRD Aurum
84831000006115	Type I diabetes mellitus without complication	313435000	CPRD Aurum
84841000006113	Type II diabetes mellitus	44054006	CPRD Aurum
84851000006110	Type II diabetes mellitus - poor control	443694000	CPRD Aurum
84861000006112	Type II diabetes mellitus with arthropathy	314903002	CPRD Aurum
84871000006117	Type II diabetes mellitus with diabetic cataract	420756003	CPRD Aurum
84881000006119	Type II diabetes mellitus with gangrene	421631007	CPRD Aurum

84891000006116	Type II diabetes mellitus with hypoglycaemic coma	719216001	CPRD Aurum
84901000006117	Type II diabetes mellitus with mononeuropathy	420436000	CPRD Aurum
84911000006119	Type II diabetes mellitus with multiple complications	190388001	CPRD Aurum
84921000006110	Type II diabetes mellitus with nephropathy	420279001	CPRD Aurum
84931000006113	Type II diabetes mellitus with neurological complications	421326000	CPRD Aurum
84941000006115	Type II diabetes mellitus with neuropathic arthropathy	314904008	CPRD Aurum
84951000006118	Type II diabetes mellitus with ophthalmic complications	422099009	CPRD Aurum
84961000006116	Type II diabetes mellitus with peripheral angiopathy	314902007	CPRD Aurum
84971000006111	Type II diabetes mellitus with polyneuropathy	713706002	CPRD Aurum
84981000006114	Type II diabetes mellitus with renal complications	420279001	CPRD Aurum
84991000006112	Type II diabetes mellitus with retinopathy	422034002	CPRD Aurum
85001000006117	Type II diabetes mellitus with ulcer	190389009	CPRD Aurum
85011000006119	Type II diabetes mellitus without complication	313436004	CPRD Aurum
850691000006118	Hyperosmolar non-ketotic state in type 2 diabetes mellitus	395204000	CPRD Aurum
856611000006114	Laser treated diabetic retinopathy	856611000006105	CPRD Aurum
856621000006118	Left proliferative diabetic retinopathy	856621000006102	CPRD Aurum
856631000006115	Left laser treated diabetic retinopathy	856631000006104	CPRD Aurum
856641000006113	Left diabetic maculopathy	856641000006109	CPRD Aurum
856651000006110	Left advanced diabetic retinal disease	856651000006106	CPRD Aurum
857031000006113	Left non-proliferative diabetic retinopathy	857031000006109	CPRD Aurum
857051000006118	Left preproliferative diabetic retinopathy	857051000006102	CPRD Aurum
857411000006110	Right proliferative diabetic retinopathy	857411000006106	CPRD Aurum
857421000006119	Right laser treated diabetic retinopathy	857421000006103	CPRD Aurum
857431000006116	Right diabetic maculopathy	857431000006100	CPRD Aurum
857441000006114	Right advanced diabetic retinal disease	857441000006105	CPRD Aurum
857971000006110	Right non-proliferative diabetic retinopathy	857971000006106	CPRD Aurum
857981000006113	Right preproliferative diabetic retinopathy	857981000006109	CPRD Aurum
9093013	Diabetic retinopathy	4855003	CPRD Aurum
913451000006117	Type 1 diabetes mellitus with renal complications	421893009	CPRD Aurum
913461000006115	Type I diabetes mellitus with renal complications	421893009	CPRD Aurum
913481000006113	Type 1 diabetes mellitus with ophthalmic complications	739681000	CPRD Aurum
913491000006111	Type I diabetes mellitus with ophthalmic complications	739681000	CPRD Aurum
913511000006117	Type 1 diabetes mellitus with neurological complications	421468001	CPRD Aurum
913521000006113	Type I diabetes mellitus with neurological complications	421468001	CPRD Aurum
913531000006111	Insulin dependent diabetes mellitus with multiple complicat	385041000000108	CPRD Aurum
913541000006118	Type 1 diabetes mellitus with multiple complications	422228004	CPRD Aurum
913551000006116	Type I diabetes mellitus with multiple complications	422228004	CPRD Aurum
913591000006110	Insulin dependent diabetes mellitus with ulcer	422183001	CPRD Aurum
913621000006112	Insulin dependent diabetes mellitus with gangrene	422275004	CPRD Aurum
913651000006115	Insulin dependent diabetes mellitus with retinopathy	4855003	CPRD Aurum
913661000006118	Type 1 diabetes mellitus with retinopathy	420789003	CPRD Aurum
913671000006113	Type I diabetes mellitus with retinopathy	420789003	CPRD Aurum

913681000006111	Insulin dependent diabetes mellitus - poor control	268519009	CPRD Aurum
913711000006112	Insulin dependent diabetes maturity onset	73211009	CPRD Aurum
913771000006115	Insulin dependent diabetes mellitus with mononeuropathy	230577008	CPRD Aurum
913781000006117	Type 1 diabetes mellitus with mononeuropathy	420918009	CPRD Aurum
913791000006119	Type I diabetes mellitus with mononeuropathy	420918009	CPRD Aurum
913801000006118	Insulin dependent diabetes mellitus with polyneuropathy	49455004	CPRD Aurum
913811000006115	Type 1 diabetes mellitus with polyneuropathy	713705003	CPRD Aurum
913821000006111	Type I diabetes mellitus with polyneuropathy	713705003	CPRD Aurum
913831000006114	Insulin dependent diabetes mellitus with nephropathy	127013003	CPRD Aurum
913841000006116	Type 1 diabetes mellitus with nephropathy	421893009	CPRD Aurum
913851000006119	Type I diabetes mellitus with nephropathy	421893009	CPRD Aurum
913891000006113	Insulin dependent diabetes mellitus with diabetic cataract	43959009	CPRD Aurum
913901000006112	Type 1 diabetes mellitus with diabetic cataract	421920002	CPRD Aurum
913911000006110	Type I diabetes mellitus with diabetic cataract	421920002	CPRD Aurum
913921000006119	Insulin dependent diab mell with peripheral angiopathy	421895002	CPRD Aurum
913931000006116	Type 1 diabetes mellitus with peripheral angiopathy	31211000119101	CPRD Aurum
913941000006114	Type I diabetes mellitus with peripheral angiopathy	31211000119101	CPRD Aurum
913981000006115	Insulin dependent diab mell with neuropathic arthropathy	201724008	CPRD Aurum
914031000006118	Type 2 diabetes mellitus with renal complications	420279001	CPRD Aurum
914041000006111	Type II diabetes mellitus with renal complications	420279001	CPRD Aurum
914051000006113	Type 2 diabetes mellitus with ophthalmic complications	422099009	CPRD Aurum
914061000006110	Type II diabetes mellitus with ophthalmic complications	422099009	CPRD Aurum
914071000006115	Type 2 diabetes mellitus with neurological complications	421326000	CPRD Aurum
914081000006117	Type II diabetes mellitus with neurological complications	421326000	CPRD Aurum
914151000006112	Type 2 diabetes mellitus with retinopathy	422034002	CPRD Aurum
914161000006114	Type II diabetes mellitus with retinopathy	422034002	CPRD Aurum
914221000006113	Type 2 diabetes mellitus with mononeuropathy	420436000	CPRD Aurum
914231000006111	Type II diabetes mellitus with mononeuropathy	420436000	CPRD Aurum
914241000006118	Type 2 diabetes mellitus with polyneuropathy	713706002	CPRD Aurum
914251000006116	Type II diabetes mellitus with polyneuropathy	713706002	CPRD Aurum
914261000006119	Type 2 diabetes mellitus with nephropathy	420279001	CPRD Aurum
914271000006114	Type II diabetes mellitus with nephropathy	420279001	CPRD Aurum
914301000006111	Type 2 diabetes mellitus with diabetic cataract	420756003	CPRD Aurum
914311000006114	Type II diabetes mellitus with diabetic cataract	420756003	CPRD Aurum
914391000006116	Insulin treated Type 2 diabetes mellitus	237599002	CPRD Aurum
928461000006119	Type 1 diabetes mellitus with persistent proteinuria	420514000	CPRD Aurum
928471000006114	Type I diabetes mellitus with persistent proteinuria	420514000	CPRD Aurum
928491000006110	Type I diabetes mellitus with persistent microalbuminuria	401110002	CPRD Aurum
928501000006119	Type 1 diabetes mellitus with ketoacidosis	420270002	CPRD Aurum
928511000006116	Type I diabetes mellitus with ketoacidosis	420270002	CPRD Aurum
928521000006112	Type 1 diabetes mellitus with ketoacidotic coma	421075007	CPRD Aurum
928531000006110	Type I diabetes mellitus with ketoacidotic coma	421075007	CPRD Aurum
928541000006117	Type 2 diabetes mellitus with persistent proteinuria	421986006	CPRD Aurum
928551000006115	Type II diabetes mellitus with persistent proteinuria	421986006	CPRD Aurum
928561000006118	Type 2 diabetes mellitus with persistent microalbuminuria	420715001	CPRD Aurum

928571000006113	Type II diabetes mellitus with persistent microalbuminuria	420715001	CPRD Aurum
928581000006111	Type 2 diabetes mellitus with ketoacidosis	421750000	CPRD Aurum
928591000006114	Type II diabetes mellitus with ketoacidosis	421750000	CPRD Aurum
928601000006118	Type 2 diabetes mellitus with ketoacidotic coma	421847006	CPRD Aurum
928611000006115	Type II diabetes mellitus with ketoacidotic coma	421847006	CPRD Aurum
938301000006114	Type 1 diabetes mellitus with exudative maculopathy	420486006	CPRD Aurum
938311000006112	Type I diabetes mellitus with exudative maculopathy	420486006	CPRD Aurum
938321000006116	Type 2 diabetes mellitus with exudative maculopathy	421779007	CPRD Aurum
938331000006118	Type II diabetes mellitus with exudative maculopathy	421779007	CPRD Aurum
975251000006111	O/E - right eye stable treated prolif diabetic retinopathy	414910007	CPRD Aurum
975261000006113	O/E - left eye stable treated prolif diabetic retinopathy	414894003	CPRD Aurum
98476015	Proliferative diabetic retinopathy	59276001	CPRD Aurum
**medcode**	**description**	**readcode**	**database**
506	Non-insulin dependent diabetes mellitus	C100112	CPRD GOLD
758	Type 2 diabetes mellitus	C10F.00	CPRD GOLD
1038	Insulin dependent diabetes mellitus	C100011	CPRD GOLD
1323	Diabetic retinopathy	F420.00	CPRD GOLD
1407	Insulin treated Type 2 diabetes mellitus	C10FJ00	CPRD GOLD
1549	Type 1 diabetes mellitus	C10E.00	CPRD GOLD
1647	Insulin dependent diabetes mellitus	C108.00	CPRD GOLD
1684	Diabetic on oral treatment	66A4.00	CPRD GOLD
2986	Preproliferative diabetic retinopathy	F420200	CPRD GOLD
3286	Proliferative diabetic retinopathy	F420100	CPRD GOLD
3837	Diabetic maculopathy	F420400	CPRD GOLD
4513	Non-insulin dependent diabetes mellitus	C109.00	CPRD GOLD
5884	NIDDM - Non-insulin dependent diabetes mellitus	C109.11	CPRD GOLD
6509	Insulin dependent diabetes mellitus with retinopathy	C108700	CPRD GOLD
6791	Insulin dependent diabetes mellitus - poor control	C108800	CPRD GOLD
7069	Background diabetic retinopathy	F420000	CPRD GOLD
7563	Diabetic on diet only	66A3.00	CPRD GOLD
8403	Non-insulin dependent diabetes mellitus - poor control	C109700	CPRD GOLD
9835	O/E - diabetic maculopathy present both eyes	2BBL.00	CPRD GOLD
10099	Advanced diabetic maculopathy	F420300	CPRD GOLD
10418	Type 1 diabetes mellitus with nephropathy	C10ED00	CPRD GOLD
10692	Type 1 diabetes mellitus with ketoacidosis	C10EM00	CPRD GOLD
10755	Non proliferative diabetic retinopathy	F420600	CPRD GOLD
11018	Diabetic retinopathy 12 month review	8HBG.00	CPRD GOLD
11129	O/E - left eye background diabetic retinopathy	2BBQ.00	CPRD GOLD
11433	O/E - right eye background diabetic retinopathy	2BBP.00	CPRD GOLD
11599	Pan retinal photocoagulation for diabetes	7276	CPRD GOLD
11626	Diabetic retinopathy NOS	F420z00	CPRD GOLD
12455	Type I diabetes mellitus	C10E.11	CPRD GOLD
12640	Type 2 diabetes mellitus with nephropathy	C10FC00	CPRD GOLD
12736	Type 2 diabetes mellitus with gangrene	C10F500	CPRD GOLD
13097	O/E - right eye proliferative diabetic retinopathy	2BBT.00	CPRD GOLD
13099	O/E - right eye preproliferative diabetic retinopathy	2BBR.00	CPRD GOLD

13101	O/E - left eye proliferative diabetic retinopathy	2BBV.00	CPRD GOLD
13102	O/E - right eye diabetic maculopathy	2BBW.00	CPRD GOLD
13103	O/E - left eye preproliferative diabetic retinopathy	2BBS.00	CPRD GOLD
13108	O/E - left eye diabetic maculopathy	2BBX.00	CPRD GOLD
17262	Non-insulin-dependent diabetes mellitus with retinopathy	C109600	CPRD GOLD
17545	Type I diabetes mellitus with diabetic cataract	C108F11	CPRD GOLD
17858	Type 1 diabetes mellitus	C108.12	CPRD GOLD
17859	Type 2 diabetes mellitus	C109.12	CPRD GOLD
18143	Type II diabetes mellitus with arthropathy	C109G11	CPRD GOLD
18209	Type 2 diabetes mellitus with renal complications	C109012	CPRD GOLD
18219	Type II diabetes mellitus	C109.13	CPRD GOLD
18230	Type 1 diabetes mellitus with neuropathic arthropathy	C108J12	CPRD GOLD
18264	Insulin treated Type II diabetes mellitus	C109J12	CPRD GOLD
18278	Insulin treated Type 2 diabetes mellitus	C109J00	CPRD GOLD
18387	Type 1 diabetes mellitus with retinopathy	C10E700	CPRD GOLD
18390	Type 2 diabetes mellitus with persistent microalbuminuria	C10FM00	CPRD GOLD
18425	Type 2 diabetes mellitus with polyneuropathy	C10FB00	CPRD GOLD
18496	Type 2 diabetes mellitus with retinopathy	C10F600	CPRD GOLD
18505	IDDM-Insulin dependent diabetes mellitus	C108.11	CPRD GOLD
18642	Type 1 diabetes mellitus with arthropathy	C10EH00	CPRD GOLD
18662	Diabetic retinopathy 6 month review	8HBH.00	CPRD GOLD
18683	Type 1 diabetes mellitus with ulcer	C10E500	CPRD GOLD
18777	Type 2 diabetes mellitus with renal complications	C10F000	CPRD GOLD
21983	Type 1 diabetes mellitus with renal complications	C108012	CPRD GOLD
22871	Type 1 diabetes mellitus with exudative maculopathy	C10EP00	CPRD GOLD
22884	Type II diabetes mellitus	C10F.11	CPRD GOLD
22967	Retinal abnormality - diabetes related	2BBF.00	CPRD GOLD
24423	Type I diabetes mellitus	C108.13	CPRD GOLD
24458	Type II diabetes mellitus - poor control	C109711	CPRD GOLD
24693	Non-insulin dependent diabetes mellitus with arthropathy	C109G00	CPRD GOLD
24694	Insulin dependent diabetes mellitus with mononeuropathy	C108B00	CPRD GOLD
24836	Type 2 diabetes mellitus with nephropathy	C109C12	CPRD GOLD
25041	Dietary advice for type II diabetes	ZC2CA00	CPRD GOLD
25591	Type 2 diabetes mellitus with exudative maculopathy	C10FQ00	CPRD GOLD
25627	Type 2 diabetes mellitus - poor control	C10F700	CPRD GOLD
26054	Type 2 diabetes mellitus with persistent proteinuria	C10FL00	CPRD GOLD
26855	Unstable insulin dependent diabetes mellitus	C108400	CPRD GOLD
29979	Non-insulin-dependent diabetes mellitus without complication	C109900	CPRD GOLD
30294	Type 1 diabetes mellitus with persistent microalbuminuria	C10EL00	CPRD GOLD
30323	Type 1 diabetes mellitus with persistent proteinuria	C10EK00	CPRD GOLD
30477	High risk proliferative diabetic retinopathy	F420700	CPRD GOLD
31310	Insulin dependent diabetes maturity onset	C108900	CPRD GOLD
32627	Type 2 diabetes mellitus with ketoacidosis	C10FN00	CPRD GOLD
34268	Type 2 diabetes mellitus with neurological complications	C10F200	CPRD GOLD
34450	Hyperosmolar non-ketotic state in type 2 diabetes mellitus	C10FK00	CPRD GOLD
34912	Non-insulin dependent diabetes mellitus with ulcer	C109400	CPRD GOLD

35288	Type 1 diabetes mellitus - poor control	C10E800	CPRD GOLD
35385	Type 2 diabetes mellitus with neuropathic arthropathy	C10FH00	CPRD GOLD
36633	Hyperosmolar non-ketotic state in type 2 diabetes mellitus	C109K00	CPRD GOLD
37648	Insulin treated non-insulin dependent diabetes mellitus	C109J11	CPRD GOLD
37806	Type 2 diabetes mellitus with peripheral angiopathy	C10FF00	CPRD GOLD
38161	Type I diabetes mellitus with retinopathy	C108711	CPRD GOLD
39070	Type 1 diabetes mellitus with hypoglycaemic coma	C10EE00	CPRD GOLD
39809	Insulin dependent diab mell with neuropathic arthropathy	C108J00	CPRD GOLD
40401	Non-insulin dependent diabetes mellitus with gangrene	C109500	CPRD GOLD
40682	Type 1 diabetes mellitus maturity onset	C10E900	CPRD GOLD
40837	Type 1 diabetes mellitus with ketoacidotic coma	C10EN00	CPRD GOLD
40962	Non-insulin dependent d m with neuropathic arthropathy	C109H00	CPRD GOLD
41049	Type 1 diabetes mellitus with retinopathy	C108712	CPRD GOLD
41716	Insulin dependent diabetes mellitus with polyneuropathy	C108C00	CPRD GOLD
42729	Type I diabetes mellitus with hypoglycaemic coma	C108E11	CPRD GOLD
42762	Type 2 diabetes mellitus with retinopathy	C109612	CPRD GOLD
42831	Type 1 diabetes mellitus with neurological complications	C10E200	CPRD GOLD
43227	Type II diabetes mellitus with multiple complications	C10F311	CPRD GOLD
43785	Non-insulin dependent diabetes mellitus with hypoglyca coma	C109D00	CPRD GOLD
43921	Unstable type 1 diabetes mellitus	C10E400	CPRD GOLD
44260	Insulin dependent diabetes mellitus with diabetic cataract	C108F00	CPRD GOLD
44440	Insulin dependent diabetes mellitus with hypoglycaemic coma	C108E00	CPRD GOLD
44443	Insulin dependent diabetes mellitus with ulcer	C108500	CPRD GOLD
44779	Type 2 diabetes mellitus with diabetic cataract	C109E12	CPRD GOLD
44982	Type 2 diabetes mellitus with diabetic cataract	C10FE00	CPRD GOLD
45276	Insulin dependent diabetes mellitus with multiple complicat	C10E312	CPRD GOLD
45467	Non-insulin dependent diabetes mellitus with polyneuropathy	C109B00	CPRD GOLD
45913	Type 2 diabetes mellitus - poor control	C109712	CPRD GOLD
45914	Type 1 diabetes mellitus - poor control	C108812	CPRD GOLD
45919	Type 2 diabetes mellitus with neurological complications	C109212	CPRD GOLD
46150	Type 2 diabetes mellitus with gangrene	C109512	CPRD GOLD
46301	Type 1 diabetes mellitus with polyneuropathy	C10EC00	CPRD GOLD
46850	Type I diabetes mellitus - poor control	C108811	CPRD GOLD
46917	Type 2 diabetes mellitus with hypoglycaemic coma	C10FD00	CPRD GOLD
47315	Type II diabetes mellitus - poor control	C10F711	CPRD GOLD
47321	Type 2 diabetes mellitus with ophthalmic complications	C10F100	CPRD GOLD
47328	O/E - right eye stable treated prolif diabetic retinopathy	2BBk.00	CPRD GOLD
47409	Type II diabetes mellitus with polyneuropathy	C109B11	CPRD GOLD
47582	Type 1 diabetes mellitus with renal complications	C10E000	CPRD GOLD
47584	Advanced diabetic retinal disease	F420500	CPRD GOLD
47649	Type 1 diabetes mellitus with ophthalmic complications	C10E100	CPRD GOLD
47650	Type 1 diabetes mellitus with multiple complications	C10E300	CPRD GOLD
47816	Type II diabetes mellitus with neuropathic arthropathy	C109H11	CPRD GOLD
47954	Type 2 diabetes mellitus without complication	C10F900	CPRD GOLD
48192	Type II diabetes mellitus with diabetic cataract	C109E11	CPRD GOLD
49074	Type 2 diabetes mellitus with ulcer	C10F400	CPRD GOLD

49146	Type I diabetes mellitus with neurological complications	C108211	CPRD GOLD
49554	Type 1 diabetes mellitus with diabetic cataract	C10EF00	CPRD GOLD
49655	Type II diabetes mellitus with retinopathy	C10F611	CPRD GOLD
49869	Type 2 diabetes mellitus with arthropathy	C109G12	CPRD GOLD
49949	Unstable type I diabetes mellitus	C10E411	CPRD GOLD
50225	Type II diabetes mellitus with renal complications	C109011	CPRD GOLD
50429	Non-insulin-dependent diabetes mellitus with ophthalm comps	C109100	CPRD GOLD
50527	Type II diabetes mellitus with polyneuropathy	C10FB11	CPRD GOLD
50609	Pre-existing diabetes mellitus, non-insulin-dependent	L180600	CPRD GOLD
50813	Type II diabetes mellitus with mononeuropathy	C109A11	CPRD GOLD
51261	Insulin dependent diabetes mellitus	C10E.12	CPRD GOLD
51756	Type 2 diabetes mellitus with ketoacidotic coma	C10FP00	CPRD GOLD
51957	Type I diabetes mellitus with ulcer	C108511	CPRD GOLD
52041	O/E - left eye stable treated prolif diabetic retinopathy	2BBl.00	CPRD GOLD
52104	Insulin dependent diabetes mellitus with multiple complicatn	C108300	CPRD GOLD
52303	Non-insulin-dependent diabetes mellitus with renal comps	C109000	CPRD GOLD
52630	O/E - sight threatening diabetic retinopathy	2BBo.00	CPRD GOLD
53392	Type II diabetes mellitus without complication	C10F911	CPRD GOLD
54008	Type 1 diabetes mellitus with neuropathic arthropathy	C10EJ00	CPRD GOLD
54212	Non-insulin-dependent d m with peripheral angiopath	C109F00	CPRD GOLD
54600	Unstable insulin dependent diabetes mellitus	C10E412	CPRD GOLD
54899	Type II diabetes mellitus with peripheral angiopathy	C109F11	CPRD GOLD
55075	Type II diabetes mellitus with ulcer	C109411	CPRD GOLD
55239	Type 1 diabetes mellitus with gastroparesis	C10EQ00	CPRD GOLD
55842	Non-insulin-dependent diabetes mellitus with neuro comps	C109200	CPRD GOLD
56268	Type II diabetes mellitus with hypoglycaemic coma	C109D11	CPRD GOLD
56803	NIDDM with peripheral circulatory disorder	C107400	CPRD GOLD
57278	Type II diabetes mellitus with renal complications	C10F011	CPRD GOLD
57621	Insulin dependent diabetes mellitus with nephropathy	C108D00	CPRD GOLD
58604	Type II diabetes mellitus with retinopathy	C109611	CPRD GOLD
59253	Type 2 diabetes mellitus with arthropathy	C10FG00	CPRD GOLD
59365	Non-insulin dependent diabetes mellitus with nephropathy	C109C00	CPRD GOLD
59725	Type II diabetes mellitus with ophthalmic complications	C109111	CPRD GOLD
60107	Unstable type I diabetes mellitus	C108411	CPRD GOLD
60208	Type I diabetes mellitus with neuropathic arthropathy	C108J11	CPRD GOLD
60499	Insulin dependent diabetes mellitus with gangrene	C108600	CPRD GOLD
60699	Type 2 diabetes mellitus with peripheral angiopathy	C109F12	CPRD GOLD
60796	Type II diabetes mellitus with persistent proteinuria	C10FL11	CPRD GOLD
61071	Type 2 diabetes mellitus with hypoglycaemic coma	C109D12	CPRD GOLD
61344	Type I diabetes mellitus with renal complications	C108011	CPRD GOLD
61829	Type 1 diabetes mellitus with neurological complications	C108212	CPRD GOLD
62107	Type II diabetes mellitus with gangrene	C109511	CPRD GOLD
62146	Non-insulin-dependent diabetes mellitus with multiple comps	C109300	CPRD GOLD
62209	Type I diabetes mellitus with ketoacidosis	C10EM11	CPRD GOLD
62352	Type I diabetes mellitus with arthropathy	C108H11	CPRD GOLD
62613	Type I diabetes mellitus without complication	C10EA11	CPRD GOLD

62674	Type 2 diabetes mellitus with mononeuropathy	C10FA00	CPRD GOLD
63017	Type I diabetes mellitus maturity onset	C108911	CPRD GOLD
63690	Type 2 diabetes mellitus with gastroparesis	C10FR00	CPRD GOLD
64446	Insulin dependent diab mell with peripheral angiopathy	C108G00	CPRD GOLD
64571	Type II diabetes mellitus with nephropathy	C109C11	CPRD GOLD
64668	Insulin treated Type II diabetes mellitus	C10FJ11	CPRD GOLD
65267	Type 2 diabetes mellitus with multiple complications	C10F300	CPRD GOLD
65463	High risk non proliferative diabetic retinopathy	F420800	CPRD GOLD
65616	Insulin dependent diabetes mellitus with arthropathy	C108H00	CPRD GOLD
65704	Type 2 diabetes mellitus with ulcer	C109412	CPRD GOLD
66145	Type I diabetes mellitus with ketoacidotic coma	C10EN11	CPRD GOLD
66872	Type I diabetes mellitus with nephropathy	C108D11	CPRD GOLD
66965	Type 2 diabetes mellitus with neuropathic arthropathy	C109H12	CPRD GOLD
67905	Type II diabetes mellitus with neurological complications	C109211	CPRD GOLD
68105	Type 1 diabetes mellitus with mononeuropathy	C10EB00	CPRD GOLD
68390	Type 1 diabetes mellitus with ulcer	C108512	CPRD GOLD
69043	Dietary advice for type I diabetes	ZC2C900	CPRD GOLD
69278	Non-insulin depend diabetes mellitus with diabetic cataract	C109E00	CPRD GOLD
69676	Type 1 diabetes mellitus without complication	C10EA00	CPRD GOLD
69993	Type 1 diabetes mellitus with gangrene	C10E600	CPRD GOLD
70316	Type 2 diabetes mellitus with ophthalmic complications	C109112	CPRD GOLD
70766	Type 1 diabetes mellitus with hypoglycaemic coma	C108E12	CPRD GOLD
72320	Non-insulin dependent diabetes mellitus with mononeuropathy	C109A00	CPRD GOLD
72702	Insulin dependent diabetes mellitus - poor control	C10E812	CPRD GOLD
85991	Type II diabetes mellitus with persistent microalbuminuria	C10FM11	CPRD GOLD
91646	Type II diabetes mellitus with ulcer	C10F411	CPRD GOLD
91942	Type I diabetes mellitus with multiple complications	C10E311	CPRD GOLD
91943	Type I diabetes mellitus with polyneuropathy	C10EC11	CPRD GOLD
93468	Type 1 diabetes mellitus with peripheral angiopathy	C10EG00	CPRD GOLD
93727	Type II diabetes mellitus with diabetic cataract	C10FE11	CPRD GOLD
93875	Insulin dependent diabetes mellitus with retinopathy	C10E712	CPRD GOLD
93878	Type I diabetes mellitus with ulcer	C10E511	CPRD GOLD
95343	Type I diabetes mellitus with retinopathy	C10E711	CPRD GOLD
95351	Type II diabetes mellitus with mononeuropathy	C10FA11	CPRD GOLD
95992	Type I diabetes mellitus without complication	C108A11	CPRD GOLD
96235	Type I diabetes mellitus maturity onset	C10E911	CPRD GOLD
97446	Type 1 diabetes mellitus maturity onset	C108912	CPRD GOLD
97474	Unstable type 1 diabetes mellitus	C108412	CPRD GOLD
97849	Insulin dependent diabetes maturity onset	C10E912	CPRD GOLD
97894	Type I diabetes mellitus with exudative maculopathy	C10EP11	CPRD GOLD
98616	Type II diabetes mellitus with neurological complications	C10F211	CPRD GOLD
98704	Insulin dependent diabetes mellitus with ulcer	C10E512	CPRD GOLD
98723	Type II diabetes mellitus with hypoglycaemic coma	C10FD11	CPRD GOLD
99231	Type I diabetes mellitus with mononeuropathy	C108B11	CPRD GOLD
99311	Type I diabetes mellitus with ophthalmic complications	C10E111	CPRD GOLD
99716	Insulin dependent diabetes mellitus with hypoglycaemic coma	C10EE12	CPRD GOLD

100770	Insulin dependent diabetes mellitus with diabetic cataract	C10EF12	CPRD GOLD
100964	Type II diabetes mellitus with ophthalmic complications	C10F111	CPRD GOLD
101311	Insulin dependent diabetes mellitus with polyneuropathy	C10EC12	CPRD GOLD
101881	Impaired vision due to diabetic retinopathy	2BBr.00	CPRD GOLD
102112	Type I diabetes mellitus with gangrene	C10E611	CPRD GOLD
102163	Insulin dependent diabetes mellitus with nephropathy	C10ED12	CPRD GOLD
102201	Type II diabetes mellitus with nephropathy	C10FC11	CPRD GOLD
102620	Type I diabetes mellitus with persistent microalbuminuria	C10EL11	CPRD GOLD
102740	Type 1 diabetes mellitus with ophthalmic complications	C108112	CPRD GOLD
103902	Type II diabetes mellitus with arthropathy	C10FG11	CPRD GOLD
104323	Type II diabetes mellitus with gangrene	C10F511	CPRD GOLD
104453	Type 1 diabetic dietary review	66At011	CPRD GOLD
104639	Type II diabetes mellitus with peripheral angiopathy	C10FF11	CPRD GOLD
105337	Type I diabetes mellitus - poor control	C10E811	CPRD GOLD
105784	Type 2 diabetes mellitus without complication	C109912	CPRD GOLD
106061	Type II diabetes mellitus with ketoacidotic coma	C10FP11	CPRD GOLD
106528	Type II diabetes mellitus with ketoacidosis	C10FN11	CPRD GOLD
107701	Hyperosmolar non-ketotic state in type II diabetes mellitus	C10FK11	CPRD GOLD
107824	Type II diabetes mellitus in remission	C10P100	CPRD GOLD
108005	Type 2 diabetes mellitus with multiple complications	C109312	CPRD GOLD
108007	Type I diabetes mellitus with multiple complications	C108311	CPRD GOLD
108360	Type I diabetes mellitus in remission	C10P000	CPRD GOLD
108724	Type I diabetes mellitus with gastroparesis	C10EQ11	CPRD GOLD
109051	Insulin dependent diabetes mellitus with gangrene	C10E612	CPRD GOLD
109103	Type II diabetes mellitus without complication	C109911	CPRD GOLD
109197	Type II diabetes mellitus with neuropathic arthropathy	C10FH11	CPRD GOLD
109628	Type 1 diabetes mellitus in remission	C10P011	CPRD GOLD
109700	Conversion to non-insulin injectable medication	66AH300	CPRD GOLD
109837	Type I diabetes mellitus with renal complications	C10E011	CPRD GOLD
109865	Type 2 diabetes mellitus with polyneuropathy	C109B12	CPRD GOLD
110344	Diabetic on non-insulin injectable medication	66o2.00	CPRD GOLD
110379	Diabetic on oral treatment and glucagon-like peptide 1	66o5.00	CPRD GOLD
110400	Type 1 diabetes mellitus with diabetic cataract	C108F12	CPRD GOLD
110611	Type 2 diabetes mellitus in remission	C10P111	CPRD GOLD
111106	Type 1 diabetes mellitus without complication	C108A12	CPRD GOLD
111798	Type II diabetes mellitus with exudative maculopathy	C10FQ11	CPRD GOLD
113197	Type 1 diabetes mellitus with nephropathy	C108D12	CPRD GOLD
113495	Type II diabetes mellitus with multiple complications	C109311	CPRD GOLD
113609	Type II diabetes mellitus with gastroparesis	C10FR11	CPRD GOLD
113863	Type I diabetes mellitus with nephropathy	C10ED11	CPRD GOLD
113975	Type I diabetes mellitus with polyneuropathy	C108C11	CPRD GOLD
114401	Type 1 diabetes mellitus with gangrene	C108612	CPRD GOLD

Supplementary Table 16: Multiple sclerosis codes

medcodeid	description	Snomed_ct_code	Database
41398015	Multiple sclerosis	24700007	CPRD Aurum
297177019	Multiple sclerosis of the spinal cord	192927008	CPRD Aurum
297179016	Generalised multiple sclerosis	192928003	CPRD Aurum
297180018	Exacerbation of multiple sclerosis	192929006	CPRD Aurum
297181019	Multiple sclerosis NOS	24700007	CPRD Aurum
1223979019	Disseminated sclerosis	24700007	CPRD Aurum
2674605012	Secondary progressive multiple sclerosis	425500002	CPRD Aurum
2692565012	Primary progressive multiple sclerosis	428700003	CPRD Aurum
299241000000110	Multiple sclerosis review	198491000000106	CPRD Aurum
300141000000119	Multiple sclerosis care plan agreed	811881000000109	CPRD Aurum
315671000000110	Multiple sclerosis multidisciplinary review	205631000000104	CPRD Aurum
641211000000118	Benign multiple sclerosis	438511000	CPRD Aurum
641301000000114	Malignant multiple sclerosis	439567002	CPRD Aurum
695191000006119	Multiple sclerosis of the brain stem	192926004	CPRD Aurum
699671000000115	Management of multiple sclerosis in onset phase	367561000000107	CPRD Aurum
699731000000110	Management of multiple sclerosis in early disease phase	367591000000101	CPRD Aurum
699791000000111	Management of multiple sclerosis in stable disability phase	367621000000103	CPRD Aurum
699911000000117	Management of multiple sclerosis in palliative phase	367681000000102	CPRD Aurum
905781000006118	[RFC] Multiple sclerosis	905781000006102	CPRD Aurum
908811000006112	[RFC] Multiple sclerosis	908811000006108	CPRD Aurum
979461000006111	Spec serv for pat with multiple sclerosis - enh serv admin	166411000000100	CPRD Aurum
983261000006119	Multiple sclerosis - personal health plan	983261000006103	CPRD Aurum
983271000006114	Multiple sclerosis - multidisciplinary review	983271000006105	CPRD Aurum
983281000006112	Multiple sclerosis - review	983281000006108	CPRD Aurum
983291000006110	Multiple sclerosis - primary progressive	983291000006106	CPRD Aurum
983301000006111	Multiple sclerosis - secondary progressive	983301000006107	CPRD Aurum
983311000006114	Multiple sclerosis - relapsing remitting	983311000006105	CPRD Aurum
1148911000000110	Multiple sclerosis review declined	515931000000104	CPRD Aurum
1674121000000110	Multiple sclerosis monitoring administration	758981000000100	CPRD Aurum
1674381000000110	Multiple sclerosis monitoring first letter	759051000000102	CPRD Aurum
1674661000000110	Multiple sclerosis monitoring second letter	759091000000105	CPRD Aurum
1674971000000110	Multiple sclerosis monitoring telephone invitation	759131000000108	CPRD Aurum
1675051000000110	Multiple sclerosis monitoring third letter	759171000000105	CPRD Aurum
1682241000006110	Relapsing and remitting multiple sclerosis	426373005	CPRD Aurum
1747821000006110	Multiple sclerosis monitoring administration	1747821000006100	CPRD Aurum
1747831000006110	Multiple sclerosis monitoring first letter	1747831000006100	CPRD Aurum
1747841000006110	Multiple sclerosis monitoring second letter	1747841000006100	CPRD Aurum
1747851000006110	Multiple sclerosis monitoring third letter	1747851000006100	CPRD Aurum
1747861000006110	Multiple sclerosis monitoring telephone invite	1747861000006100	CPRD Aurum
2894411000006110	MS - Multiple sclerosis	24700007	CPRD Aurum
2894421000006110	DS - Disseminated sclerosis	24700007	CPRD Aurum
4769281000006110	Multiple sclerosis of the brainstem	192926004	CPRD Aurum
4769291000006110	Brain stem multiple sclerosis	192926004	CPRD Aurum
4769321000006110	Generalized multiple sclerosis	192928003	CPRD Aurum

4769341000006110	Multiple sclerosis (MS) exacerbation	192929006	CPRD Aurum
7045281000006110	Multiple sclerosis (MS) secondary progressive	425500002	CPRD Aurum
7058051000006110	Multiple sclerosis (MS) relapsing remitting	426373005	CPRD Aurum
7092351000006110	Multiple sclerosis (MS) primary progressive	428700003	CPRD Aurum
**medcode**	**description**	**readcode**	**database**
684	Multiple sclerosis	F20..00	CPRD GOLD
2298	Exacerbation of multiple sclerosis	F203.00	CPRD GOLD
3440	Disseminated sclerosis	F20..11	CPRD GOLD
20493	Multiple sclerosis NOS	F20z.00	CPRD GOLD
23730	Generalised multiple sclerosis	F202.00	CPRD GOLD
40344	Multiple sclerosis of the brain stem	F200.00	CPRD GOLD
43232	Multiple sclerosis multidisciplinary review	666B.00	CPRD GOLD
44985	Multiple sclerosis review	666A.00	CPRD GOLD
48527	Spec serv for pat with multiple sclerosis - enh serv admin	9kG..00	CPRD GOLD
50161	Multiple sclerosis care plan agreed	8CS1.00	CPRD GOLD
69886	Multiple sclerosis of the spinal cord	F201.00	CPRD GOLD
95864	Management of multiple sclerosis in stable disability phase	8Cc2.00	CPRD GOLD
95972	Relapsing and remitting multiple sclerosis	F207.00	CPRD GOLD
96246	Secondary progressive multiple sclerosis	F208.00	CPRD GOLD
96291	Benign multiple sclerosis	F204.00	CPRD GOLD
96607	Primary progressive multiple sclerosis	F206.00	CPRD GOLD
97487	Management of multiple sclerosis in early disease phase	8Cc1.00	CPRD GOLD
98521	Management of multiple sclerosis in palliative phase	8Cc4.00	CPRD GOLD
98835	Management of multiple sclerosis in onset phase	8Cc0.00	CPRD GOLD
100165	Kurtzke multiple sclerosis rating scale	ZRVE.00	CPRD GOLD
100503	Multiple sclerosis review declined	8IAb.00	CPRD GOLD
106307	Multiple sclerosis monitoring administration	9mD..00	CPRD GOLD
109049	Multiple sclerosis monitoring first letter	9mD0.00	CPRD GOLD
109302	Multiple sclerosis monitoring second letter	9mD1.00	CPRD GOLD
112194	Multiple sclerosis monitoring telephone invitation	9mD2.00	CPRD GOLD

Supplementary Table 17: Hormone Replacement Therapy (HRT) codes

prodcodeid	productname	Database
2173941000033118	adgyn combi tablets	CPRD Aurum
2173841000033114	adgyn estro 2mg tablets	CPRD Aurum
3290641000033118	angeliq 1mg/2mg tablets	CPRD Aurum
3908341000033113	bedol 2mg tablets	CPRD Aurum
285141000033112	climagest 1mg tablets	CPRD Aurum
285241000033117	climagest 2mg tablets	CPRD Aurum
287741000033116	climaval 1mg tablets	CPRD Aurum
287841000033114	climaval 2mg tablets	CPRD Aurum
285441000033116	climesse tablets	CPRD Aurum
3908241000033115	clinorette tablets	CPRD Aurum
374341000033111	conjugated oestrogens 1.25mg tablets	CPRD Aurum
3193041000033118	conjugated oestrogens 1.25mg tablets and norgestrel 150microgram tablets	CPRD Aurum
1594241000033112	conjugated oestrogens 2.5mg tablets	CPRD Aurum
3192841000033115	conjugated oestrogens 300microgram / medroxyprogesterone 1.5mg modified-release tablets	CPRD Aurum
4118041000033114	conjugated oestrogens 300microgram tablets	CPRD Aurum
12291041000033111	conjugated oestrogens 450microgram / bazedoxifene 20mg modified-release tablets	CPRD Aurum
3192741000033113	conjugated oestrogens 625microgram / medroxyprogesterone 5mg tablets	CPRD Aurum
374441000033117	conjugated oestrogens 625microgram tablets	CPRD Aurum
3192941000033111	conjugated oestrogens 625microgram tablets and norgestrel 150microgram tablets	CPRD Aurum
316441000033116	conjugated oestrogens 625micrograms/g vaginal cream	CPRD Aurum
393141000033117	cyclo-progynova 1mg tablets	CPRD Aurum
394941000033117	cyclo-progynova 2mg tablets	CPRD Aurum
2217041000033117	dermestril - septem 50 patches	CPRD Aurum
2217141000033118	dermestril - septem 75 patches	CPRD Aurum
432341000033111	dermestril 100 patches	CPRD Aurum
432441000033117	dermestril 25 patches	CPRD Aurum
432541000033116	dermestril 50 patches	CPRD Aurum
12291141000033110	duavive 0.45mg/20mg modified-release tablets	CPRD Aurum
508341000033112	elleste duet 1mg tablets	CPRD Aurum
508241000033119	elleste duet 2mg tablets	CPRD Aurum
508441000033118	elleste duet conti tablets	CPRD Aurum
508041000033110	elleste solo 1mg tablets	CPRD Aurum
508141000033114	elleste solo 2mg tablets	CPRD Aurum
509141000033115	elleste solo mx 40 transdermal patches	CPRD Aurum
509241000033110	elleste solo mx 80 transdermal patches	CPRD Aurum
545141000033113	estracombi tts patches	CPRD Aurum
546541000033110	estraderm mx 100 patches	CPRD Aurum
546641000033111	estraderm mx 25 patches	CPRD Aurum
546741000033119	estraderm mx 50 patches	CPRD Aurum
1798141000033113	estraderm mx 75 patches	CPRD Aurum

545241000033118	estraderm tts 100 patches	CPRD Aurum
545341000033111	estraderm tts 25 patches	CPRD Aurum
545441000033117	estraderm tts 50 patches	CPRD Aurum
3097641000033113	estradiol 0.06% gel (750microgram per actuation)	CPRD Aurum
13451141000033114	estradiol 1.53mg/dose transdermal spray	CPRD Aurum
8348341000033114	estradiol 1.5mg / nomegestrol 2.5mg tablets	CPRD Aurum
3097741000033116	estradiol 100mg implant	CPRD Aurum
3098341000033118	estradiol 100micrograms/24hours transdermal patches	CPRD Aurum
6002841000033118	estradiol 10microgram pessaries	CPRD Aurum
3290541000033119	estradiol 1mg / drospirenone 2mg tablets	CPRD Aurum
3192241000033119	estradiol 1mg / dydrogesterone 5mg tablets	CPRD Aurum
3192641000033116	estradiol 1mg / norethisterone acetate 500microgram tablets	CPRD Aurum
13910141000033113	estradiol 1mg / progesterone 100mg capsules	CPRD Aurum
3099541000033118	estradiol 1mg gel sachets	CPRD Aurum
3098041000033115	estradiol 1mg tablets	CPRD Aurum
3097841000033114	estradiol 25mg implant	CPRD Aurum
3099341000033113	estradiol 25microgram pessaries	CPRD Aurum
3098441000033112	estradiol 25micrograms/24hours transdermal patches	CPRD Aurum
3191741000033119	estradiol 2mg / norethisterone acetate 1mg tablets	CPRD Aurum
3098141000033116	estradiol 2mg tablets	CPRD Aurum
3225741000033112	estradiol 37.5micrograms/24hours transdermal patches	CPRD Aurum
3098541000033113	estradiol 40micrograms/24hours transdermal patches	CPRD Aurum
3192041000033110	estradiol 40micrograms/24hours transdermal patches and dydrogesterone 10mg tablets	CPRD Aurum
3099641000033117	estradiol 500microgram gel sachets	CPRD Aurum
8129141000033112	estradiol 500micrograms / dydrogesterone 2.5mg tablets	CPRD Aurum
3097941000033118	estradiol 50mg implant	CPRD Aurum
3194641000033114	estradiol 50micrograms/24hours / levonorgestrel 7micrograms/24hours transdermal patches	CPRD Aurum
3191841000033112	estradiol 50micrograms/24hours / norethisterone 170micrograms/24hours transdermal patches	CPRD Aurum
3098641000033114	estradiol 50micrograms/24hours transdermal patches	CPRD Aurum
3191941000033116	estradiol 50micrograms/24hours transdermal patches and norethisterone 1mg tablets	CPRD Aurum
3099841000033116	estradiol 7.5micrograms/24hours vaginal delivery system	CPRD Aurum
3098741000033117	estradiol 75micrograms/24hours transdermal patches	CPRD Aurum
3098841000033110	estradiol 80micrograms/24hours transdermal patches	CPRD Aurum
3192141000033114	estradiol 80micrograms/24hours transdermal patches and dydrogesterone 10mg tablets	CPRD Aurum
3099441000033119	estradiol acetate 1.25mg vaginal ring	CPRD Aurum
3192341000033112	estradiol valerate 1mg / medroxyprogesterone 2.5mg tablets	CPRD Aurum
3192441000033118	estradiol valerate 1mg / medroxyprogesterone 5mg tablets	CPRD Aurum
3099941000033112	estradiol valerate 1mg tablets	CPRD Aurum
3192541000033117	estradiol valerate 2mg / medroxyprogesterone 5mg tablets	CPRD Aurum
3191541000033110	estradiol valerate 2mg / norethisterone 700microgram tablets	CPRD Aurum
3100041000033111	estradiol valerate 2mg tablets	CPRD Aurum
3226241000033113	estradot 100micrograms/24hours patches	CPRD Aurum

3225841000033119	estradot 25micrograms/24hours patches	CPRD Aurum
3225941000033110	estradot 37.5micrograms/24hours patches	CPRD Aurum
3226041000033117	estradot 50micrograms/24hours patches	CPRD Aurum
3226141000033118	estradot 75micrograms/24hours patches	CPRD Aurum
546441000033114	estrapak 50	CPRD Aurum
546841000033112	estring 7.5micrograms/24hours vaginal delivery system	CPRD Aurum
3100741000033114	estriol 0.01% cream with applicator	CPRD Aurum
3100541000033118	estriol 0.1% cream	CPRD Aurum
3100441000033119	estriol 1mg tablets	CPRD Aurum
13130441000033110	estriol 30microgram pessaries	CPRD Aurum
3100841000033116	estriol 500microgram pessaries	CPRD Aurum
13010741000033115	estriol 50micrograms/g vaginal gel with applicator	CPRD Aurum
3090041000033118	ethinylestradiol 10microgram tablets	CPRD Aurum
3090241000033114	ethinylestradiol 1mg tablets	CPRD Aurum
12420841000033116	ethinylestradiol 2.7mg / etonogestrel 11.7mg vaginal delivery system	CPRD Aurum
3193941000033117	ethinylestradiol 20microgram / desogestrel 150microgram tablets	CPRD Aurum
10599741000033112	ethinylestradiol 20microgram / drospirenone 3mg tablets	CPRD Aurum
3194541000033113	ethinylestradiol 20microgram / gestodene 75microgram tablets	CPRD Aurum
3193641000033112	ethinylestradiol 20microgram / norethisterone acetate 1mg tablets	CPRD Aurum
3090341000033116	ethinylestradiol 2microgram tablets	CPRD Aurum
3193841000033113	ethinylestradiol 30microgram / desogestrel 150microgram tablets	CPRD Aurum
3194341000033118	ethinylestradiol 30microgram / drospirenone 3mg tablets	CPRD Aurum
3193541000033111	ethinylestradiol 30microgram / gestodene 75microgram tablets	CPRD Aurum
3194041000033115	ethinylestradiol 30microgram / levonorgestrel 150microgram tablets	CPRD Aurum
3193741000033115	ethinylestradiol 30microgram / norethisterone acetate 1.5mg tablets	CPRD Aurum
3194741000033117	ethinylestradiol 33.9micrograms/24hours / norelgestromin 203micrograms/24hours transdermal patches	CPRD Aurum
3194141000033116	ethinylestradiol 35microgram / norethisterone 1mg tablets	CPRD Aurum
3193141000033119	ethinylestradiol 35microgram / norethisterone 500microgram tablets	CPRD Aurum
3193241000033114	ethinylestradiol 35microgram / norgestimate 250microgram tablets	CPRD Aurum
13829441000033113	ethinylestradiol 50microgram gastro-resistant tablets	CPRD Aurum
3090141000033119	ethinylestradiol 50microgram tablets	CPRD Aurum
1609141000033111	evista 60mg tablets	CPRD Aurum
558641000033112	evorel 100 patches	CPRD Aurum
558741000033115	evorel 25 patches	CPRD Aurum
558541000033111	evorel 50 patches	CPRD Aurum
558841000033113	evorel 75 patches	CPRD Aurum
559041000033114	evorel conti patches	CPRD Aurum
557941000033116	evorel pak	CPRD Aurum
558941000033117	evorel sequi patches	CPRD Aurum
572541000033110	femapak 40	CPRD Aurum
572441000033114	femapak 80	CPRD Aurum
575541000033115	fematrix 40 patches	CPRD Aurum
575341000033110	fematrix 80 patches	CPRD Aurum

572341000033115	femoston 1/10mg tablets	CPRD Aurum
571841000033115	femoston 2/10mg tablets	CPRD Aurum
571941000033111	femoston 2/20mg tablets	CPRD Aurum
8129241000033117	femoston-conti 0.5mg/2.5mg tablets	CPRD Aurum
1922141000033113	femoston-conti 1mg/5mg tablets	CPRD Aurum
575641000033119	femseven 100 patches	CPRD Aurum
575441000033116	femseven 50 patches	CPRD Aurum
575741000033111	femseven 75 patches	CPRD Aurum
2846541000033110	femseven conti patches	CPRD Aurum
2801741000033117	femseven sequi patches	CPRD Aurum
2950941000033111	femtab 1mg tablets	CPRD Aurum
2951041000033118	femtab 2mg tablets	CPRD Aurum
2951241000033114	femtab continuous tablets	CPRD Aurum
2951141000033119	femtab sequi tablets	CPRD Aurum
709541000033113	hormonin tablets	CPRD Aurum
2215241000033115	indivina 1mg/2.5mg tablets	CPRD Aurum
2215341000033113	indivina 1mg/5mg tablets	CPRD Aurum
2215441000033119	indivina 2mg/5mg tablets	CPRD Aurum
805541000033114	kliofem tablets	CPRD Aurum
1625541000033112	kliovance tablets	CPRD Aurum
3193441000033110	levonorgestrel 250microgram / ethinylestradiol 30microgram tablets	CPRD Aurum
838241000033112	livial 2.5mg tablets	CPRD Aurum
905341000033110	menorest 37.5 patches	CPRD Aurum
905441000033116	menorest 50 patches	CPRD Aurum
905541000033115	menorest 75 patches	CPRD Aurum
2738741000033116	novofem tablets	CPRD Aurum
1798241000033118	nuvelle continuous tablets	CPRD Aurum
993241000033115	nuvelle tablets	CPRD Aurum
994041000033114	nuvelle ts patches	CPRD Aurum
999041000033113	oestrogel pump-pack 0.06% gel	CPRD Aurum
14041141000033115	ovestin 1mg cream	CPRD Aurum
1021441000033115	ovestin 1mg cream	CPRD Aurum
1021841000033117	ovestin 1mg tablets	CPRD Aurum
4118141000033113	premarin 0.3mg tablets	CPRD Aurum
1132441000033116	premarin 0.625mg tablets	CPRD Aurum
1112641000033111	premarin 0.625mg/g vaginal cream	CPRD Aurum
1132341000033110	premarin 1.25mg tablets	CPRD Aurum
1140041000033113	premarin 2.5mg tablets	CPRD Aurum
1136041000033114	premique 0.625mg/5mg tablets	CPRD Aurum
1136141000033113	premique cycle 0.625mg/10mg tablets	CPRD Aurum
3076041000033112	premique low dose 0.3mg/1.5mg modified-release tablets	CPRD Aurum
1132541000033115	prempak-c 0.625mg/0.15mg tablets	CPRD Aurum
1132641000033119	prempak-c 1.25mg/0.15mg tablets	CPRD Aurum

1134041000033118	progynova 1mg tablets	CPRD Aurum
1134141000033119	progynova 2mg tablets	CPRD Aurum
2739441000033118	progynova ts 100micrograms/24hours transdermal patches	CPRD Aurum
2739341000033112	progynova ts 50micrograms/24hours transdermal patches	CPRD Aurum
14041041000033119	sandrena 1mg gel sachets	CPRD Aurum
1239041000033116	sandrena 1mg gel sachets	CPRD Aurum
1239141000033117	sandrena 500microgram gel sachets	CPRD Aurum
1444741000033117	tibolone 2.5mg tablets	CPRD Aurum
1468741000033119	tridestra tablets	CPRD Aurum
1468441000033114	trisequens forte tablets	CPRD Aurum
1455141000033118	trisequens tablets	CPRD Aurum
6002941000033114	vagifem 10microgram vaginal tablets	CPRD Aurum
1505641000033111	vagifem 25microgram vaginal tablets	CPRD Aurum
1563941000033116	zumenon 1mg tablets	CPRD Aurum
1563841000033112	zumenon 2mg tablets	CPRD Aurum
**prodcode**	**productname**	**Database**
6041	estradiol 0.06% gel	CPRD GOLD
17475	adgyn combi tablets 2mg + (2mg + 1mg) [strakan]	CPRD GOLD
20888	adgyn estro 2mg tablets (kyowa kirin ltd)	CPRD GOLD
16493	angeliq tablets (bayer plc)	CPRD GOLD
35958	bedol 2mg tablets (resource medical uk ltd)	CPRD GOLD
390	climagest 1mg tablets (novartis pharmaceuticals uk ltd)	CPRD GOLD
50200	climagest 1mg tablets (sigma pharmaceuticals plc)	CPRD GOLD
66404	climagest 1mg tablets (waymade healthcare plc)	CPRD GOLD
2111	climagest 2mg tablets (novartis pharmaceuticals uk ltd)	CPRD GOLD
60637	climaval 1mg tablets (lexon (uk) ltd)	CPRD GOLD
1873	climaval 1mg tablets (novartis pharmaceuticals uk ltd)	CPRD GOLD
51546	climaval 2mg tablets (lexon (uk) ltd)	CPRD GOLD
2262	climaval 2mg tablets (novartis pharmaceuticals uk ltd)	CPRD GOLD
64995	climesse tablets (lexon (uk) ltd)	CPRD GOLD
2766	climesse tablets (novartis pharmaceuticals uk ltd)	CPRD GOLD
28230	clinorette tablets (resource medical uk ltd)	CPRD GOLD
19429	conjugat oestrogen equi and (conjugat oestrogen equi with medroxyprogesterone acetate 625 micrograms with (625 microgram with 10 mg)	CPRD GOLD
3960	conjugated oestrogens 1.25mg tablets	CPRD GOLD
12970	conjugated oestrogens 1.25mg tablets and norgestrel 150microgram tablets	CPRD GOLD
11934	conjugated oestrogens 300microgram / medroxyprogesterone 1.5mg modified-release tablets	CPRD GOLD
35718	conjugated oestrogens 300microgram tablets	CPRD GOLD
69137	conjugated oestrogens 450microgram / bazedoxifene 20mg modified-release tablets	CPRD GOLD
11859	conjugated oestrogens 625microgram / medroxyprogesterone 5mg tablets	CPRD GOLD
3423	conjugated oestrogens 625microgram tablets	CPRD GOLD
22741	conjugated oestrogens 625microgram tablets and norgestrel 150microgram tablets	CPRD GOLD
932	conjugated oestrogens 625micrograms/g vaginal cream	CPRD GOLD

34174	conjugated oestrogens equine with medroxyprogesterone acetate 625micrograms with 10mg tablets	CPRD GOLD
3036	cyclo-progynova 2mg tablets (meda pharmaceuticals ltd)	CPRD GOLD
20895	dermestril - septem 50 patches (kyowa kirin ltd)	CPRD GOLD
15880	dermestril - septem 75 patches (kyowa kirin ltd)	CPRD GOLD
15194	dermestril 100 patches (kyowa kirin ltd)	CPRD GOLD
14580	dermestril 25 patches (kyowa kirin ltd)	CPRD GOLD
15869	dermestril 50 patches (kyowa kirin ltd)	CPRD GOLD
32930	dermestril septem 25microgram/24hr transdermal patch (strakan ltd)	CPRD GOLD
8777	dienestrol 1mg tablets	CPRD GOLD
29573	dienestrol 5mg tablets	CPRD GOLD
69756	duavive 0.45mg/20mg modified-release tablets (merck sharp & dohme ltd)	CPRD GOLD
3002	elleste duet 1mg tablets (meda pharmaceuticals ltd)	CPRD GOLD
4207	elleste duet 2mg tablets (meda pharmaceuticals ltd)	CPRD GOLD
64580	elleste duet conti tablets (mawdsley-brooks & company ltd)	CPRD GOLD
4393	elleste duet conti tablets (meda pharmaceuticals ltd)	CPRD GOLD
4328	elleste solo 1mg tablets (meda pharmaceuticals ltd)	CPRD GOLD
67666	elleste solo 2mg tablets (mawdsley-brooks & company ltd)	CPRD GOLD
4909	elleste solo 2mg tablets (meda pharmaceuticals ltd)	CPRD GOLD
5755	elleste solo mx 40 transdermal patches (meda pharmaceuticals ltd)	CPRD GOLD
9649	elleste solo mx 80 transdermal patches (meda pharmaceuticals ltd)	CPRD GOLD
67533	estracombi tts patches (de pharmaceuticals)	CPRD GOLD
1183	estracombi tts patches (novartis pharmaceuticals uk ltd)	CPRD GOLD
4882	estraderm mx 100 patches (merus labs luxco s.a r.l.)	CPRD GOLD
52480	estraderm mx 25 patches (lexon (uk) ltd)	CPRD GOLD
3387	estraderm mx 25 patches (merus labs luxco s.a r.l.)	CPRD GOLD
51978	estraderm mx 25 patches (stephar (u.k.) ltd)	CPRD GOLD
3040	estraderm mx 50 patches (merus labs luxco s.a r.l.)	CPRD GOLD
4977	estraderm mx 75 patches (merus labs luxco s.a r.l.)	CPRD GOLD
206	estraderm tts 100 patches (novartis pharmaceuticals uk ltd)	CPRD GOLD
49694	estraderm tts 25 patches (de pharmaceuticals)	CPRD GOLD
988	estraderm tts 25 patches (novartis pharmaceuticals uk ltd)	CPRD GOLD
986	estraderm tts 50 patches (novartis pharmaceuticals uk ltd)	CPRD GOLD
67806	estraderm tts 50 patches (sigma pharmaceuticals plc)	CPRD GOLD
57514	estraderm tts 50 patches (waymade healthcare plc)	CPRD GOLD
7242	estradiol 0.1% gel	CPRD GOLD
44461	estradiol 10microgram pessaries	CPRD GOLD
22975	estradiol 1mg / drospirenone 2mg tablets	CPRD GOLD
40848	estradiol 1mg gel sachets	CPRD GOLD
7615	estradiol 25microgram pessaries	CPRD GOLD
17529	estradiol 2mg vaginal ring	CPRD GOLD
23152	estradiol 40micrograms/24hours transdermal patches and dydrogesterone 10mg tablets	CPRD GOLD
40836	estradiol 500microgram gel sachets	CPRD GOLD
55551	estradiol 500micrograms / dydrogesterone 2.5mg tablets	CPRD GOLD

19135	estradiol 50mg implant (merck sharp & dohme ltd)	CPRD GOLD
19974	estradiol 50micrograms/24hours / levonorgestrel 7micrograms/24hours transdermal patches	CPRD GOLD
22372	estradiol 80micrograms/24hours transdermal patches and dydrogesterone 10mg tablets	CPRD GOLD
11157	estradiol and (estradiol with dydrogesterone) tablets 1mg + (1mg + 10mg)	CPRD GOLD
12907	estradiol and (estradiol with dydrogesterone) tablets 1mg with 5mg	CPRD GOLD
11511	estradiol and (estradiol with dydrogesterone) tablets 2mg + (2mg + 10 mg)	CPRD GOLD
11363	estradiol and (estradiol with dydrogesterone) tablets 2mg + (2mg + 20mg)	CPRD GOLD
19986	estradiol and (estradiol with levonorgestrel) 50mcg/24hrs with (50mcg+10mcg/24hrs) once weekly patch	CPRD GOLD
13945	estradiol and (estradiol with levonorgestrel) 80mcg/24hrs with (50mcg+20mcg/24hr) twice weekly patch	CPRD GOLD
14596	estradiol and (estradiol with norethisterone) and (estradiol) triphasic forte tablets 4mg + (4mg + 1	CPRD GOLD
14569	estradiol and (estradiol with norethisterone) and (estradiol) triphasic tablets 2mg + (2mg + 1mg) +	CPRD GOLD
24764	estradiol and (estradiol with norethisterone) tablets 2mg + (2mg + 1mg)	CPRD GOLD
4511	estradiol implant 100mg	CPRD GOLD
21757	estradiol implant 100mg [organon]	CPRD GOLD
8837	estradiol implant 25mg	CPRD GOLD
2977	estradiol implant 50mg	CPRD GOLD
29449	estradiol injection 1mg/ml	CPRD GOLD
31330	estradiol injection 5mg/ml	CPRD GOLD
16437	estradiol once weekly patch 100mcg/24 hour (7.6mg/unit)	CPRD GOLD
22335	estradiol once weekly patch 100mcg/24 hours (3.0mg/unit)	CPRD GOLD
35742	estradiol once weekly patch 100micrograms/24 hours	CPRD GOLD
11430	estradiol once weekly patch 25micrograms/24 hours	CPRD GOLD
18009	estradiol once weekly patch 50mcg/24hours (3.8 mg/unit)	CPRD GOLD
20155	estradiol once weekly patch 50micrograms/24 hours	CPRD GOLD
11375	estradiol once weekly patch 50micrograms/24 hours(1.5mg/unit)	CPRD GOLD
6563	estradiol once weekly patch 75micrograms/24 hours	CPRD GOLD
42515	estradiol patch 40micrograms/24 hours (1.25mg/unit)	CPRD GOLD
4956	estradiol tablets 1mg	CPRD GOLD
7381	estradiol tablets 2mg	CPRD GOLD
37037	estradiol transdermal patch 100micrograms/24 hours	CPRD GOLD
37692	estradiol transdermal patch 25micrograms/24 hours	CPRD GOLD
37697	estradiol transdermal patch 50micrograms/24 hours	CPRD GOLD
37033	estradiol transdermal patch 75micrograms/24 hours	CPRD GOLD
26049	estradiol twice weekly patch 100 micrograms/24 hours (1.56mg/unit)	CPRD GOLD
6059	estradiol twice weekly patch 100mcg/24 hours (6.4mg/unit)	CPRD GOLD
18218	estradiol twice weekly patch 100micrograms/24 hours	CPRD GOLD
5759	estradiol twice weekly patch 100micrograms/24 hours (8mg/unit)	CPRD GOLD
12773	estradiol twice weekly patch 100micrograms/24hrs (3.0mg/unit)	CPRD GOLD
15298	estradiol twice weekly patch 25 micrograms/24 hours (0.39mg/unit)	CPRD GOLD
5100	estradiol twice weekly patch 25mcg/24 hours (1.6mg/unit)	CPRD GOLD
15328	estradiol twice weekly patch 25micrograms/24 hours	CPRD GOLD
11672	estradiol twice weekly patch 25micrograms/24 hours (0.75mg/unit)	CPRD GOLD

6601	estradiol twice weekly patch 25micrograms/24 hours (2mg/unit)	CPRD GOLD
5343	estradiol twice weekly patch 25micrograms/24 hours (2mg/unit)	CPRD GOLD
14234	estradiol twice weekly patch 37.5micrograms/24 hours	CPRD GOLD
15100	estradiol twice weekly patch 37.5micrograms/24 hours (3.29mg/unit)	CPRD GOLD
6793	estradiol twice weekly patch 40micrograms/24 hours	CPRD GOLD
11882	estradiol twice weekly patch 50 micrograms/24 hours (0.78mg/unit)	CPRD GOLD
5005	estradiol twice weekly patch 50mcg/24 hours (3.2mg/unit)	CPRD GOLD
18901	estradiol twice weekly patch 50micrograms/24 hours	CPRD GOLD
19145	estradiol twice weekly patch 50micrograms/24 hours (4.33mg/unit)	CPRD GOLD
9268	estradiol twice weekly patch 50micrograms/24 hours (4mg/unit)	CPRD GOLD
14792	estradiol twice weekly patch 50micrograms/24 hours(1.5mg/unit)	CPRD GOLD
20135	estradiol twice weekly patch 75 micrograms/24 hours (1.17mg/unit)	CPRD GOLD
18600	estradiol twice weekly patch 75micrograms/24 hours	CPRD GOLD
18502	estradiol twice weekly patch 75micrograms/24 hours (2.25mg/unit)	CPRD GOLD
4721	estradiol twice weekly patch 75micrograms/24 hours(4.8mg/unit)	CPRD GOLD
18437	estradiol twice weekly patch 75micrograms/24hours (6.57mg/unit)	CPRD GOLD
18383	estradiol twice weekly patch 80micrograms/24 hours	CPRD GOLD
16367	estradiol valerate 1mg / medroxyprogesterone 2.5mg tablets	CPRD GOLD
19432	estradiol valerate 1mg / medroxyprogesterone 5mg tablets	CPRD GOLD
2457	estradiol valerate 1mg tablets	CPRD GOLD
16392	estradiol valerate 2mg / medroxyprogesterone 5mg tablets	CPRD GOLD
67659	estradiol valerate 2mg tablets (de pharmaceuticals)	CPRD GOLD
11642	estradiol valerate and (estradiol valerate with levonorgestrel) 1mg with (1mg with 250micrograms) tablets	CPRD GOLD
11297	estradiol valerate and (estradiol valerate with levonorgestrel) 2mg with (2mg with 75micrograms) tablets	CPRD GOLD
18167	estradiol valerate and (estradiol valerate with medroxyprogesterone acetate) with placebo 2mg with (2mg with 20mg) with 7 placebo tablets	CPRD GOLD
9413	estradiol valerate and (estradiol valerate with norethisterone) tablets 1mg + (1mg + 1mg)	CPRD GOLD
12713	estradiol valerate and (estradiol valerate with norethisterone) tablets 2mg + (2mg + 1 mg)	CPRD GOLD
18530	estradiol valerate and (estradiol valerate with norgestrel) 2mg with (2mg with 500micrograms) tablets	CPRD GOLD
7001	estradiol valerate tablets 2mg	CPRD GOLD
12661	estradiol valerate with medroxyprogesterone acetate tablets	CPRD GOLD
24917	estradiol valerate with norethisterone (continuous combined) tablets 2mg + 0.7mg	CPRD GOLD
54456	estradiol valerate with norgestrel 2mg+500micrograms tablets	CPRD GOLD
14027	estradiol with (estradiol with norethisterone acetate) 1mg with (1mg with 1mg) tablets	CPRD GOLD
16997	estradiol with (estradiol with norethisterone acetate) 2mg with (2mg with 1 mg) tablets	CPRD GOLD
14812	estradiol with (estradiol with norethisterone acetate) 50mcg/24 hr with (50mcg+170mcg/24 hr) twice weekly patch	CPRD GOLD
9976	estradiol with (estradiol with norethisterone acetate) 50mcg/24 hr with (50mcg+250mcg/24 hr) twice weekly patch	CPRD GOLD
41390	estradiol with (estradiol with norethisterone acetate) twice weekly patch	CPRD GOLD
63654	estradiol with dydrogesterone 1mg +10mg tablets	CPRD GOLD
12953	estradiol with dydrogesterone tablets 1mg + 5mg	CPRD GOLD
6603	estradiol with estrone and estriol tablets	CPRD GOLD
12788	estradiol with norethisterone acetate (continuous combined) tablets 1mg + 0.5mg	CPRD GOLD

12807	estradiol with norethisterone acetate (continuous combined) tablets 2mg + 0.7mg	CPRD GOLD
5012	estradiol with norethisterone acetate (continuous combined) tablets 2mg + 1mg	CPRD GOLD
13990	estradiol with norethisterone acetate patch and tablets 50mcg/24hours(4mg/unit) + 1mg	CPRD GOLD
14950	estradiol with norethisterone acetate twice weekly patch (50mcg + 170mcg)/24 hr	CPRD GOLD
8162	estradiol with norethisterone patch and tablets 50mcg/24hours(3.2mg/unit) + 1mg	CPRD GOLD
10096	estradot 100micrograms/24hours patches (novartis pharmaceuticals uk ltd)	CPRD GOLD
10126	estradot 25micrograms/24hours patches (novartis pharmaceuticals uk ltd)	CPRD GOLD
19487	estradot 37.5micrograms/24hours patches (novartis pharmaceuticals uk ltd)	CPRD GOLD
51201	estradot 50micrograms/24hours patches (necessity supplies ltd)	CPRD GOLD
10180	estradot 50micrograms/24hours patches (novartis pharmaceuticals uk ltd)	CPRD GOLD
52174	estradot 50micrograms/24hours patches (sigma pharmaceuticals plc)	CPRD GOLD
64735	estradot 50micrograms/24hours patches (waymade healthcare plc)	CPRD GOLD
10076	estradot 75micrograms/24hours patches (novartis pharmaceuticals uk ltd)	CPRD GOLD
1014	estrapak 50 patch and tablets 50mcg/24hours(4mg/unit) + 1mg [nov/ciba]	CPRD GOLD
9181	estring 2mg vaginal ring (pfizer ltd)	CPRD GOLD
10593	estriol tablets 1mg	CPRD GOLD
18311	estriol tablets 250micrograms	CPRD GOLD
67141	ethinylestradiol 2microgram tablets (ucb pharma ltd)	CPRD GOLD
5931	evista 60mg tablets (daiichi sankyo uk ltd)	CPRD GOLD
67320	evista 60mg tablets (mawdsley-brooks & company ltd)	CPRD GOLD
2397	evorel -100 100microgram/24 hr (6.4mg/unit) transdermal patch (janssen-cilag ltd)	CPRD GOLD
2141	evorel -25 25microgram/24hr (1.6mg/unit) transdermal patch (janssen-cilag ltd)	CPRD GOLD
1488	evorel -50 50microgram/24hr (3.2mg/unit) transdermal patch (janssen-cilag ltd)	CPRD GOLD
1489	evorel -75 75microgram/24 hr(4.8mg/unit) transdermal patch (janssen-cilag ltd)	CPRD GOLD
38965	evorel 100 patches (janssen-cilag ltd)	CPRD GOLD
38935	evorel 25 patches (janssen-cilag ltd)	CPRD GOLD
38932	evorel 50 patches (janssen-cilag ltd)	CPRD GOLD
66575	evorel 50 patches (lexon (uk) ltd)	CPRD GOLD
38940	evorel 75 patches (janssen-cilag ltd)	CPRD GOLD
52441	evorel conti patches (de pharmaceuticals)	CPRD GOLD
2729	evorel conti patches (janssen-cilag ltd)	CPRD GOLD
51688	evorel conti patches (lexon (uk) ltd)	CPRD GOLD
56480	evorel conti patches (waymade healthcare plc)	CPRD GOLD
2728	evorel pak treatment pack 50mcg/24hours(3.2mg/unit) + 1mg [janssen]	CPRD GOLD
9234	evorel sequi 50microgram/24hr (3.2mg/unit) transdermal patch (janssen-cilag ltd)	CPRD GOLD
4244	evorel sequi patches (janssen-cilag ltd)	CPRD GOLD
69377	evorel sequi patches (lexon (uk) ltd)	CPRD GOLD
3131	femapak 40 (abbott healthcare products ltd)	CPRD GOLD
9684	femapak 80 (abbott healthcare products ltd)	CPRD GOLD
5292	fematrix 40 patches (abbott healthcare products ltd)	CPRD GOLD
9047	fematrix 80 patches (abbott healthcare products ltd)	CPRD GOLD

3530	femoston 1/10mg tablets (mylan ltd)	CPRD GOLD
67774	femoston 2/10mg tablets (mawdsley-brooks & company ltd)	CPRD GOLD
3553	femoston 2/10mg tablets (mylan ltd)	CPRD GOLD
65810	femoston 2/10mg tablets (sigma pharmaceuticals plc)	CPRD GOLD
8770	femoston tablets 2mg + 20mg [abbott]	CPRD GOLD
49985	femoston-conti 0.5mg/2.5mg tablets (mylan ltd)	CPRD GOLD
69143	femoston-conti 1mg/5mg tablets (mawdsley-brooks & company ltd)	CPRD GOLD
5195	femoston-conti 1mg/5mg tablets (mylan ltd)	CPRD GOLD
52306	femoston-conti 1mg/5mg tablets (stephar (u.k.) ltd)	CPRD GOLD
6177	femseven 100 patches (teva uk ltd)	CPRD GOLD
2140	femseven 50 patches (teva uk ltd)	CPRD GOLD
6082	femseven 75 patches (teva uk ltd)	CPRD GOLD
49757	femseven conti patches (lexon (uk) ltd)	CPRD GOLD
6545	femseven conti patches (teva uk ltd)	CPRD GOLD
9905	femseven sequi patches (teva uk ltd)	CPRD GOLD
7251	femtab 1mg tablets (merck serono ltd)	CPRD GOLD
6538	femtab 2mg tablets (merck serono ltd)	CPRD GOLD
16443	femtab continuous tablets (merck serono ltd)	CPRD GOLD
58575	generic climagest 1mg tablets	CPRD GOLD
67760	generic cyclo-progynova 2mg tablets	CPRD GOLD
56056	generic elleste duet 1mg tablets	CPRD GOLD
58035	generic elleste duet 2mg tablets	CPRD GOLD
66434	generic evorel sequi transdermal patches	CPRD GOLD
56081	generic femoston 1/10mg tablets	CPRD GOLD
58516	generic femoston 2/10mg tablets	CPRD GOLD
62530	generic tridestra tablets	CPRD GOLD
65009	generic trisequens tablets	CPRD GOLD
1922	hormonin tablet (shire pharmaceuticals ltd)	CPRD GOLD
39622	hormonin tablets (amco)	CPRD GOLD
17293	improvera 1.5mg+10mg tablet (pharmacia ltd)	CPRD GOLD
7175	indivina 1mg/2.5mg tablets (orion pharma (uk) ltd)	CPRD GOLD
67635	indivina 1mg/5mg tablets (lexon (uk) ltd)	CPRD GOLD
6861	indivina 1mg/5mg tablets (orion pharma (uk) ltd)	CPRD GOLD
9671	indivina 2mg/5mg tablets (orion pharma (uk) ltd)	CPRD GOLD
1268	kliofem tablets (novo nordisk ltd)	CPRD GOLD
2115	kliovance tablets (novo nordisk ltd)	CPRD GOLD
67240	livial 2.5mg tablets (mawdsley-brooks & company ltd)	CPRD GOLD
2209	livial 2.5mg tablets (merck sharp & dohme ltd)	CPRD GOLD
57615	livial 2.5mg tablets (waymade healthcare plc)	CPRD GOLD
1774	menophase tablet (pharmacia ltd)	CPRD GOLD
10967	menorest 37.5 patches (novartis pharmaceuticals uk ltd)	CPRD GOLD
10946	menorest 50 patches (novartis pharmaceuticals uk ltd)	CPRD GOLD
13582	menorest 75 patches (novartis pharmaceuticals uk ltd)	CPRD GOLD

28811	mestranol with norethisterone tablet	CPRD GOLD
20393	mixogen tablet (organon laboratories ltd)	CPRD GOLD
9235	norgestrel and conjugated oestrogens (equine) 150micrograms + 1.25mg tablet	CPRD GOLD
9224	norgestrel and conjugated oestrogens (equine) 150micrograms + 625micrograms tablet	CPRD GOLD
13146	novofem tablets (novo nordisk ltd)	CPRD GOLD
6030	nuvelle continuous tablets (bayer plc)	CPRD GOLD
52813	nuvelle ts phase i patches (bayer plc)	CPRD GOLD
1797	oestrogel 0.06% gel (aventis pharma)	CPRD GOLD
35091	oestrogel pump-pack 0.06% gel (besins healthcare (uk) ltd)	CPRD GOLD
54425	oestrogel pump-pack 0.06% gel (de pharmaceuticals)	CPRD GOLD
57548	oestrogel pump-pack 0.06% gel (sigma pharmaceuticals plc)	CPRD GOLD
56468	oestrogel pump-pack 0.06% gel (waymade healthcare plc)	CPRD GOLD
8465	ovestin tablets 1mg [organon]	CPRD GOLD
7978	pentovis 250microgram capsule (warner lambert uk ltd)	CPRD GOLD
32248	piperazine oestrone sulphate 1.5mg with medroxyprogesterone 10mg tablet	CPRD GOLD
35198	premarin 0.3mg tablets (pfizer ltd)	CPRD GOLD
1331	premarin 0.625mg tablets (pfizer ltd)	CPRD GOLD
2597	premarin 0.625mg/g vaginal cream (pfizer ltd)	CPRD GOLD
774	premarin 1.25mg tablets (pfizer ltd)	CPRD GOLD
52101	premique 0.625mg/5mg tablets (lexon (uk) ltd)	CPRD GOLD
339	premique 0.625mg/5mg tablets (pfizer ltd)	CPRD GOLD
67764	premique 0.625mg/5mg tablets (sigma pharmaceuticals plc)	CPRD GOLD
60932	premique 0.625mg/5mg tablets (waymade healthcare plc)	CPRD GOLD
6492	premique low dose 0.3mg/1.5mg modified-release tablets (pfizer ltd)	CPRD GOLD
1760	prempak 0.625 tablet (wyeth pharmaceuticals)	CPRD GOLD
34749	prempak 1.25 tablet (wyeth pharmaceuticals)	CPRD GOLD
1160	prempak-c 0.625mg/0.15mg tablets (pfizer ltd)	CPRD GOLD
67236	prempak-c 0.625mg/0.15mg tablets (sigma pharmaceuticals plc)	CPRD GOLD
1161	prempak-c 1.25mg/0.15mg tablets (pfizer ltd)	CPRD GOLD
3179	progynova 1mg tablets (bayer plc)	CPRD GOLD
4326	progynova 2mg tablets (bayer plc)	CPRD GOLD
4467	progynova ts 100microgram/24 hr (7.6mg/unit) transdermal patch (schering health care ltd)	CPRD GOLD
9901	progynova ts 100micrograms/24hours transdermal patches (bayer plc)	CPRD GOLD
57590	progynova ts 50 patches (dowelhurst ltd)	CPRD GOLD
4466	progynova ts 50microgram/24hr (3.8mg/unit) transdermal patch (schering health care ltd)	CPRD GOLD
10052	progynova ts 50micrograms/24hours transdermal patches (bayer plc)	CPRD GOLD
8873	quinestradol 250micrograms capsule	CPRD GOLD
3433	sandrena 0.10% gel (organon laboratories ltd)	CPRD GOLD
40643	sandrena 1mg gel sachets (orion pharma (uk) ltd)	CPRD GOLD
57562	sandrena 1mg gel sachets (waymade healthcare plc)	CPRD GOLD
40606	sandrena 500microgram gel sachets (orion pharma (uk) ltd)	CPRD GOLD
256	tibolone 2.5mg tablets	CPRD GOLD

1568	tridestra tablets (orion pharma (uk) ltd)	CPRD GOLD
2452	trisequens forte tablets [novo]	CPRD GOLD
2208	trisequens tablets (novo nordisk ltd)	CPRD GOLD
44494	vagifem 10microgram vaginal tablets (novo nordisk ltd)	CPRD GOLD
49693	vagifem 25microgram vaginal tablets (mawdsley-brooks & company ltd)	CPRD GOLD
2402	vagifem 25microgram vaginal tablets (novo nordisk ltd)	CPRD GOLD
52632	vagifem 25microgram vaginal tablets (waymade healthcare plc)	CPRD GOLD
7388	zumenon 1mg tablets (mylan ltd)	CPRD GOLD
6622	zumenon 2mg tablets (mylan ltd)	CPRD GOLD

Supplementary Table 18: Boby Mass Index (BMI) codes

numunitid	description	Database
122	cm	CPRD Aurum
156	kg	CPRD Aurum
359	Kg/m?	CPRD Aurum
408	cms	CPRD Aurum
568	BMI	CPRD Aurum
657	Kg/mÃÂ²	CPRD Aurum
827	Kgs	CPRD Aurum
907	kg/m^2	CPRD Aurum
22101	Kg/mÂ²	CPRD Aurum
	**medcodeid_term**	**Database**
	weight	CPRD Aurum
	height	CPRD Aurum
	bmi	CPRD Aurum
	Body max index	CPRD Aurum
**enttype**	**enttype_desc**	**Database**
13	weight	CPRD GOLD
14	height	CPRD GOLD
	**data3**	**Database**
	bmi value	CPRD GOLD

Supplementary Table 19: Index of Multiple Deprivation (IMD) codes

imd	description	Database
1	Least deprived	NCRAS HES
2		NCRAS HES
3		NCRAS HES
4		NCRAS HES
5	Most deprived	NCRAS HES
missing	Unknown	NCRAS HES

Supplementary Table 20: Sex codes

sex	description	Database
1	Male	NCRAS HES
2	Female	NCRAS HES
3	Indeterminate	NCRAS HES
4	Unknown	NCRAS HES

Supplementary Table 21: Ethnicity codes

ethnicity	group_1	final_group	database
Bangladesi	Asian	Other	NCRAS HES
Bl_Afric	Black	Other	NCRAS HES
Bl_Carib	Black	Other	NCRAS HES
Bl_Other	Black	Other	NCRAS HES
Chinese	Asian	Other	NCRAS HES
Indian	Asian	Other	NCRAS HES
Mixed	Mixed	Other	NCRAS HES
Oth_Asian	Asian	Other	NCRAS HES
Other	Other	Other	NCRAS HES
Pakistani	Asian	Other	NCRAS HES
White	White	White	NCRAS HES
Unknown	Unknown	Unknown	NCRAS HES
